# Amino Turbo Chirality and Its Asymmetric Control

**DOI:** 10.34133/research.0474

**Published:** 2024-09-19

**Authors:** Ting Xu, Yu Wang, Shengzhou Jin, Anis U. Rahman, Xianghua Yan, Qingkai Yuan, Hao Liu, Jia-Yin Wang, Wenxin Yan, Yinchun Jiao, Ruibin Liang, Guigen Li

**Affiliations:** ^1^ School of Chemistry and Chemical Engineering, Nanjing University, Nanjing 210093, China.; ^2^ Department of Chemistry and Biochemistry, Texas Tech University, Lubbock, TX 79409-1061, USA.; ^3^ School of Pharmacy, Continuous Flow Engineering Laboratory of National Petroleum and Chemical Industry, Changzhou University, Changzhou, Jiangsu 213164, China.; ^4^ School of Chemistry and Chemical Engineering, Key Laboratory of Theoretical Organic Chemistry and Functional Molecular, Ministry of Education, Hunan University of Science and Technology, Xiangtan, Hunan 411201, China.

## Abstract

A series of new targets containing 3 chiral elements of central, orientational, and turbo chirality have been designed and synthesized asymmetrically. The absolute configurations and conformations of these types of chirality were concurrently controlled by using chiral sulfonimine auxiliary and unambiguously determined by x-ray diffraction analysis. These targets include alpha unnatural amino acid derivatives, which may play an important role for drug design, discovery, and development. Three propellers of turbo framework are covalently connected to a chiral C(sp^3^) center via C(sp^2^)–C(sp^3^) bonding along with a C–N axis, while one of them is orientated away from the same carbon chiral center. The turbo or propeller chirality is characterized by 2 types of molecular arrangements of propellers, clockwise (*PPP*) and counterclockwise (*MMM*), respectively. The turbo stereogenicity was found to depend on the center chirality of sulfonimine auxiliary instead of the chiral C(sp^3^) center, i.e., (*S*)- and (*R*)-sulfinyl centers led to the asymmetric formation of *PPP-* and *MMM*-configurations, respectively. Computational studies were conducted on relative energies for rotational barriers of a turbo target along the C–N anchor and the transition pathway between 2 enantiomers meeting our experimental observations. This work is anticipated to have a broad impact on chemical, biomedical, and materials sciences in the future.

## Introduction

The origin of life is mainly about the origin of chirality since it has been found in all living creatures on Earth in forms varying from microscopic living organisms (e.g., helical bacteria) to macroscopic objects (e.g., sea shells) [1–5]. Several types of homochirality widely exist in functional biomolecules, such as DNA/RNA, peptides/proteins, and carbohydrates governing life processes [6–8]. In modern medicine, drug action processes of pharmaceuticals often depend on chirality to impose their potency and selectivity to reduce dosages and unwanted side effects [8–10]. In materials science, CPL (circularly polarized light) research has becoming increasingly active and important since controlling chirality of corresponding compounds and materials plays a crucial role to achieve challenging optoelectronic properties [11–15]. In chemical synthesis, asymmetric synthesis and catalysis have been serving for these areas in the past half a century for generating new chiral small molecules and polymers in higher chemical yields and diastereo- and enantioselectivity [16–43].

**Fig. 1.**
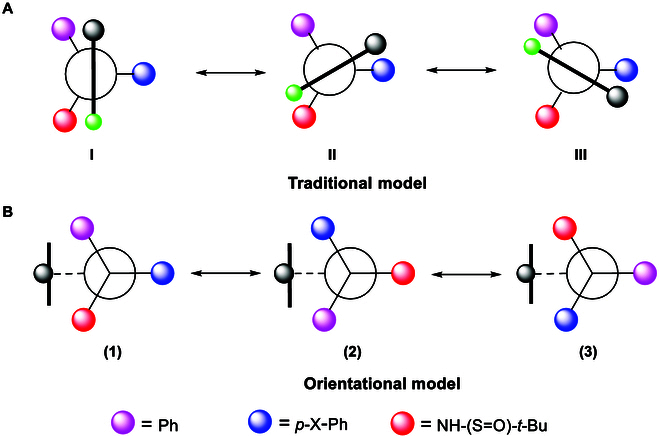
Felkin–Ahn (A) and orientational (B) chirality models.

The discovery and development of new chiral elements is an important aspect of research in organic chemistry and represents one of the most intriguing areas of asymmetric catalysis. So far, there have been following major types of chirality: central [16–18], axial [20–27], spiral [16,21], sandwich (metallic [35–36] and organo [42–47]), and turbo or propeller chirality in small molecules [48–50]; multilayer (rigid helical [13,51] and flexible folding [52,53]) and topological and inherent chirality [54,55] in macro and polymeric molecules. It is worth noting that our recent work on a new chirality, orientational chirality, is uniquely characterized by C(sp^2^)–C(sp^3^) or C(sp)–C(sp^3^) axis-anchored chiral centers and remotely anchored blockers [56–58] (Fig. 1). X-ray structural analysis has proven that individual orientational isomers can be stabilized by through-space functional groups; this enables one *R*- or *S*-chiral center to give 3 orientatiomers by rotating along the C(sp^2^)–C(sp^3^) or C(sp)–C(sp^3^) axis. The orientational model was fundamentally different from the well-known Felkin–Ahn-type or Cram-type models in which chiral C(sp^3^) center and blocking C(sp^2^) carbons are adjacently anchored, leading to 6 energy barriers during rotating operation. However, in orientational chirality, there are 3 energy barriers for either (*R*)- or (*S*)-stereogenicity (Fig. 1). This is due to the fact that there exists a the steric dialog between the chiral C(sp^3^) center and its remotely anchored blocker.

**Fig. 2.**
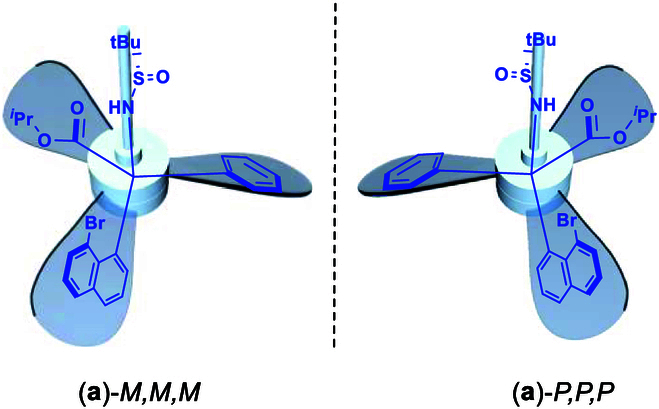
Turbo chiral targets via 2 rotational operations along the C–N axis [orientational chirality was also shown by Ph being pushed away from Br].

**Fig. 3.**
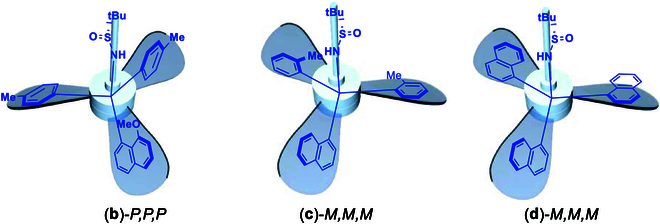
Turbo chiral molecules centered by achiral carbon.

During our ongoing effort on seeking new orientational molecules, we now found that the chiral or achiral C(sp^3^) center can be surrounded by 3 planar moieties with 2 types of arrangements of clockwise and counterclockwise fashions, which belong turbo or propeller chirality (Fig. 2). This turbo chirality originated from a chiral sulfur center of sulfinyl amide, which controls 3 chiral elements at the same time: C(sp^3^) central, orientational, and turbo chirality. Surprisingly, the turbo chirality was solely controlled by the chiral sulfur center without being affected by the chiral C(sp^3^) center as proven by generating achiral C(sp^3^) centers as shown by x-ray diffraction analysis (Fig. 3). It is worth mentioning that over 75% of drugs contain amino functionality and more and more drugs have chiral center. The turbo amino chirality would provide more opportunities for chiral drug design and development in the future. Herein in this report, we would like to present our preliminary results on this new molecular framework.

## Results

The present work was initiated by our design and synthesis of chiral unnatural amino acids with orientational chirality for peptide and protein research [8,56–58]. Introducing carboxylic ester and amine functionalities to the C(sp^3^) center would serve for this purpose. It is well known that in traditional amino acids, one (*R*)- or (*S*)-chiral carbon center only corresponds to one chiral amino acid isomer. However, the number of chiral amino acid isomers would be increased 3 times more if their orientational chirality is taken into account. The use of naphthyl ring as the structural template for the synthesis of orientational amino acid derivatives would serve for this purpose, which has been proven to be successful in our previous similar design. Obviously, the substitution of hydrogen atom on position 1 of naphthyl ring with larger moieties, such as Me, MeO, and Br, is anticipated to impose steric effects, so 1 of the 3 groups of the chiral C(sp^3^) center on naphthyl position 8 is pushed away from them. Bromine substitution is the first case we investigated since we believe that heavy atom effect would benefit forming crystals for resulting products. Fortunately, based on this analysis, high-quality crystals of 2 individual enantiomers of the corresponding products were obtained smoothly. Their x-ray diffraction analysis proved that the phenyl ring of chiral carbon center is pushed away from the Br blocker, while isopropyl ester and *N*-sulfinyl groups are placed on each side of naphthyl ring in each case [(a)-*M,M,M* and (a)-*P,P,P* in Fig. 2].

**Fig. 4.**
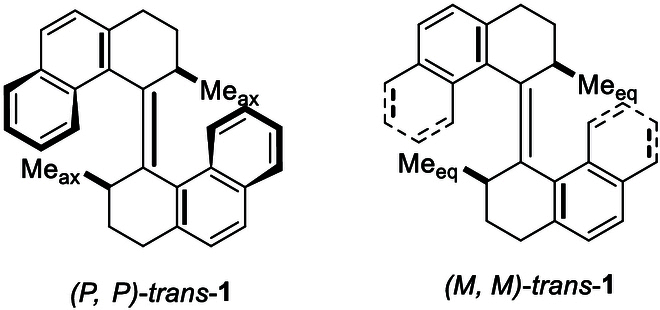
Optical switching molecules centered by alkene.

The structural characteristics and nomenclature of the present turbo or propeller chirality directly benefits from known chiral targets, especially those of chiroptical switch molecules invented by the Nobel laureate Ben L. Feringa [59]. In those cases, alkenes are used as a centrally overcrowded anchor and connected by 2 propeller blades with 2 chiral centers (Fig. 4). However, in our new targets, a chiral carbon center serves as a central anchor connected by 3 propellers and an amino moiety with chiral sulfur. As shown in Fig. 3, the chiral carbon center is unnecessary to achieve turbo chiral arrangement since 2 or 3 identical aromatic rings can be attached onto it, still showing 3 propellers of clockwise or anticlockwise arrangements depending on the chirality of amino protection auxiliary.

**Fig 5.**
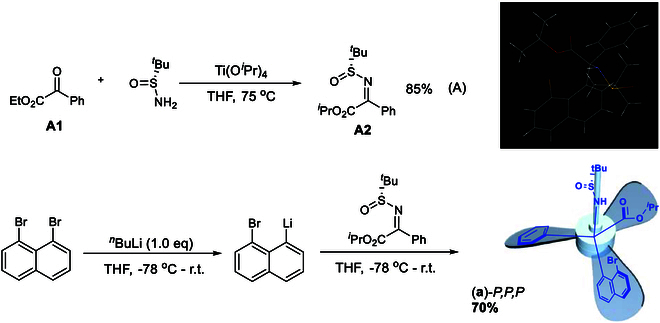
Asymmetric synthesis of turbo chiral (a)-*P,P,P* and its x-ray structure.

Asymmetric synthesis of turbo products is represented by assembling isopropyl 2-(8-bromonaphthalen-1-yl)-2-(((*S*)-*tert*-butylsulfinyl)amino)-2-phenylacetate [(**a**)-*M,M,M* in Figs. 2 and 5]. It was started by preparing the first building block **A2** through dehydration of (*R*)-2-methylpropane-2-sulfinamide with ethyl 2-oxo-2-phenylacetate **A1** by the use of Ti(OEt)_4_ in dried tetrahydrofuran (THF) at 75 °C and then to room temperature to give isopropyl (*R,Z*)-2-((*tert*-butylsulfinyl)imino)-2-phenylacetate **A2** in a chemical yield of 85% (equation A, Fig. 5) [60,61]. 1,8-Dibromonaphthalene was pre-converted to its mono-lithium reagent by treating with butyllithium at −78 °C, which then proceeded electrophilic addition in situ to give isopropyl (*R,Z*)-2-((tert-butyl sulfinyl)imino)-2-phenylacetate **A2** at the same temperature for 2 h. Chromatographic purification afforded the final a single isomeric product of isopropyl 2-(8-bromonaphthalen-1-yl)-2-(((*R*)-*tert*-butyl sulfinyl) amino)-2-phenylacetate (a)-*P,P,P* in 70% yield (equation B, Fig. 5). The absolute configuration of this isomeric product has been unambiguously proven by its x-ray diffraction analysis as shown in Fig. 5.

**Fig. 6.**
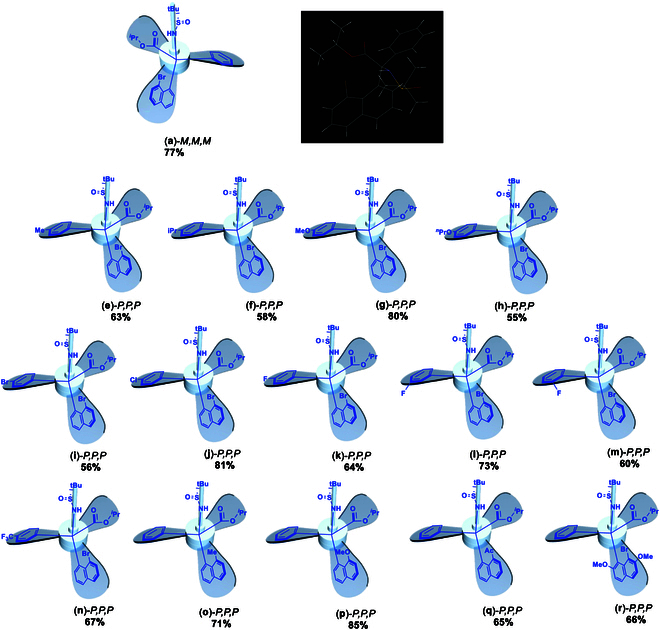
Scope of asymmetric synthesis of turbo chiral compounds.

The similar asymmetric assembly of the opposite enantiomer of(**a**)-*P,P,P,*isopropyl 2-(8-bromonaphthalen-1-yl)-2-(((*S*)-*tert*-butylsulfinyl)amino)-2-phenylacetate [(**a**)-*M,M,M*] was performed by following the above procedure to give a chemical yield of 77% for the key step that is nearly identical to that of the former (Fig. 6). Its absolute configuration was also determined by x-ray diffraction analysis as shown in Fig. 6. Two x-ray structures clearly indicate turbo arrangements surrounding the chiral carbon center, showing that the (*S*)-carbon, (*R*)-*tert*-butylsulfinyl group corresponds to clockwise and (*R*)-carbon, (*S*)-*tert*-butylsulfinyl group to *anti*-clockwise (Figs. 5 and 6).

**Fig. 7.**
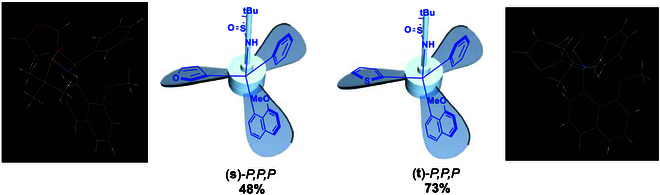
Turbo chirality targets with heterocycles.

Along with the generation of isomeric product (**a**)-*P,P,P*, we expend this approach to a series of other turbo isomeric products by retaining 8-bromonaphthalen-1-yl substructure and changing Ph ring to other aromatic counterpart at first (Fig. 6). Symmetrically substituted 4-methyl and isopropyl phenyl substrates afforded turbo isomeric products, (**e**)-*P,P,P* and (**f**)-*P,P,P*, in chemical yields of 63% and 58%, respectively. The use of stronger electron-donation groups, MeO-[(**g**)-*P,P,P*] and nPro-[(**h**)-*P,P,P*] chemical yields of 80% and 55%, were obtained with a much higher yield for the former case but an almost identical yield for the latter. Three para-halogenated aryl (4-Br-, 4-Cl, and 4-F) and one ortho- and meta-F-Ph all resulted in turbo chiral products in chemical yields ranging from 56% to 81% [(**i**)-*P,P,P* to (**m**)-*P,P,P*; Fig. 6]. One strong electron-withdrawing group, 4-CF_3_-Ph-attached aryl substrate, also worked well to give 67% yield ((**n**)-*P,P,P*). Second, the substrate change was made on the remaining 8-bromonaphthalen-1-yl substructure by replacing bromine with 3 other groups, Me-, MeO-, and Ac. Chemical yields of 71%, 85%, and 65% were achieved, respectively. The last substrate modification was made by introducing 2 MeO groups onto positions 2 and 7 of 8-bromonaphthalen-1-yl substructure. The expected turbo chiral product, isopropyl 2-(8-bromo-2,7-dimethoxynaphthalen-1-yl)-2-(((R)-*tert*-butylsulfinyl)amino)-2-phenylacetate (**r**)-*P,P,P*, was generated in 66% yield. Third, the substrate change was made by changing both iso-propyl ester to heterocycles of furan and thiophen and 8-bromonaphthalen-1-yl to 8-methoxylnaphthalen-1-yl counterpart. Individual enantiomers of (*R*)-*N*-(furan-3-yl(8-methoxynaphthalen-1-yl)(phenyl)methyl)-2-methylpropane-2-sulfinamide [(**s**)-*P,P,P*; Fig. 7] and (*R*)-*N*-((8-methoxynaphthalen-1-yl)(phenyl)(thiophen-2-yl)methyl)-2-methylpropane-2-sulfinamide [(**t**)-*P,P,P*; Fig. 7] were obtained in yields of 48% and 73%, respectively. More importantly, x-ray diffraction analysis revealed that both these turbo products display obvious clockwise absolute configurations, and both propeller blades of furan and thiophen rings are directed down-side away from sulfinyl group on chiral carbon center (Fig. 7).

To investigate whether chiral sulfur or carbon center control the turbo chirality predominantly, we utilized 2 and/or 3 identical aromatic groups to be anchored onto the central sp^3^-carbon by using (*R*)-*N*-(di-tolylmethylene)-2-methylpropane-2-sulfinamide and (*S*)-*N*-(di(naphthalen-1-yl)methylene)-2-methylpropane-2-sulfinamide as electrophilic acceptors. These receptors that reacted with corresponding ArLi to give (*R*)-*N*-(di-*p*-tolylmethylene)-2-methylpropane-2-sulfinamide, (*S*)-N-(di-o-tolylmethylene)-2-methylpropane-2-sulfinamide, and (*S*)-N-(di(naphthalen-1-yl)methylene)-2-methylpropane-2-sulfinamide were synthesized in chemical yields of 47% [(**b**)-*P,P,P*], 60% [(**c**)-*M,M,M*], and 55% [(**d**)-*M,M,M*], respectively. To our pleasure, we were able to get good-quality crystals of these 3 turbo chiral products for x-ray structural analysis. Their x-ray structures confirmed that absolute turbo chirality depends on configuration of sulfur of sulfinamide instead of their sp^3^ carbon centers. This means that (*R*)-*N*-propane-2-sulfinyl auxiliary leads to the formation of *P,P,P*-turbo chirality and (*S*)-*N*-propane-2-sulfinyl auxiliary results in the opposite *M,M,M*-turbo chirality. This conclusion can also be proven by the other 4 x-ray structures of 2 pairs of enantiomers controlling 2 opposite chiral auxiliaries as shown in Figs. 5 to 7.

**Fig. 8.**
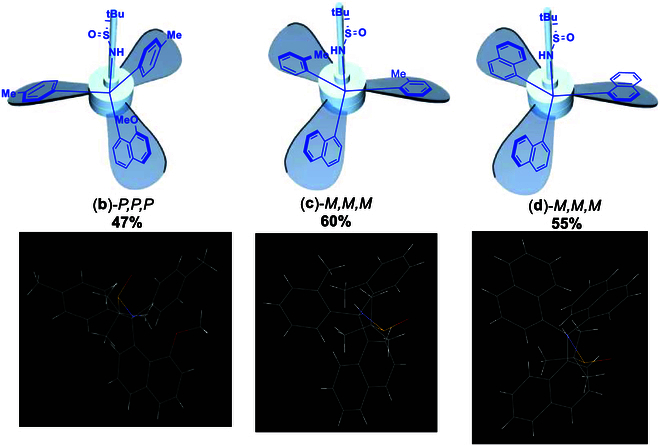
Turbo chirality targets with achiral C(sp^3^)-center.

**Fig. 9.**
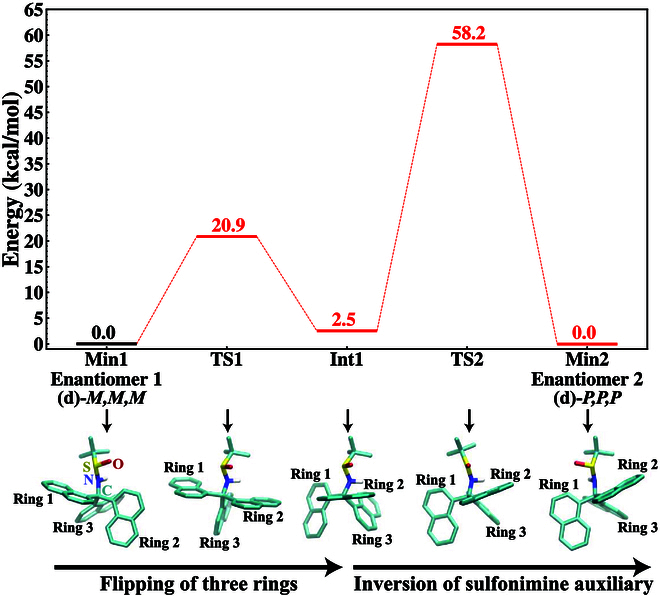
Energy diagram for the reaction pathway from Enantiomer 1 to Enantiomer 2, which are mirror images of each other. The first stage of the pathway is the simultaneous flipping of the 3 naphthalene rings, overcoming transition state 1 (TS1) and resulting in an intermediate (Int1). The second stage is the inversion of the sulfonimine auxiliary, overcoming transition state 2 (TS2) and resulting in an Enantiomer 2. Structures of the stationary points along the pathway (minima and transition states) are illustrated below the energy diagram. Key functional groups are labeled, including the central atoms (C, N, S, O atoms) in the axis of the ring-flipping motion. All energies are evaluated using the B3LYP-D3/def2-TZVP method at the stationary points optimized with the B3LYP-D3/def2-SVP method. The zero point energy (ZPE) corrections have been included at the B3LYP-D3/def2-SVP level of theory at the optimized stationary points. The higher energy of Int1 than Enantiomer 1 and 2 indicates that the sulfonimine auxiliary thermodynamically controls the turbo chirality of the 3 naphthalene rings. Also, the considerable kinetic barriers for both stages of the transition contribute to the kinetic stability of the 2 enantiomers.

We further employed quantum mechanics (QM) calculations at the density functional theory (DFT) level of theory to characterize the potential energies of stationary points (minima and transition states) along the minimum energy pathway (MEP) connecting (**d**)-*M,M,M* and (**d**)-*P,P,P* [62–72] (Fig. 8). These 2 enantiomers are referred to as Enantiomers 1 and 2 below, respectively. The results are summarized in Fig. 9. The computational results support the experimental findings that the center chirality of the sulfonimine auxiliary thermodynamically controls the turbo chirality of the 3 naphthalene rings. In particular, comparing Intermediate 1 and Enantiomer 1, which share the same center chirality of the sulfonimine auxiliary but differ in the turbo chirality of the 3 naphthalene rings, Intermediate 1 is 2.5 kcal/mol higher in energy due to changing the turbo chirality of the 3 rings. In other words, the lower energy of Enantiomer 1 perhaps arises from the more favorable van der Waals interaction between the sulfonimine auxiliary and the 3 hydrophobic naphthalene rings oriented in this chiral configuration. Thus, at equilibrium, the chirality of the sulfonimine auxiliary results in a higher population of the *MMM* turbo chiral configuration in Enantiomer 1 than the *PPP* configuration observed in Intermediate 1.

The transition between the 2 chiral configurations of 3 naphthalene rings is also kinetically slow. The TS separating Enantiomer 1 and Intermediate 1 has a high energy of 20.9 kcal/mol, implying a slow rate for changing the turbo chirality of the 3 rings, contributing to the kinetic stability of Enantiomer 1. The inversion of the central chirality of the sulfonimine auxiliary converts Intermediate 1 to Enantiomer 2. Enantiomer 2 is the mirror image of Enantiomer 1 and thus has the same energy as the latter. Starting from Intermediate 1, the inversion of the sulfonimine auxiliary’s chirality needs to overcome a high kinetic barrier of 55.7 kcal/mol, leading to a significant overall kinetic barrier of 58.2 kcal/mol separating Enantiomers 1 and 2.

**Fig. 10.**
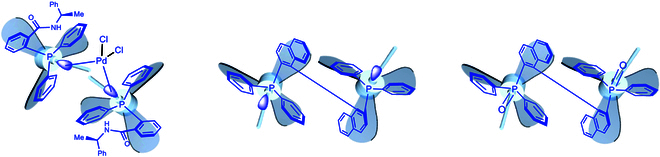
Turbo chirality with 2 units of triple propeller blades of chiral ligands and complex.

The extended computational work is currently being extended to other atom-centered turbo molecular frameworks in our laboratories. It is worth mentioning that although tri- or tetra-aromatic rings surrounding P- and C-centered compounds have been widely reported in literature [21–22,42,73–88], their turbo chirality patterns have not been paid attention for a while until recently when several groups were involved in this research (Fig. 10 and Supplementary Materials) [48–50,83–86]. In addition, we also found that turbo chirality exists in diaryl ethers with 2 propeller blades [88–91] and in multiple aryl ring-anchored structures with tri or tetra propeller blades ([87,92,93] and Supplementary Materials).

## Discussion

We have designed and synthesized new chiral targets containing central, orientational, and turbo chirality surrounding a C–N axis. The chirality is efficiently controlled by sulfonimine auxiliary via asymmetric nucleophilic carbonyl addition reaction. The resulting configurations and conformations have been unambiguously confirmed by x-ray diffraction analysis. The turbo atropisomers are characterized by 2 types of molecular clockwise and counterclockwise arrangements of structural blades. The absolute *PPP-* and *MMM*-stereogenicity was proven to depend on the center chirality of sulfonimine auxiliary, i.e., (*S*)- and (*R*)-sulfinyl centers led to the asymmetric formation of complete *PPP-* and *MMM*-configurations, respectively, regardless of the center chirality of C(sp^3^) joint. This was confirmed by attaching 2 or 3 identical aromatic blades onto the C(sp^3^) joint of the C–N axis. Computational studies were performed to characterize the energy of the intermediate state and the barriers along the MEP between 2 enantiomers of (**d**). The computational results support our experimental finding that the turbo chirality of the compound is thermodynamically controlled by the center chirality of the sulfonimine auxiliary. Meanwhile, the chirality inversion of the sulfonimine auxiliary is the rate-limiting step for the transition between the *PPP*- and *MMM-*configurations. The high barriers along the reaction pathway prevent facile transition between them, allowing for the separation of distinct kinetically stable enantiomers in the experiment. The present turbo chirality work would be anticipated by enhancing a new stereochemistry topic and to have a broad impact on chemical, biomedical, and material sciences in the future.

## Materials and Methods

Unless otherwise stated, all reactions were magnetically stirred and conducted in oven-dried glassware in anhydrous solvents under Ar, applying standard Schlenk techniques. Solvents and liquid reagents, as well as solutions of solid or liquid reagents were added via syringes, stainless steel, or polyethylene cannulas through rubber septa or through a weak Ar counterflow. Solvents were removed under reduced pressure at 40 to 65 °C using a rotavapor. All given yields are isolated yields of chromatographic and NMR spectroscopic materials. All commercially available chemicals were used as received without further purification.

^1^H and ^13^C nuclear magnetic resonance (NMR) spectra were recorded in CDCl_3_ on 400-MHz instruments with trimethylsilyl (TMS) as internal standard. For referencing of the ^1^H NMR spectra, the residual solvent signal (δ = 7.26 ppm for CDCl_3_) was used. In the case of the ^13^C NMR spectra, the signal of solvent (δ = 77.0 ppm for CDCl_3_) was used. Chemical shifts (δ) were reported in ppm with respect to TMS. Data are represented as follows: chemical shift, multiplicity (s = singlet, d = doublet, t = triplet, m = multiplet), coupling constant (*J*, Hz), and integration. Optical rotations were measured with a Rudolph Research Analytical APIV/2 W Polarimeter at the indicated temperature with a sodium lamp. Measurements were performed in a 2-ml vessel with the concentration unit of g/100 ml in the corresponding solvents.

 The details of computational methods are included in the Supplementary Materials, which include the selection of initial geometries, the QM level of theory, and the optimization of the stationary points along the reaction pathway.

## Data Availability

All data are available in the manuscript or Supplementary Materials.

## Supplementary Materials

Supplementary 1 Supplementary Text Figs. S1 to S48 Table S1 Computational Methods
