# Supplementary material for: Amino Turbo Chirality and Its Asymmetric Control
Source: Research (Wash D C). 2024 Sep 19;7:0474. doi: 10.34133/research.0474 (PMC11411161; doi:10.34133/research.0474)
Supplement: Supplementary 1 — Supplementary Text Figs. S1 to S48 Table S1 Computational Methods [file research.0474.f1.pdf]

Supporting Information

Amino Turbo Chirality and Its Asymmetric Control

Ting Xu,[a] Yu Wang,[a] Shengzhou Jin,[a] Anis U. Rahman,[b] Xianghua Yan,[b] Qingkai Yuan,[b] Hao Liu,[b] Jia-Yin Wang,[c] Wenxin Yan,[d] Yinchun Jiao,[d] Ruibin Liang,[*,b] and Guigen Li[*,a,b]

[a] *School of Chemistry and Chemical Engineering, Nanjing University, Nanjing, 210093, China.*
[b] *Department of Chemistry and Biochemistry, Texas Tech University, Lubbock, Texas 79409-1061, USA.*
[c] *School of Pharmacy, Continuous Flow Engineering Laboratory of National Petroleum and Chemical Industry, Changzhou University, Changzhou, Jiangsu 213164, China,*
[d] *School of Chemistry and Chemical Engineering, Key Laboratory of Theoretical Organic Chemistry and Functional Molecular, Ministry of Education, Hunan University of Science and Technology, Xiangtan, Hunan 411201, China.*

[*]Correspondence should be addressed to Ruibin Liang and Guigen Li: rliang@ttu.edu *(RL)* guigen.li@ttu.edu, guigenli@nju.edu.cn (GL)

Context

1. General Procedure for the Synthesis of (**a**)-*P*,*P*,*P* - (**n**)-*P*,*P*,*P* S2-4

2. Procedure for the Synthesis of (**s**)-*P*,*P*,*P* - (**d**)-*M*,*M*,*M* S4-8

3. Characterization Data of Compounds (**a**)-*P*,*P*,*P* - (**t**)-*P*,*P*,*P* S8-16

4. NMR Spectra of Compounds (**a**)-*P*,*P*,*P* - (**t**)-*P*,*P*,*P* S17-37

5. X-ray Single-crystal Data for Compound (**a**)-*P*,*P*,*P*, (**a**)-*M*,*M*,*M*, (**b**)-*P*,*P*,*P*, (**c**)-*M*,*M*,*M*, (**d**)-*M*,*M*,*M*, (**s**)-*P*,*P*,*P*, (**t**)-*P*,*P*,*P* S38-44

6. Other atom-centered chiral turbo frameworks (A few examples) S45-49

EXPERIMENTAL SECTION

For general Information, all melting points are uncorrected. The NMR spectra were recorded in $CDCl_3$ on a 400 MHz instrument with TMS as the internal standard. Chemical shifts (δ) are reported in ppm with respect to TMS. Data are represented as follows: chemical shift, multiplicity (s = singlet, d = doublet, t = triplet, m = multiplet), coupling constant (J, Hz), and integration. High-resolution mass spectrometry (HRMS) analyses were carried out using a time-of-flight mass spectrometry (TOF-MS) instrument with an electrospray ionization (ESI) source. X-ray crystallographic analysis was performed with a SMART CCD and a P4 diffractometer. All commercially sourced starting materials were used without further purification.

1. General Procedure for the Synthesis of (a)-*P,P,P* - (n)-*P,P,P*.

Asymmetric Synthesis of Turbo Chiral Targets

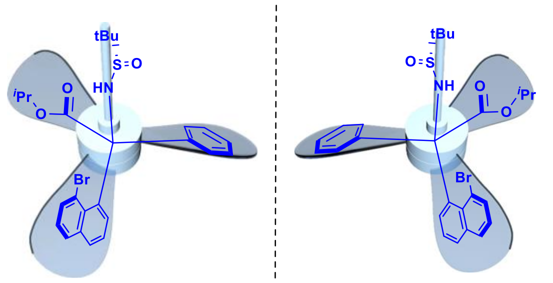

(**a**)-*M,M,M* (**a**)-*P,P,P*

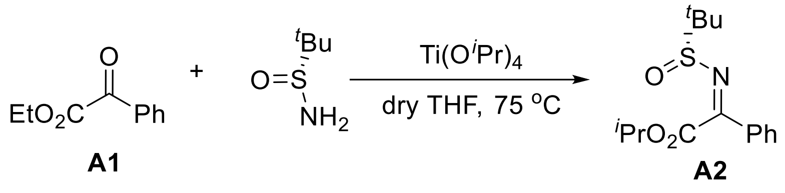

Stir a mixture of (R)-^{t}BuSONH$_2$ (363 mg, 3.0 mmol), ethyl 2-oxo-2-phenylacetate **A1** (356 mg, 2.0 mmol) and Ti(O^{i}Pr)$_4$ (1.72 g, 6.0 mmol) in dry THF (15 mL) at 75 °C for 16 h. Cool the reaction vial to room temperature, and hydrolyze the resulting mixture with brine. The liquid was separated with EA and water, and the organic phase was taken. Removal of solvent under reduced pressure and purified by column

chromatography (PE:EA = 20:1) to give the yellow solid isopropyl (R,Z)-2-((tert-butylsulfinyl)imino)-2-phenylacetate **A2** in 85% yield (502 mg, 1.7 mmol).

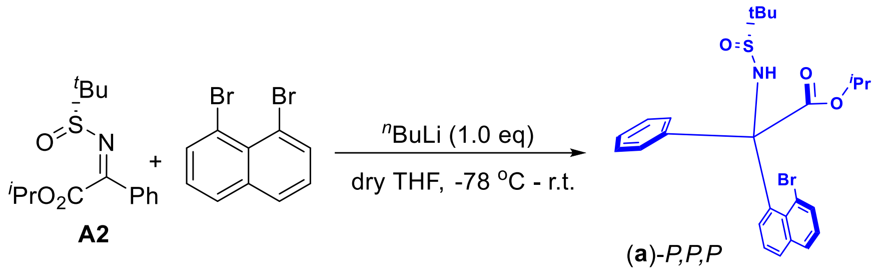

To a solution of 1,8-dibromonaphthalene (286 mg, 1 mmol) in dry THF at -78 °C was added *n*-butyllithium (0.625 mL, 1.6 M) as a solution in hexanes. The resulting solution was stirred for 1 hour, a solution of isopropyl (R,Z)-2-((tert-butylsulfinyl)imino)-2-phenylacetate **A2** (296 mg, 1 mmol) in dry THF was slowly added via syringe. Stirring was continued at -78 °C for 2 hour, then the solution was allowed to warm to room temperature and saturated aqueous NH_4Cl was added dropwise. The organic phase was extracted with EA and dried over anhydrous $MgSO_4$. Chromatography (PE:EA= 3:1) afforded the diastereomerically pure isopropyl isopropyl 2-(8-bromonaphthalen-1-yl)-2-(((R)-tert-butylsulfinyl)amino)-2-phenylacetate (**a**)-*P,P,P* in 70% yield (351 mg, 0.7 mmol).

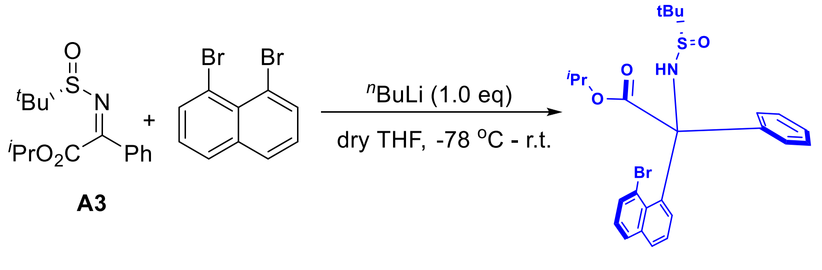

The product of isopropyl 2-(8-bromonaphthalen-1-yl)-2-(((S)-tert-butylsulfinyl)amino)-2-phenylacetate **1b** (77% yield, 386 mg, 0.77 mmol) was synthesized in the same way as above. As shown in Figure 1, the absolute configurations of the isomeric products with two turbo chiral compounds have been unambiguously determined by X-ray crystallographic analysis.

We have synthesized a series of turbo chiral compounds with the use of ^{n}BuLi (1.0 eq), 1-

bromonaphthalene bearing various substituents in 8-position underwent nucleophilic addition with isopropyl (R,Z)-2-((tert-butylsulfinyl)imino)-2-phenylacetate **A2** in moderate yield from 55% to 81% ((**e**)-*P,P,P* - (**n**)-*P,P,P*), showing that the electronic and steric nature of the naphthalene ring hardly affect the reaction

2. Procedure for the Synthesis of (s)-*P,P,P* - (d)-*M,M,M*

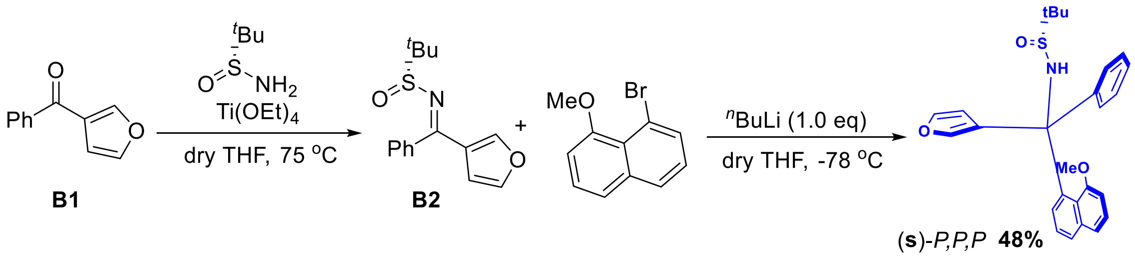

Stir a mixture of (R)-^{t}BuSONH$_2$ (485 mg, 4.0 mmol), furan-3-yl(phenyl)methanone (344 mg, 2.0 mmol) **B1** and Ti(OEt)$_4$ (1.692 g, 6.0 mmol) in dry THF (15 mL) at 75 °C for 18 h. The resulting mixture was hydrolyzed with brine. The liquid was separated with EA and water, and the organic phase was taken. Removal of solvent under reduced pressure and purified by column chromatography (PE:EA = 10:1) to give the yellow oil (R,E)-N-(furan-3-yl(phenyl)methylene)-2-methylpropane-2-sulfinamide **B2** in 56% yield (308 mg, 1.12 mmol).

To a solution of 1-bromo-8-methoxynaphthalene (238 mg, 1 mmol) in dry THF at -78 °C was added *n*-butyllithium (0.625 mL, 1.6 M) as a solution in hexanes. The resulting solution was stirred for 1 hour, a solution of (R,E)-N-(furan-3-yl(phenyl)methylene)-2-methylpropane-2-sulfinamide (276 mg, 1 mmol) in dry THF was slowly added via syringe. Stirring was continued at -78 °C for 4 hours, then the solution was allowed to warm to room temperature and saturated aqueous NH$_4$Cl was added dropwise. The organic phase was extracted with EA and dried over anhydrous MgSO$_4$. Chromatography (PE:EA= 3:1) afforded the diastereomerically white solid (R)-N-((S)-furan-3-yl(8-methoxynaphthalen-1-yl)(phenyl)methyl)-2-methylpropane-2-sulfinamide (**s**)-*P,P,P* in 48% yield (206 mg, 0.48 mmol).

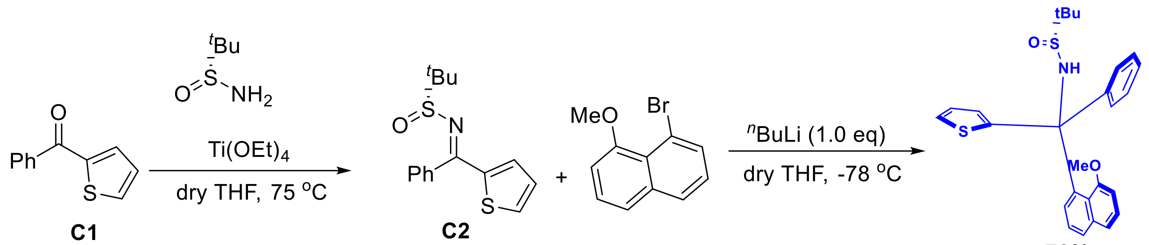

C1 **C2** (**t**)-*P,P,P* **73%**

Stir a mixture of (R)-^{t}BuSONH$_2$ (480 mg, 4.0 mmol), phenyl(thiophen-2-yl)methanone **C1** (375 mg, 2.0 mmol) and Ti(OEt)$_4$ (1.71 g, 6.0 mmol) in dry THF (15 mL) at 75 °C for 18 h. The resulting mixture was hydrolyzed with brine. The liquid was separated with EA and water, and the organic phase was taken. Removal of solvent under reduced pressure and purified by column chromatography (PE:EA = 10:1) to give the yellow oil (R,E)-2-methyl-N-(phenyl(thiophen-2-yl)methylene)propane-2-sulfinamide **C2** in 80% yield (472 mg, 1.62 mmol).

To a solution of 1-bromo-8-methoxynaphthalene (236 mg, 1 mmol) in dry THF at -78 °C was added *n*-butyllithium (0.60 mL, 1.6 M) as a solution in hexanes. The resulting solution was stirred for 1 hour, a solution of (R,E)-2-methyl-N-(phenyl(thiophen-2-yl)methylene)propane-2-sulfinamide **C2** (276 mg, 1 mmol) in dry THF was slowly added via syringe. Stirring was continued at -78 °C for 2 hours, then the solution was allowed to warm to room temperature and saturated aqueous NH_4Cl was added dropwise. The organic phase was extracted with EA and dried over anhydrous $MgSO_4$. Chromatography (PE: EA= 3:1) afforded the diastereomerically white solid (R)-N-((8-methoxynaphthalen-1-yl)(phenyl)(thiophen-2-yl)methyl)-2-methylpropane-2-sulfinamide (**t**)-*P,P,P* in 73% yield (326 mg, 0.73 mmol).

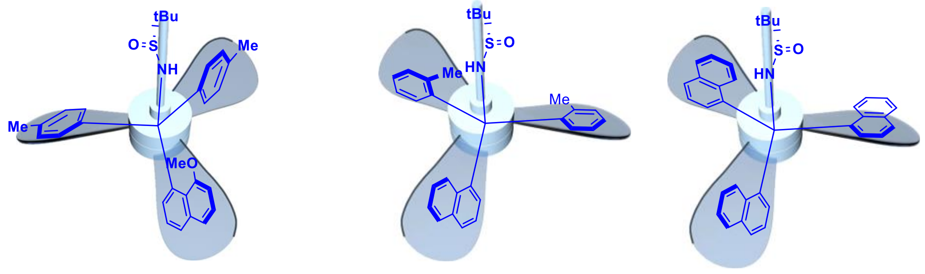

(**b**)-*P,P,P* **47%** (**c**)-*M,M,M* **60%** (**d**)-*M,M,M* **55%**

To investigate whether chiral sulfur or carbon center control the turbo chirality predominently, we utilized two and/or three identical aromatic group to be anchored onto the central sp^3-carbon by using (*R*)-*N*-(di-tolylmethylene)-2-methylpropane-2-sulfinamide and (*S*)-*N*-(di(naphthalen-1-yl)methylene)-2-methylpropane-2-sulfinamide as electrophilic acceptors. These receptors reacted with corresponding ArLi to give (*R*)-*N*-(di-*p*-tolylmethylene)-2-methylpropane-2-sulfinamide, (*S*)-N-(di-o-tolylmethylene)-2-methylpropane-2-sulfinamide and (*S*)-N-(di(naphthalen-1-yl)methylene)-2-methylpropane-2-sulfinamide were synthesized in chemical yields of 47% [(**b**)-*P,P,P*], 60% [(**c**)-*M,M,M*] and 55% [(**d**)-*M,M,M*], respectively.

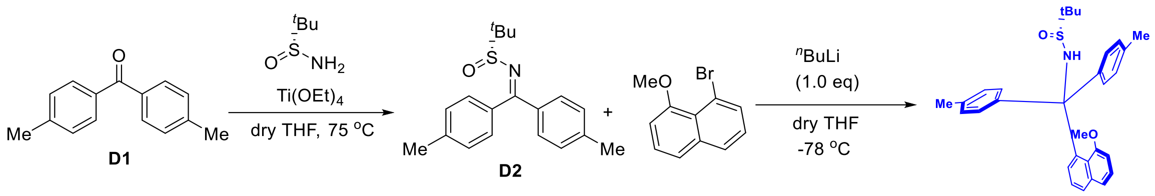

(**b**)-*P,P,P* **47%**

Stir a mixture of (R)-^{t}BuSONH$_2$ (490 mg, 4.0 mmol), di-p-tolylmethanone **D1** (421 mg, 2.0 mmol) and Ti(OEt)$_4$ (1.7 g, 6.0 mmol) in dry THF (15 mL) at 75 °C for 18 h. The resulting mixture was hydrolyzed with brine. The liquid was separated with EA and water, and the organic phase was taken. Removal of solvent under reduced pressure and purified by column chromatography (PE:EA = 20:1) to give the yellow oil (R)-N-(di-p-tolylmethylene)-2-methylpropane-2-sulfinamide **D2** in 86% yield (539 mg, 1.71 mmol).

To a solution of 1-bromo-8-methoxynaphthalene (152 mg, 0.64 mmol) in dry THF at -78 °C was added *n*-butyllithium (0.4 mL, 1.6 M) as a solution in hexanes. The resulting solution was stirred for 1 hour, a solution of (R)-N-(di-p-tolylmethylene)-2-methylpropane-2-sulfinamide **D2** in 86% yield (201 mg, 0.64 mmol) in dry THF was slowly added via syringe. Stirring was continued at -78 °C for 12 hours, then the solution was allowed to warm to room temperature and saturated aqueous NH$_4$Cl was added dropwise. The organic phase was extracted with EA and dried over anhydrous MgSO$_4$. Chromatography (PE: EA= 3:1 to 1:1) afforded the diastereomerically white solid (R)-N-((8-methoxynaphthalen-1-yl)di-p-tolylmethyl)-2-methylpropane-2-sulfinamide (**b**)-*P,P,P* in 47% yield (142 mg, 0.3 mmol).

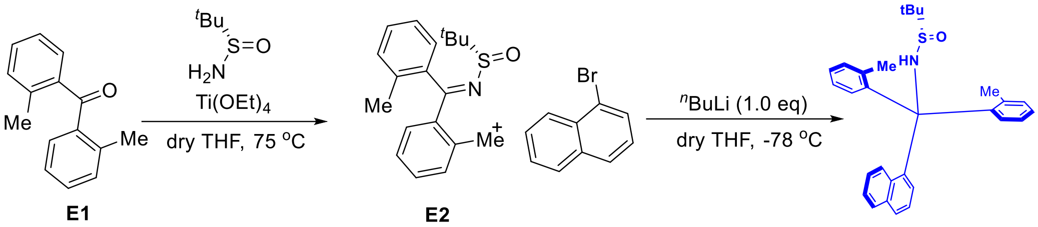

(**c**)-*M,M,M* **60%**

Stir a mixture of (S)-^{t}BuSONH$_2$ (488 mg, 4.0 mmol), di-o-tolylmethanone **E1** (422 mg, 2.0 mmol) and Ti(OEt)$_4$ (1.68 g, 6.0 mmol) in dry THF (15 mL) at 90 °C for 28 h. The resulting mixture was hydrolyzed with brine. The liquid was separated with EA and water, and the organic phase was taken. Removal of solvent under reduced pressure and purified by column chromatography (PE:EA = 15:1) to give the yellow oil (S)-N-(di-o-tolylmethylene)-2-methylpropane-2-sulfinamide **E2** in 56% yield (308 mg, 1.12 mmol).

To a solution of 1-bromonaphthalene (208 mg, 0.16 mmol) in dry THF at -78 °C was added *n*-butyllithium (0.1 mL, 1.6 M) as a solution in hexanes. The resulting solution was stirred for 1 hour, a solution of (S)-N-(di-o-tolylmethylene)-2-methylpropane-2-sulfinamide **E2** (50 mg, 0.16 mmol) in dry THF was slowly added via syringe. Stirring was continued at -78 °C for 6 hours, then the solution was allowed to warm to room temperature and saturated aqueous NH$_4$Cl was added dropwise. The organic phase was extracted with EA and dried over anhydrous MgSO$_4$. Chromatography (PE: EA= 3:1 to 1:1) afforded the diastereomerically white solid (S)-2-methyl-N-(naphthalen-1-yldi-o-tolylmethyl)propane-2-sulfinamide (**c**)-*M,M,M* in 60% yield (42 mg, 0.1 mmol).

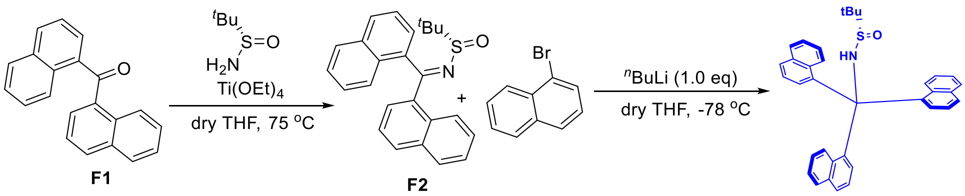

(**d**)-*M,M,M* **55%**

Stir a mixture of (S)-^{t}BuSONH$_2$ (484 mg, 4.0 mmol), di(naphthalen-1-yl)methanone **F1** (565 mg, 2.0 mmol) and Ti(OEt)$_4$ (1.692 g, 6.0 mmol) in dry THF (15 mL) at 90 °C for 48 h. The resulting mixture was hydrolyzed with brine. The liquid was separated with EA and water, and the organic phase was taken. Removal

of solvent under reduced pressure and purified by column chromatography (PE:EA = 15:1) to give the yellow solid (S)-N-(di(naphthalen-1-yl)methylene)-2-methylpropane-2-sulfinamide **F2** in 21% yield (161 mg, 0.42 mmol).

To a solution of 1-bromonaphthalene (32.8 mg, 0.16 mmol) in dry THF at -78 °C was added *n*-butyllithium (0.1 mL, 1.6 M) as a solution in hexanes. The resulting solution was stirred for 1 hour, a solution of (S)-N-(di(naphthalen-1-yl)methylene)-2-methylpropane-2-sulfinamide **F2** (61.8 mg, 1 mmol) in dry THF was slowly added via syringe. Stirring was continued at -78 °C for 2 hours, then the solution was allowed to warm to room temperature and saturated aqueous NH_4Cl was added dropwise. The organic phase was extracted with EA and dried over anhydrous $MgSO_4$. Chromatography (PE: EA= 1:1) afforded the diastereomerically white solid (S)-2-methyl-N-(tri(naphthalen-1-yl)methyl)propane-2-sulfinamide (**d**)-*M,M,M* in 55% yield (44.9 mg, 0.09 mmol).

3. Characterization Data of Compounds (a)-*P,P,P* - (t)-*P,P,P*

isopropyl 2-(8-bromonaphthalen-1-yl)-2-(((R)-tert-butylsulfinyl)amino)-2-phenylacetate (**a**)-*P,P,P*

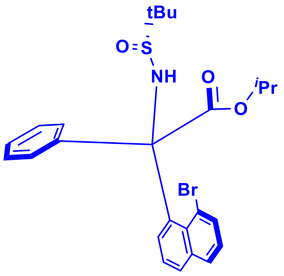

Isolation by column chromatography (petroleum ether/ ethyl acetate= 3/1 v/v), white solid, 350 mg, 70 % yield, mp 166.9-167.2 °C; ^{1}H NMR (400 MHz, $CDCl_3$) δ 8.33 (s, 1H), 7.90 - 7.85 (m, 3H), 7.64 - 7.29 (m, 5H), 7.14 (s, 1H), 6.93 (s, 1H), 5.70 (s, 1H), 5.10 - 5.03 (m, 1H), 1.30 (s, 9H), 1.00 (s, 6H). ^{13}C NMR (100 MHz, $CDCl_3$) δ 171.3, 144.7, 137.5, 136.8, 134.7, 134.0, 132.2, 131.3, 129.9, 128.3, 127.7, 127.2, 125.4, 124.6, 119.3, 73.8, 70.0, 57.3, 23.3, 20.8. HRMS (ESI) m/z: $[M+Na]^+$ Calcd for $C_{25}H_{28}BrNNaO_3S$ 524.0871; Found 524.0857. $[\alpha]_D^{25}$ = -30.600 (c = 1.0, CH_2Cl_2).

isopropyl 2-(8-bromonaphthalen-1-yl)-2-(((S)-tert-butylsulfinyl)amino)-2-phenylacetate (**a**)-*M,M,M*

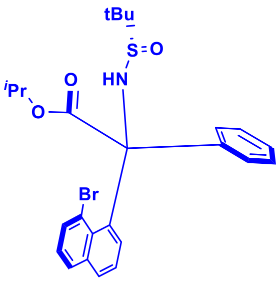

Isolation by column chromatography (petroleum ether/ ethyl acetate= 3/1 v/v), white solid, 384 mg, 77 % yield, mp 162.4-163.6 ºC; ^{1}H NMR (400 MHz, $CDCl_3$) δ 8.33 (s, 1H), 7.91 - 7.83 (m, 3H), 7.64 - 7.32 (m, 3H), 7.29 - 6.93 (m, 4H), 5.70 (s, 1H), 5.12 - 5.03 (m, 1H), 1.30 (s, 9H), 1.01 (s, 6H). ^{13}C NMR (100 MHz, $CDCl_3$) δ 171.3, 144.7, 136.8, 134.0, 131.3, 129.8, 127.7, 125.3, 124.6, 119.3, 73.8, 70.0, 57.3, 23.3, 20.8. HRMS (ESI) m/z: $[M+Na]^+$ Calcd for $C_{25}H_{28}BrNNaO_3S$ 524.0871; Found 524.0857. $[\alpha]_D^{25}$ = 18.826 (c = 1.25, CH_2Cl_2).

(R)-N-((8-methoxynaphthalen-1-yl)di-p-tolylmethyl)-2-methylpropane-2-sulfinamide (**b**)-*P,P,P*

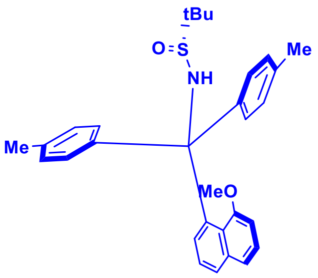

Isolation by column chromatography (petroleum ether/ ethyl acetate= 3/1 v/v), white solid, 220 mg, 47 % yield, mp 177.5-178.6 ºC; ^{1}H NMR (400 MHz, $CDCl_3$) δ 8.44 (s, 1H), 7.76 (d, J = 8.0 Hz, 1H), 7.53 (d, J = 7.9 Hz, 1H), 7.40 - 7.28 (m, 3H), 7.18 - 6.83 (m, 6H), 6.53 (d, J = 61.6 Hz, 2H), 3.27 (s, 3H), 2.32 (s, 6H). ^{13}C NMR (100 MHz, $CDCl_3$) δ 154.2, 135.2, 130.7, 129.0, 127.7, 125.3, 124.3, 123.2, 108.7, 65.3, 55.2, 23.2, 20.9. HRMS (ESI) m/z: $[M+Na]^+$ Calcd for $C_{30}H_{33}NNaO_2S$ 494.2130; Found 494.2134. $[\alpha]_D^{25}$ = 4.800 (c = 1.0, CH_2Cl_2).

(S)-2-methyl-N-(naphthalen-1-yldi-o-tolylmethyl)propane-2-sulfinamide (**c**)-*M,M,M*

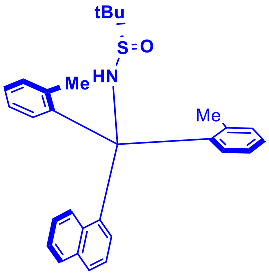

Isolation by column chromatography (petroleum ether/ ethyl acetate= 1/1 v/v), white solid, 42.3 mg, 60 % yield, mp 168.7-169.3 ºC; ^{1}H NMR (400 MHz, $CDCl_3$) δ 7.87 (d, J = 8.0 Hz, 3H), 7.45 - 7.32 (m, 5H), 7.28 (s, 1H), 7.21 - 7.06 (m, 5H), 6.91 (s, 1H), 5.57 (s, 1H), 2.08 (s, 3H), 1.62 (s, 3H), 1.23 (s, 9H). ^{13}C NMR (100 MHz, $CDCl_3$) δ 142.1, 137.7, 134.5, 132.9, 132.8, 130.8, 130.3, 129.0, 127.7, 127.5, 126.1, 125.0(4), 125.0(6), 124.6, 57.1, 23.9, 23.5. HRMS (ESI) m/z: $[M+Na]^+$ Calcd for $C_{29}H_{31}NNaOS$ 464.2024; Found 464.2023. $[\alpha]_D^{25}$ = 42.000 (c = 0.5, CH_2Cl_2).

(S)-2-methyl-N-(tri(naphthalen-1-yl)methyl)propane-2-sulfinamide (**d**)-*M,M,M*

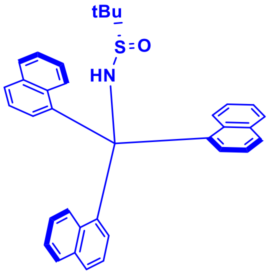

Isolation by column chromatography (petroleum ether/ ethyl acetate= 1/1 v/v), white solid, 45 mg, 55 % yield, mp 165.8-166.7 °C; ^{1}H NMR (400 MHz, $CDCl_3$) δ 8.10 - 7.55 (m, 13H), 7.27 (s, 3H), 7.00 (s, 5H), 6.17 (s, 1H), 1.14 (s, 9H). ^{13}C NMR (100 MHz, $CDCl_3$) δ 134.9, 133.7, 129.1, 126.8, 124.7, 57.4, 23.7. HRMS (ESI) m/z: $[M+Na]^+$ Calcd for $C_{35}H_{31}NNaOS$ 536.2024; Found 536.2036. $[\alpha]_D^{25}$ = 61.500 (c = 0.5, CH_2Cl_2).

isopropyl 2-(8-bromonaphthalen-1-yl)-2-(((R)-tert-butylsulfinyl)amino)-2-(p-tolyl)acetate (**e**)-*P,P,P*

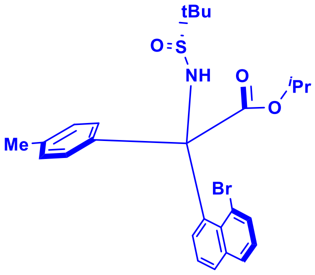

Isolation by column chromatography (petroleum ether/ ethyl acetate= 3/1 v/v), white solid, 276 mg, 63 % yield, mp 140.2-141.7 °C; ^{1}H NMR (400 MHz, $CDCl_3$) δ 8.18 (s, 1H), 7.86 - 7.80 (m, 3H), 7.56 (s, 1H), 7.32 (s, 1H), 7.23 (d, *J* = 7.6 Hz, 2H), 7.03 - 6.72 (m, 2H), 5.64 (s, 1H), 5.08 - 5.01 (m, 1H), 2.32 (s, 3H), 1.26 (s, 9H), 0.99 (s, 6H). ^{13}C NMR (100 MHz, $CDCl_3$) δ 171.4, 141.6, 137.5, 136.7, 134.9, 134.0, 132.2, 131.2, 129.8, 128.8, 125.3, 124.6, 119.3, 73.5, 69.9, 57.3, 23.3, 21.0, 20.9. HRMS (ESI) m/z: $[M+H]^+$ Calcd for $C_{26}H_{32}NO_3S$ 438.2103; Found 438.2099. $[\alpha]_D^{25}$ = -24.600 (c = 1.0, CH_2Cl_2).

isopropyl 2-(8-bromonaphthalen-1-yl)-2-(((R)-tert-butylsulfinyl)amino)-2-(4-isopropylphenyl)acetate (**f**)-*P,P,P*

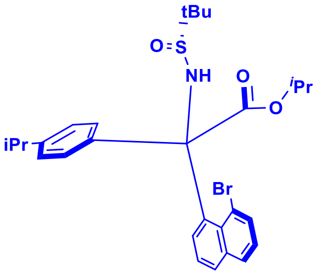

Isolation by column chromatography (petroleum ether/ ethyl acetate= 3/1 v/v), white solid, 305 mg, 56 % yield, mp 147.0-141.9 °C; ^{1}H NMR (400 MHz, $CDCl_3$) δ 8.20 (s, 1H), 7.86 - 7.81 (m, 4H), 7.58 (s, 1H), 7.33 (s, 1H), 7.22 (d, *J* = 7.6 Hz, 1H), 6.88 - 6.42 (m, 2H), 5.65 (s, 1H), 5.03 (dd, *J* = 12.4, 6.0 Hz, 1H), 2.90 - 2.85 (m, 1H), 1.26 (s, 9H), 1.23 (s, 3H), 1.21 (s, 3H), 0.97 (s, 6H). ^{13}C NMR (100 MHz, $CDCl_3$) δ 171.4, 148.4, 141.8, 137.4, 136.7, 134.9, 133.9, 132.2, 131.2, 129.8, 128.4, 125.3, 124.6, 119.3, 73.6, 69.9, 57.2, 33.6, 23.9, 23.8, 23.2, 20.9. HRMS (ESI) m/z: $[M+H]^+$ Calcd for $C_{28}H_{35}BrNO_3S$ 544.1521; Found 544.1492. $[\alpha]_D^{25}$ = -69.607 (c = 3.65, CH_2Cl_2).

isopropyl 2-(8-bromonaphthalen-1-yl)-2-(((R)-tert-butylsulfinyl)amino)-2-(4-methoxyphenyl)acetate (**g**)-

P,P,P

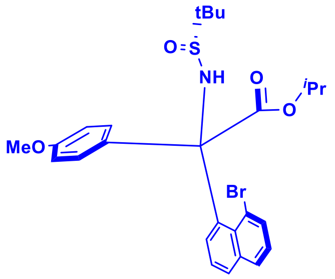

Isolation by column chromatography (petroleum ether/ ethyl acetate= 3/1 v/v), white solid,422 mg, 80 % yield, mp 155.8-157.3 ºC; ^{1}H NMR (400 MHz, $CDCl_3$) δ 8.22 (s, 1H), 7.84 - 7.81 (m, 3H), 7.60 (s, 1H), 7.32 (d, *J* = 7.2 Hz, 1H), 7.24 (d, *J* = 7.6 Hz, 1H), 6.96 - 6.65 (m, 3H), 5.63 (s, 1H), 5.05 - 5.00 (m, 1H), 3.79 (s, 3H), 1.25 (s, 9H), 0.98 (s, 6H). ^{13}C NMR (100 MHz, $CDCl_3$) δ 171.3, 159.1, 136.8, 136.4, 134.0, 132.1, 131.2, 129.8, 125.3, 124.6, 119.2, 73.3, 69.9, 57.2, 55.2, 23.2, 20.9. HRMS (ESI) m/z: $[M+Na]^+$ Calcd for $C_{26}H_{30}BrNNaO_4S$ 554.0977; Found 554.0960. $[\alpha]_D^{25}$ = -8.889 (c = 0.65, CH_2Cl_2).

isopropyl 2-(8-bromonaphthalen-1-yl)-2-(((R)-tert-butylsulfinyl)amino)-2-(4-propoxyphenyl)acetate **(h)**-*P,P,P*

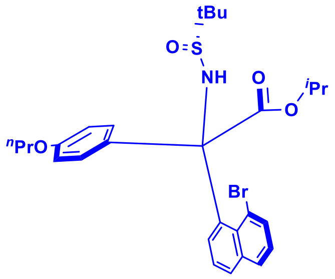

Isolation by column chromatography (petroleum ether/ ethyl acetate= 3/1 v/v), white solid, 322 mg, 58 % yield, mp 145.2-146.8 ºC; ^{1}H NMR (400 MHz, $CDCl_3$) δ 8.20 (s, 1H), 7.85 - 7.81 (m, 3H), 7.61 (s, 1H), 7.33 (s, 1H), 7.23 (d, *J* = 7.6 Hz, 1H), 6.96 - 6.63 (m, 3H), 5.62 (s, 1H), 5.06 - 5.00 (m, 1H), 3.91 (t, *J* = 6.8 Hz, 2H), 1.79 (dd, *J* = 14.0, 6.8 Hz, 2H), 1.25 (s, 9H), 1.04 (t, *J* = 7.2 Hz, 3H). ^{13}C NMR (100 MHz, $CDCl_3$) δ 171.3, 158.7, 136.8, 136.1, 133.9, 131.1, 129.8, 125.2, 124.6, 119.3, 100.0, 73.3, 69.9, 69.5, 57.3, 23.2, 22.6, 20.9, 10.5. HRMS (ESI) m/z: $[M+Na]^+$ Calcd for $C_{28}H_{34}BrNNaO_4S$ 582.1290; Found 582.1277. $[\alpha]_D^{25}$ = -6.630 (c = 0.6, CH_2Cl_2).

isopropyl 2-(8-bromonaphthalen-1-yl)-2-(4-bromophenyl)-2-(((R)-tert-butylsulfinyl)amino)acetate **(i)**-*P,P,P*

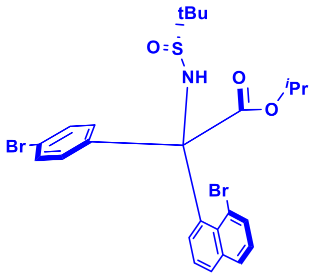

Isolation by column chromatography (petroleum ether/ ethyl acetate= 3/1 v/v), white solid, 334 mg, 58 % yield, mp 181.1-182.5 ºC; ^{1}H NMR (400 MHz, $CDCl_3$) δ 8.21 (s, 1H), 7.84 (dd, *J* = 7.6, 3.2 Hz, 3H), 7.61 - 7.46 (m, 2H), 7.33 (s, 1H), 7.26 (d, *J* = 3.6 Hz, 1H), 7.24 (d, *J* = 7.6 Hz, 1H), 6.79 (s, 1H), 5.63 (s, 1H), 5.05 (dd, *J* = 12.4, 6.4 Hz, 1H), 1.26 (s, 9H), 0.99 (s, 6H). ^{13}C NMR (100 MHz, $CDCl_3$) δ 170.9, 143.9, 136.8, 134.1, 131.5, 129.9, 125.5, 124.6, 122.1, 119.1, 73.6, 70.3, 57.4, 23.4, 20.9. HRMS (ESI) m/z: $[M+Na]^+$ Calcd

for $C_{25}H_{27}Br_2NNaO_3S$ 601.9976 ; Found 601.9978. $[\alpha]_D^{25}$ = -60.460 (c = 1.5, CH_2Cl_2).

isopropyl 2-(8-bromonaphthalen-1-yl)-2-(((R)-tert-butylsulfinyl)amino)-2-(4-chlorophenyl)acetate **(j)**-*P,P,P*

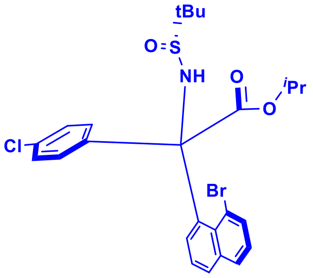

Isolation by column chromatography (petroleum ether/ ethyl acetate= 3/1 v/v), white solid, 432 mg, 81 % yield, mp 148.4-149.3 °C; ^{1}H NMR (400 MHz, $CDCl_3$) δ 8.28 (s, 1H), 7.84 (dd, *J* = 8.0, 3.6 Hz, 3H), 7.58 - 7.32 (m, 3H), 7.24 (d, *J* = 7.6 Hz, 1H), 7.09 (s, 1H), 6.86 (s, 1H), 5.64 (s, 1H), 5.07 - 5.01 (m, 1H), 1.26 (s, 9H), 0.99 (s, 6H). ^{13}C NMR (100 MHz, $CDCl_3$) δ 170.9, 143.4, 136.8, 134.1, 133.8, 131.4, 129.9, 128.1, 125.5, 124.6, 119.2, 73.5, 70.3, 57.4, 23.2, 20.8. HRMS (ESI) m/z: $[M+Na]^+$ Calcd for $C_{28}H_{34}BrNNaO_4S$ 582.1290; Found 582.1295. $[\alpha]_D^{25}$ = -6.939 (c = 0.58, CH_2Cl_2).

isopropyl 2-(8-bromonaphthalen-1-yl)-2-(((R)-tert-butylsulfinyl)amino)-2-(4-fluorophenyl)acetate **(k)**-*P,P,P*

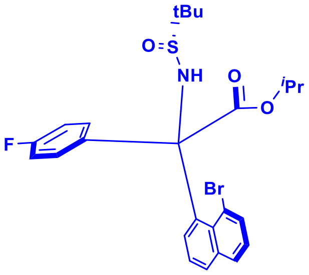

Isolation by column chromatography (petroleum ether/ ethyl acetate= 3/1 v/v), white solid, 330 mg, 64 % yield, mp 140.7-141.3 °C; ^{1}H NMR (400 MHz, $CDCl_3$) δ 8.31 (s, 1H), 7.86 - 7.82 (m, 3H), 7.54 (s, 1H), 7.33 (s, 1H), 7.23 (d, *J* = 7.6 Hz, 1H), 7.14 - 6.79 (m, 3H), 5.65 (s, 1H), 5.03 (dd, *J* = 12.4, 6.4 Hz, 1H), 1.26 (s, 9H), 0.98 (s, 6H). ^{13}C NMR (100 MHz, $CDCl_3$) δ 171.1, 163.5, 161.1, 140.3, 136.8, 134.7, 134.1, 131.4, 129.9, 125.4, 124.6, 119.1, 114.6, 114.4, 73.4, 70.2, 57.3, 23.2, 20.8. HRMS (ESI) m/z: $[M+Na]^+$ Calcd for $C_{25}H_{27}BrFNNaO_3S$ 542.0777; Found 542.0761. $[\alpha]_D^{25}$ = -35.800 (c = 1.0, CH_2Cl_2).

isopropyl 2-(8-bromonaphthalen-1-yl)-2-(((R)-tert-butylsulfinyl)amino)-2-(3-fluorophenyl)acetate **(l)**-*P,P,P*

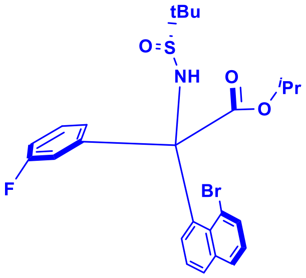

Isolation by column chromatography (petroleum ether/ ethyl acetate= 3/1 v/v), white solid, 288 mg, 56 % yield, mp 170.1-171.2 °C; ^{1}H NMR (400 MHz, $CDCl_3$) δ 8.15 (s, 1H), 7.88 (d, J = 7.6 Hz, 3H), 7.58 – 7.30 (m, 3H), 7.21 – 6.93 (m, 2H), 6.70 (d, J = 29.6 Hz, 1H), 5.67 (s, 1H), 5.11 – 5.05 (m, 1H), 1.31 (s, 9H), 1.03 (s, 6H). ^{13}C NMR (100 MHz, $CDCl_3$) δ 170.9, 147.5, 136.8, 134.1, 132.1, 131.5, 129.9, 125.5, 124.7, 119.2, 116.4, 73.7, 70.3, 57.4, 23.2, 20.8. HRMS (ESI) m/z: $[M+Na]^+$ Calcd for $C_{25}H_{27}BrFNNaO_3S$ 542.0777; Found 542.0784. $[\alpha]_D^{25}$ = -34.500 (c = 1.0, CH_2Cl_2).

isopropyl 2-(8-bromonaphthalen-1-yl)-2-(((R)-tert-butylsulfinyl)amino)-2-(2-fluorophenyl)acetate (**m**)-*P,P,P*

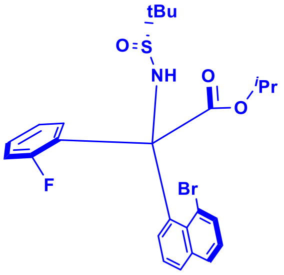

Isolation by column chromatography (petroleum ether/ ethyl acetate= 3/1 v/v), white solid, 311 mg, 60 % yield; mp 157.3-157.9 °C ^{1}H NMR (400 MHz, $CDCl_3$) δ 8.13 (s, 1H), 7.85 (d, J = 8.0 Hz, 4H), 7.55 - 7.31 (m, 3H), 7.24 (d, J = 8.0 Hz, 1H), 6.97 (d, J = 6.8 Hz, 1H), 5.65 (s, 1H), 5.05 (dd, J = 12.4, 6.4 Hz, 1H), 1.28 (s, 9H), 1.00 (s, 6H). ^{13}C NMR (100 MHz, $CDCl_3$) δ 170.9, 136.8, 134.1, 131.5, 129.9, 125.5, 124.7, 119.2, 116.4, 73.7, 70.3, 57.4, 23.2, 20.8, 20.8. HRMS (ESI) m/z: $[M+Na]^+$ Calcd for $C_{25}H_{27}BrFNNaO_3S$ 542.0777; Found 542.0764. $[\alpha]_D^{25}$ = -29.200 (c = 1.0, CH_2Cl_2).

isopropyl 2-(8-bromonaphthalen-1-yl)-2-(((R)-tert-butylsulfinyl)amino)-2-(4-(trifluoromethyl)phenyl)acetate (**n**)-*P,P,P*

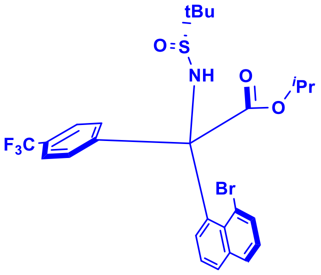

Isolation by column chromatography (petroleum ether/ ethyl acetate= 3/1 v/v), white solid, 380 mg, 67 % yield, mp 168.3-169.5 °C; ^{1}H NMR (400 MHz, $CDCl_3$) δ 8.52 (s, 1H), 7.88 (d, J = 8.0 Hz, 3H), 7.75 (s, 1H), 7.90 - 7.86 (m, 3H), 7.50 - 7.34 (m, 1H), 7.08 (s, 1H), 5.74 (s, 1H), 5.11 - 5.05 (m, 1H), 1.31 (s, 9H), 1.01 (s, 6H). ^{13}C NMR (100 MHz, $CDCl_3$) δ 170.8, 149.0, 136.9, 134.2, 132.0, 131.6, 130.1, 129.9, 129.8, 129.4, 125.6, 125.4, 124.6, 122.7, 119.1, 73.9, 70.5, 57.4, 23.2, 20.8. HRMS (ESI) m/z: $[M+H]^+$ Calcd for $C_{26}H_{28}BrF_3NO_3S$ 570.0925; Found 570.0920. $[\alpha]_D^{25}$ = -23.567 (c = 1.18, CH_2Cl_2).

isopropyl 2-(((R)-tert-butylsulfinyl)amino)-2-(8-methylnaphthalen-1-yl)-2-phenylacetate (**o**)-*P,P,P*

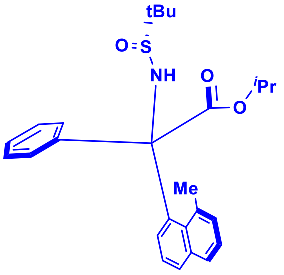

Isolation by column chromatography (petroleum ether/ ethyl acetate= 3/1 v/v), white solid, 310 mg, 71 % yield, mp 186.7-188.1 °C; 1H NMR (400 MHz, $CDCl_3$) δ 7.82 (d, *J* = 8.0 Hz, 1H), 7.73 (dd, *J* = 7.2, 2.0 Hz, 1H), 7.53 - 7.31 (m, 5H), 7.31 - 7.26 (m, 2H), 7.24 (d, *J* = 7.6 Hz, 1H), 5.65 (s, 1H), 5.02 - 4.96 (m, 1H), 2.78 (s, 3H), 1.28 (s, 9H), 1.00 - 0.89 (m, 6H). ^{13}C NMR (100 MHz, $CDCl_3$) δ 144.1, 135.7, 134.2, 133.5, 131.5, 130.5, 128.0, 127.8, 124.6, 123.5, 74.6, 70.0, 56.8, 24.2, 23.2, 20.8. HRMS (ESI) m/z: $[M+H]^+$ Calcd for $C_{26}H_{32}NO_3S$ 438.2103; Found 438.2099. $[\alpha]_D^{25}$ = -39.200 (c = 1.0, CH_2Cl_2).

isopropyl 2-(((R)-tert-butylsulfinyl)amino)-2-(8-methoxynaphthalen-1-yl)-2-phenylacetate (**p**)-*P,P,P*

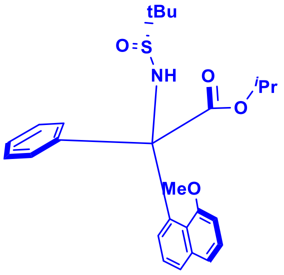

Isolation by column chromatography (petroleum ether/ ethyl acetate= 3/1 v/v), white solid, 485 mg, 85 % yield, mp 170.5-171.8 °C; ^1H NMR (400 MHz, $CDCl_3$) δ 8.37 (s, 1H), 7.80 - 7.76 (m, 1H), 7.48 (d, *J* = 8.0 Hz, 2H), 7.42 - 7.35 (m, 2H), 7.27 (d, *J* = 6.0 Hz, 3H), 7.20 (s, 1H), 6.81 (d, *J* = 4.4 Hz, 1H), 5.56 (s, 1H), 5.00 - 4.93 (m, 1H), 3.77 (s, 3H), 1.09 (s, 9H), 0.95 (d, *J* = 21.6 Hz, 6H). ^{13}C NMR (100 MHz, $CDCl_3$) δ 170.8, 155.0, 142.6, 136.3, 132.8, 129.7, 128.5, 127.7, 126.8, 125.4, 124.9, 124.6, 122.0, 105.2, 74.5, 68.9, 56.2, 52.9, 22.7, 21.3, 21.0. HRMS (ESI) m/z: $[M+H]^+$ Calcd for $C_{39}H_{44}NOS$ 574.3144; Found 574.3116. $[\alpha]_D^{25}$ = -28.350 (c = 1.10, CH_2Cl_2).

isopropyl 2-(8-acetylnaphthalen-1-yl)-2-(((R)-tert-butylsulfinyl)amino)-2-phenylacetate (**q**)-*P,P,P*

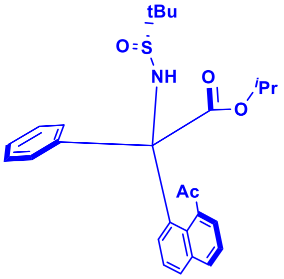

Isolation by column chromatography (petroleum ether/ ethyl acetate= 3/1 v/v), white solid, 301 mg, 65 % yield, mp 163.5-164.9 °C; ^1H NMR (400 MHz, $CDCl_3$) δ 8.57 (d, *J* = 8.0 Hz, 1H), 8.05 - 8.01 (m, 2H), 7.92 - 7.89 (m, 1H), 7.65 - 7.61 (m, 2H), 7.58 - 7.52 (m, 3H), 7.44 - 7.40 (m, 2H), 7.38 - 7.35 (m, 1H), 5.35 (s, 1H), 5.22 - 5.15 (m, 1H), 4.32 (s, 2H), 1.25 (s, 9H), 1.22 (dd, *J* = 6.4, 4.0 Hz, 6H). ^{13}C NMR (100 MHz, $CDCl_3$) δ 199.9, 171.4, 140.0, 135.2, 134.0, 133.1, 130.1, 128.7, 128.5, 128.4, 128.0, 127.8, 126.5, 126.4, 125.6, 124.4, 70.0, 65.6, 56.9, 48.6, 22.9, 22.8, 21.5, 21.5. HRMS (ESI) m/z: $[M+H]^+$ Calcd for $C_{27}H_{32}NO_4S$ 466.2052; Found 466.2059. $[\alpha]_D^{25}$ = -26.500 (c = 1.0, CH_2Cl_2)

isopropyl 2-(8-bromo-2,7-dimethoxynaphthalen-1-yl)-2-(((R)-tert-butylsulfinyl)amino)-2-phenylacetate* (r)-*P,P,P

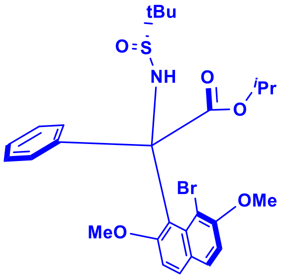

Isolation by column chromatography (petroleum ether/ ethyl acetate= 3/1 v/v), white solid, 317 mg, 66 % yield, mp 195.8-196.1 °C; ^{1}H NMR (400 MHz, $CDCl_3$) δ 7.83 (d, J = 8.8 Hz, 1H), 7.71 (d, J = 7.2 Hz, 2H), 7.60 (d, J = 8.8 Hz, 1H), 7.32 - 7.27 (m, 2H), 7.10 (d, J = 8.8 Hz, 1H), 6.85 (dd, J = 8.8, 2.4 Hz, 1H), 6.55 (d, J = 2.0 Hz, 1H), 5.88 (s, 1H), 4.96 - 4.88 (m, 1H), 3.88 (s, 3H), 3.20 (s, 3H), 1.19 (d, J = 5.6 Hz, 12H), 0.90 (d, J = 6.4 Hz, 3H). ^{13}C NMR (100 MHz, $CDCl_3$) δ 172.8, 156.6, 156.1, 142.5, 134.8, 131.1, 129.3, 128.9, 127.8, 127.4, 125.0, 118.6, 116.5, 109.3, 106.3, 69.8, 69.7, 56.4, 55.1, 54.7, 22.6, 21.6, 20.8. HRMS (ESI) m/z: [M+H] $^{+}$ Calcd for $C_{27}H_{34}NO_5S$ 484.2158; Found 484.2154. $[\alpha]_D^{25}$ = -32.660 (c = 1.20, CH_2Cl_2).

(R)-N-(furan-3-yl(8-methoxynaphthalen-1-yl)(phenyl)methyl)-2-methylpropane-2-sulfinamide* (s)-*P,P,P

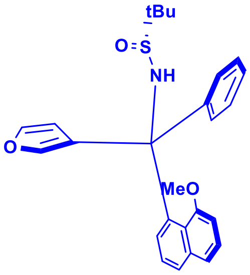

Isolation by column chromatography (petroleum ether/ ethyl acetate= 5/1 - 3/1 v/v), white solid, 250 mg, 58 % yield, mp 123.6-124.9 °C; ^{1}H NMR (400 MHz, $CDCl_3$) δ 7.79 (d, J = 8.0 Hz, 1H), 7.55 (d, J = 8.0 Hz, 2H), 7.44 (s, 1H), 7.38 - 7.33 (m, 2H), 7.17 (d, J = 7.2 Hz, 1H), 6.82 (d, J = 7.2 Hz, 2H), 6.50 (s, 1H), 6.26 (s, 1H), 3.23 (s, 3H), 1.06 (s, 9H). ^{13}C NMR (100 MHz, $CDCl_3$) δ 153.9, 147.5, 144.7, 142.3, 140.2, 137.3, 129.2, 129.0, 127.1, 126.0, 125.5, 124.7, 123.8, 123.3, 114.2, 108.5, 70.8, 56.3, 54.8, 23.0. HRMS (ESI) m/z: [M+Na] $^{+}$ Calcd for $C_{26}H_{27}NNaO_3S$ 456.1609; Found 456.1618. $[\alpha]_D^{25}$ = -23.800 (c = 1.0, CH_2Cl_2).

(R)-N-((8-methoxynaphthalen-1-yl)(phenyl)(thiophen-2-yl)methyl)-2-methylpropane-2-sulfinamide* (t)-*P,P,P

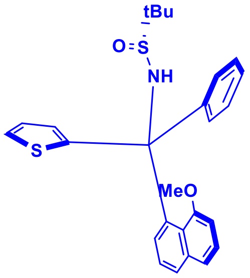

Isolation by column chromatography (petroleum ether/ ethyl acetate= 5/1 -3/1 v/v), white solid, 326 mg, 73 % yield, mp 147.7-149.2 °C; ^{1}H NMR (400 MHz, $CDCl_3$) δ 7.79 (d, J = 8.0 Hz, 1H), 7.54 (d, J = 8.0 Hz, 1H), 7.51 - 7.26 (m, 6H), 7.25 - 7.16 (m, 2H), 6.94 (s, 1H), 6.89 - 6.78 (m, 2H), 6.72 (s, 1H), 3.25 (s, 3H), 1.14 (s, 9H). ^{13}C NMR (100 MHz, $CDCl_3$) δ 154.0, 137.0, 129.9, 129.5, 127.8, 127.6, 126.4, 125.8, 125.5, 124.6,

123.2, 108.4, 56.9, 54.7, 23.2.HRMS (ESI) m/z: [M+Na] $^{+}$ Calcd for $C_{26}H_{27}NNaO_2S_2$ 472.1381; Found 472.1371. $[\alpha]_D^{25}$ = -66.630 (c = 2.1, CH_2Cl_2).

4. NMR Spectra of Compounds (a)-*P*,*P*,*P* - (t)-*P*,*P*,*P*

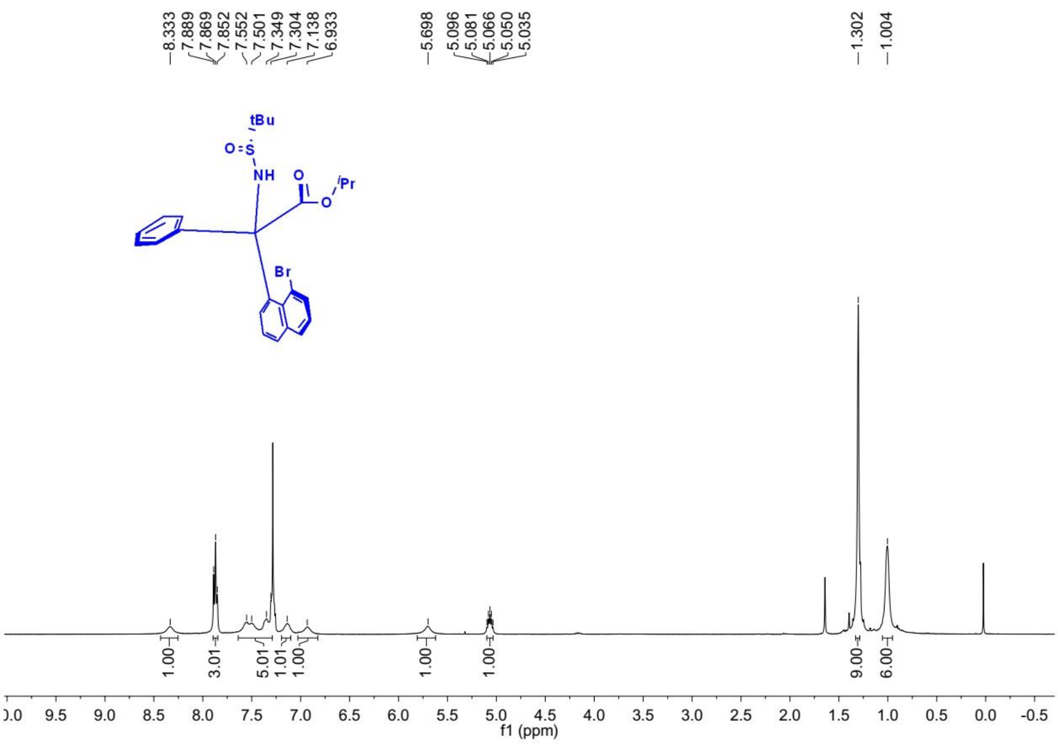

Figure 1. ^{1}H NMR Spectrum of Compound (a)-*P*,*P*,*P* ($CDCl_3$, 400 MHz)

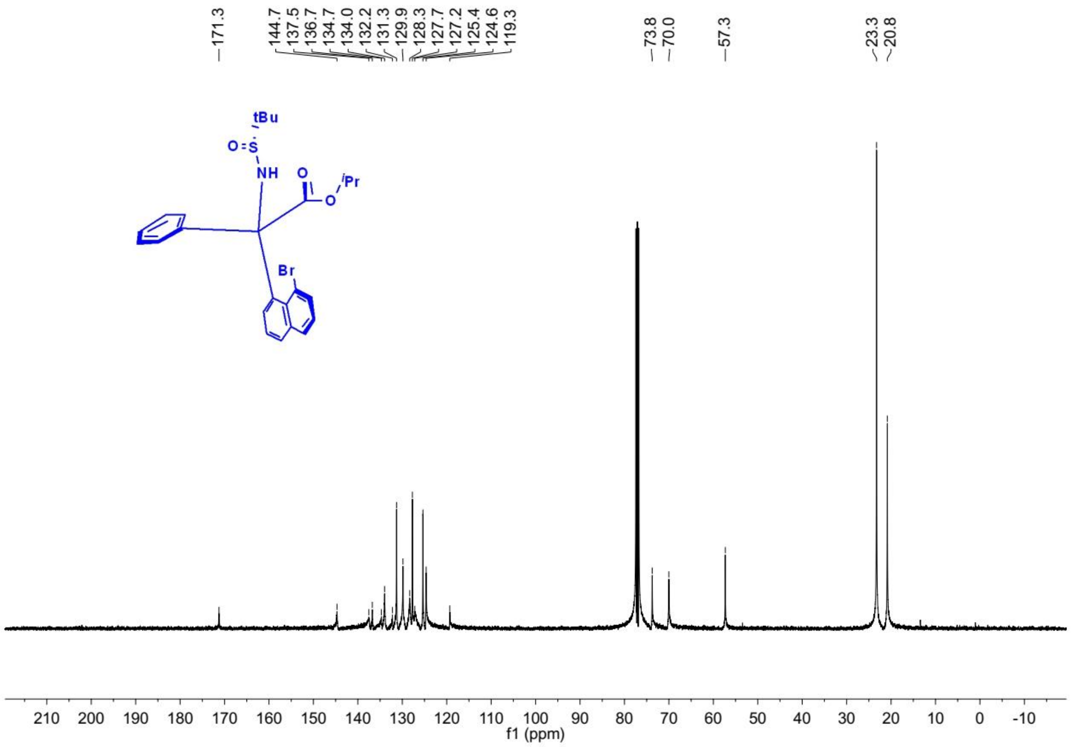

Figure 2. ^{13}C NMR Spectrum of Compound (a)-*P*,*P*,*P* ($CDCl_3$, 100 MHz)

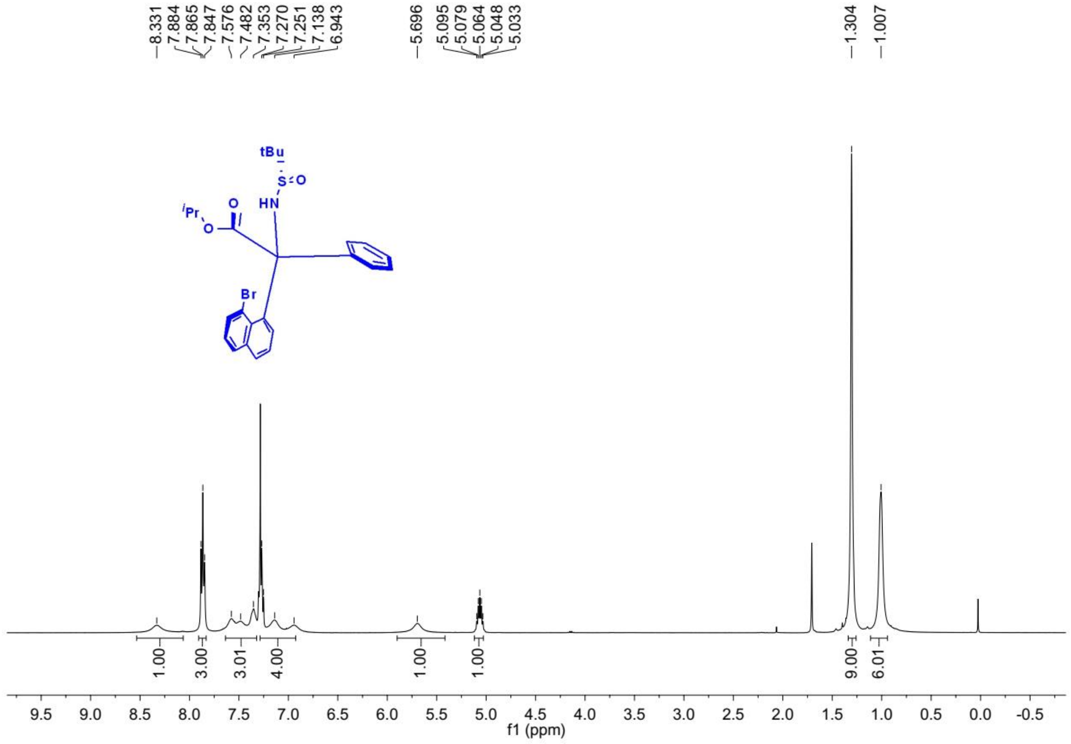

Figure 3. ^{1}H NMR Spectrum of Compound (a)-*M*,*M*,*M* ($CDCl_3$, 400 MHz)

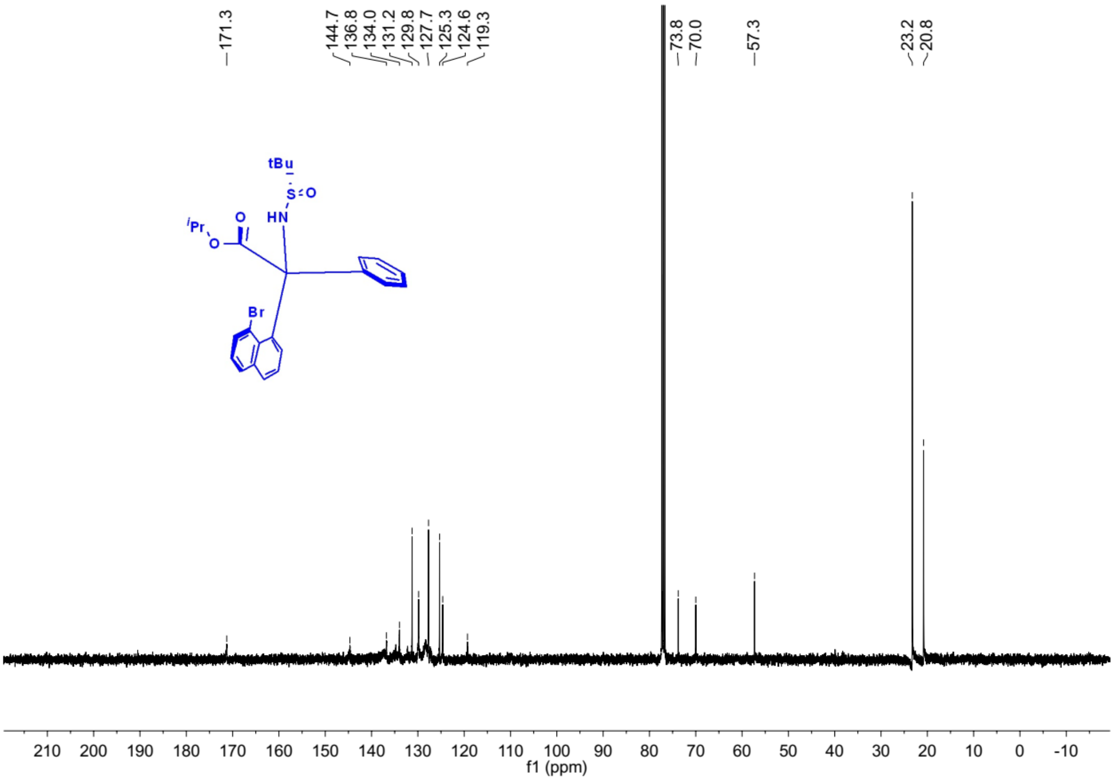

Figure 4. ^{13}C NMR Spectrum of Compound (a)-*M*,*M*,*M* ($CDCl_3$, 100 MHz)

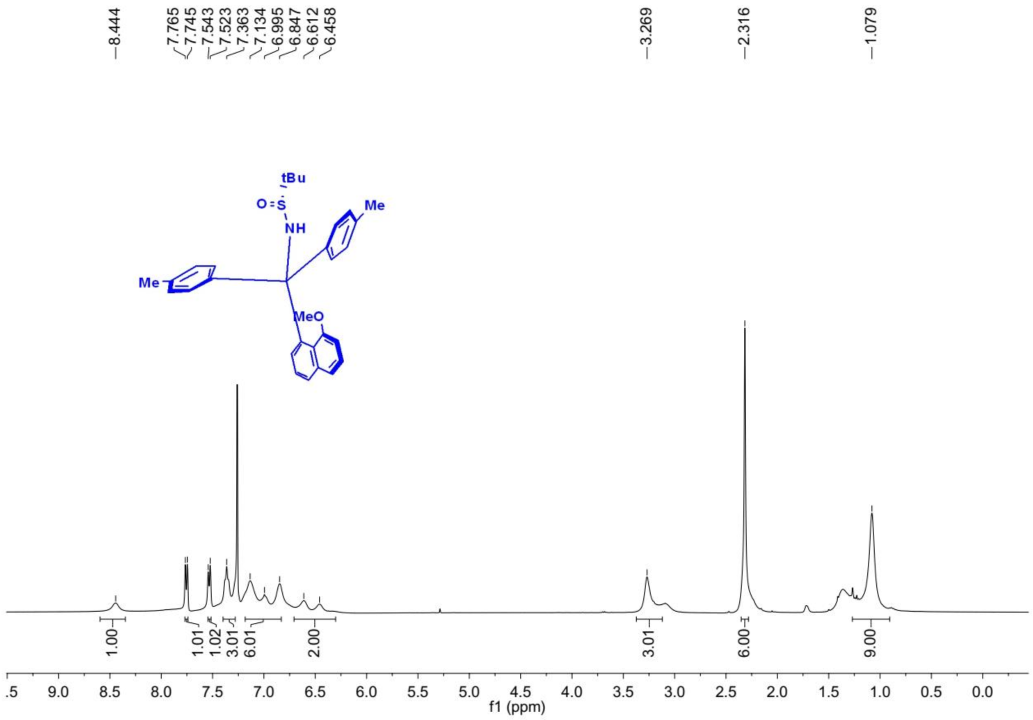

Figure 5. ¹H NMR Spectrum of Compound (**b**)-*P*,*P*,*P* (CDCl3, 400 MHz)

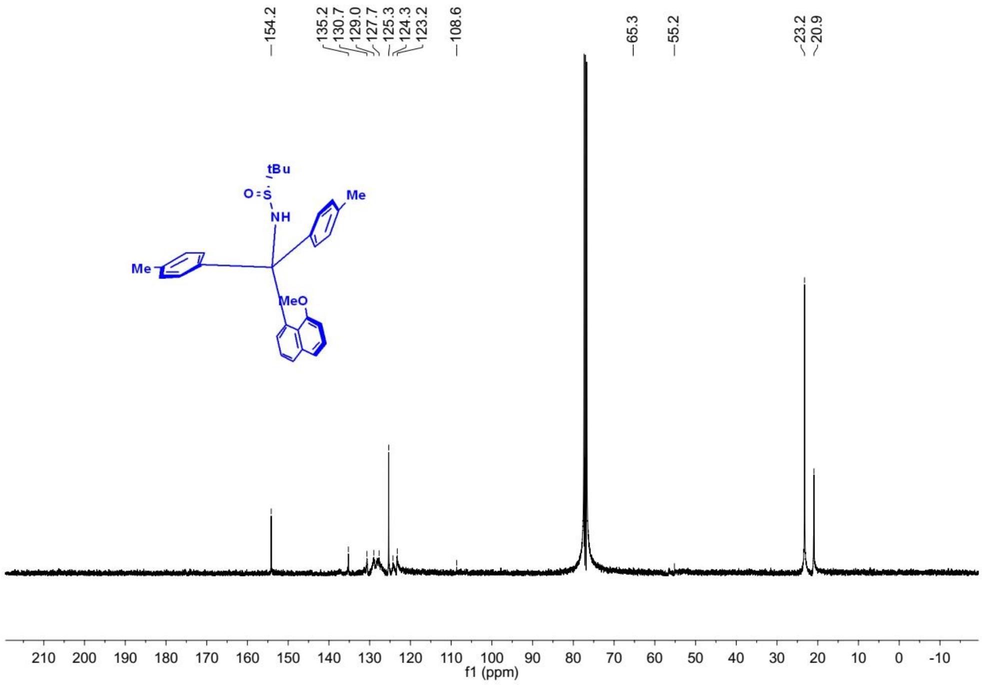

Figure 6. ¹³C NMR Spectrum of Compound (**b**)-*P*,*P*,*P* (CDCl3, 100 MHz)

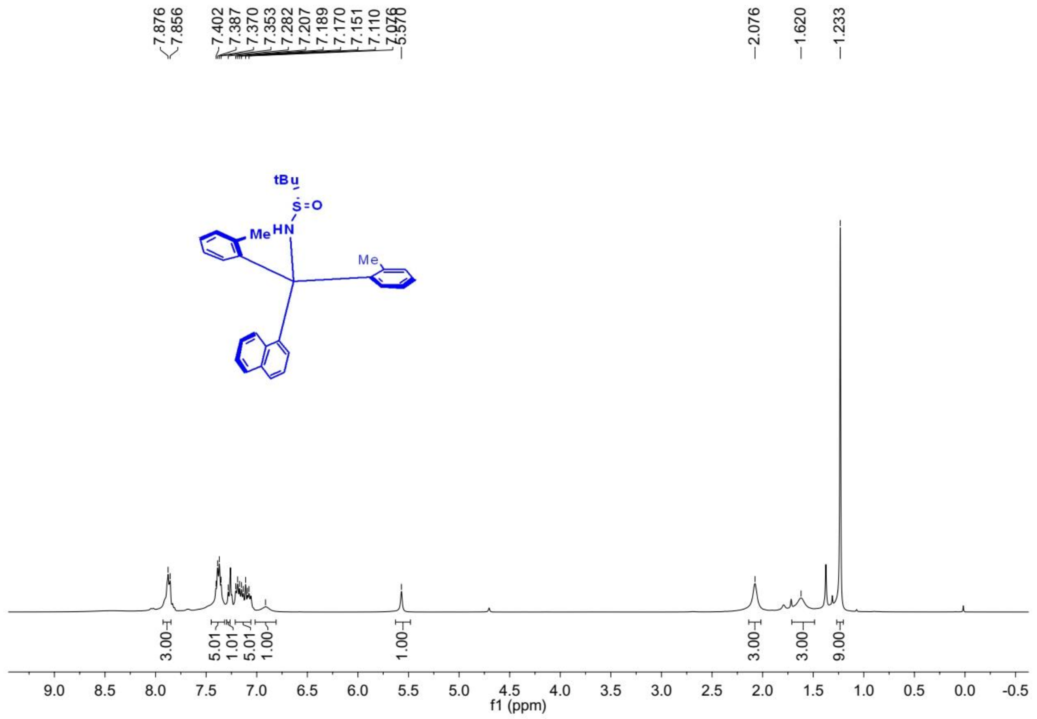

Figure 7. ^{1}H NMR Spectrum of Compound (c)-*M*,*M*,*M* (CDCl$_3$, 400 MHz)

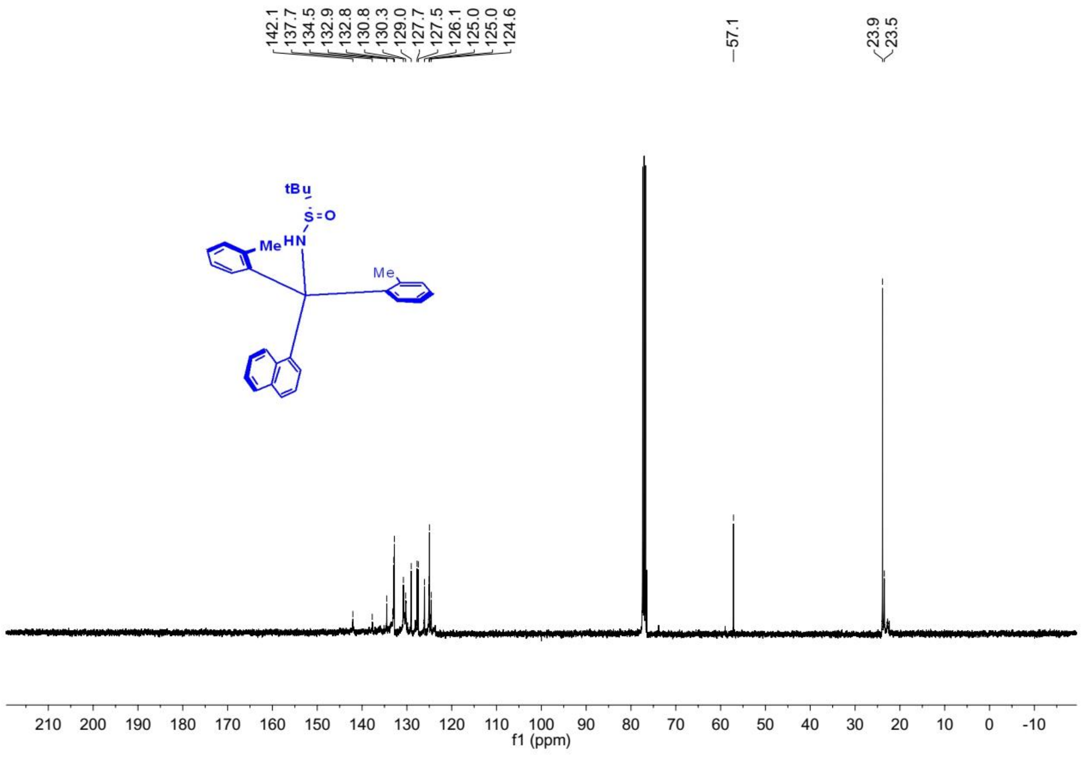

Figure 8. ^{13}C NMR Spectrum of Compound (c)-*M*,*M*,*M* (CDCl$_3$, 100 MHz)

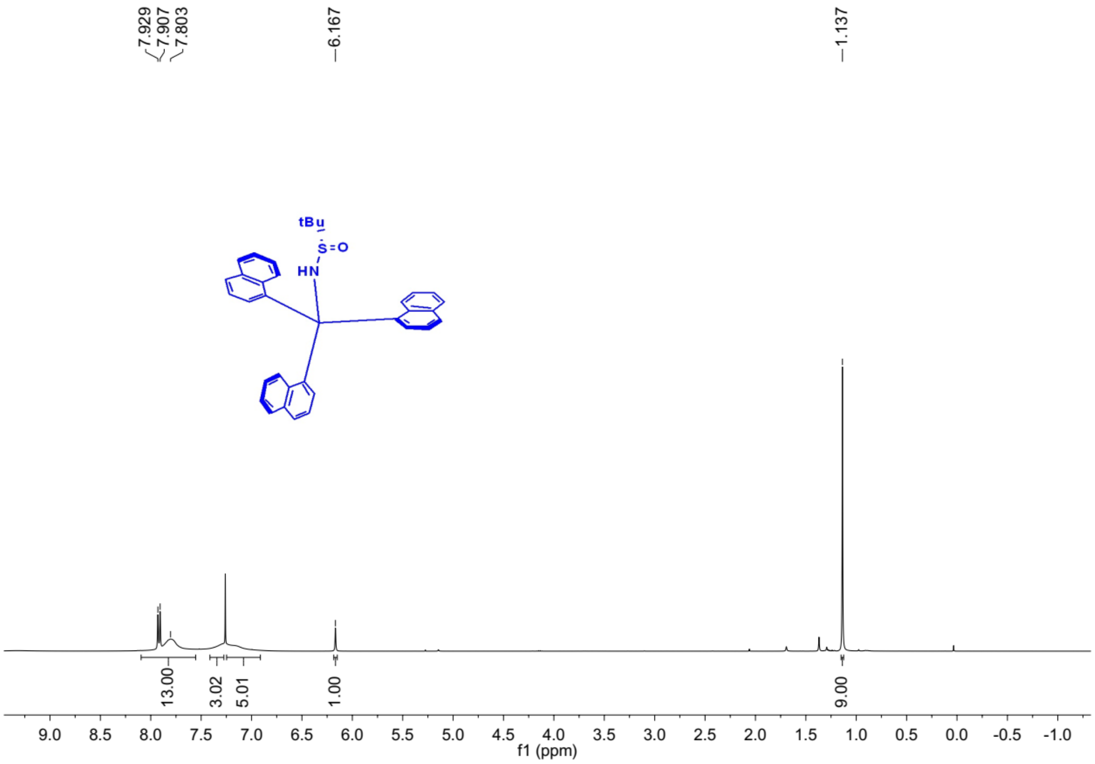

Figure 9. ^{1}H NMR Spectrum of Compound (d)-*M,M,M* ($CDCl_3$, 400 MHz)

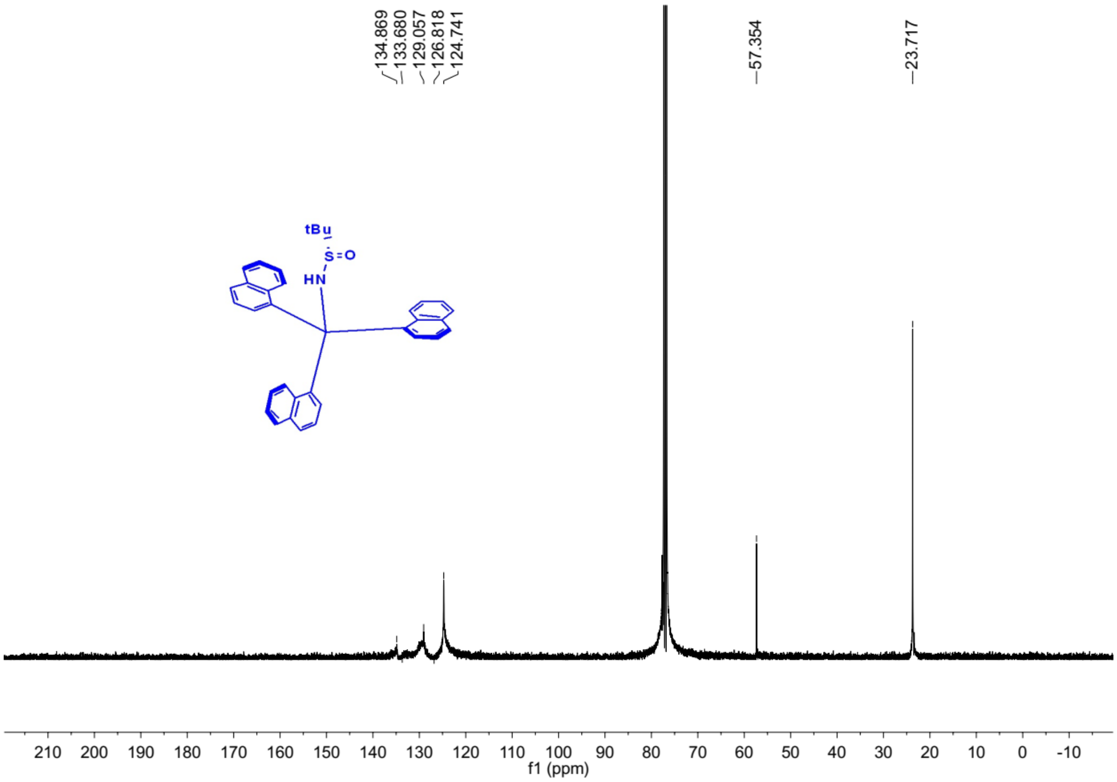

Figure 10. ^{13}C NMR Spectrum of Compound (d)-*M,M,M* ($CDCl_3$, 100 MHz)

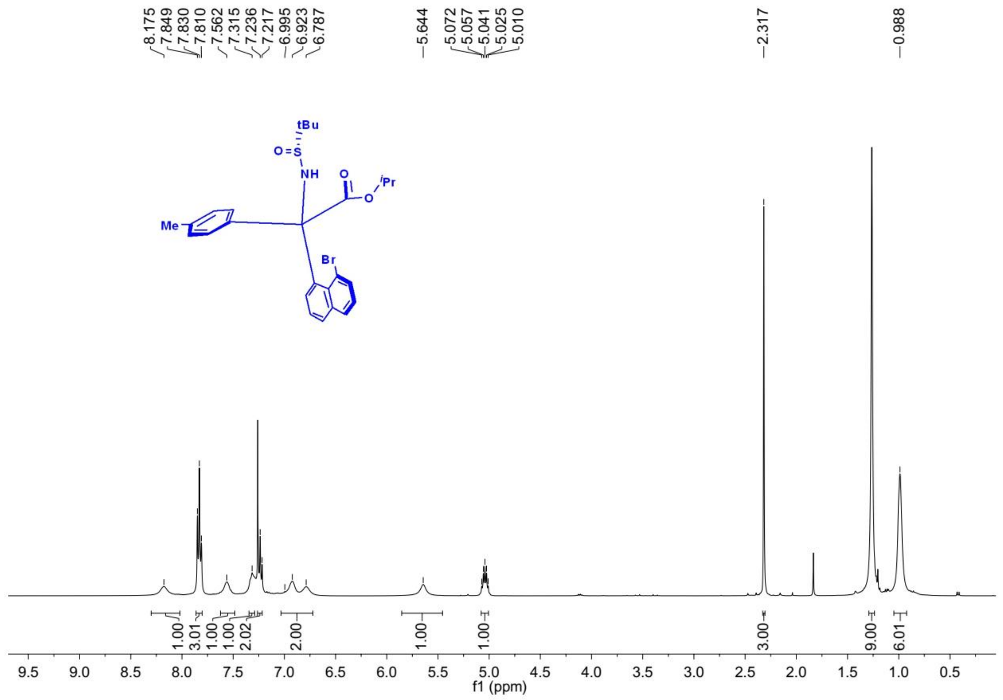

Figure 11. **^{1}H NMR Spectrum of Compound** (**e**)-*P*,*P*,*P* **(CDCl3, 400 MHz)**

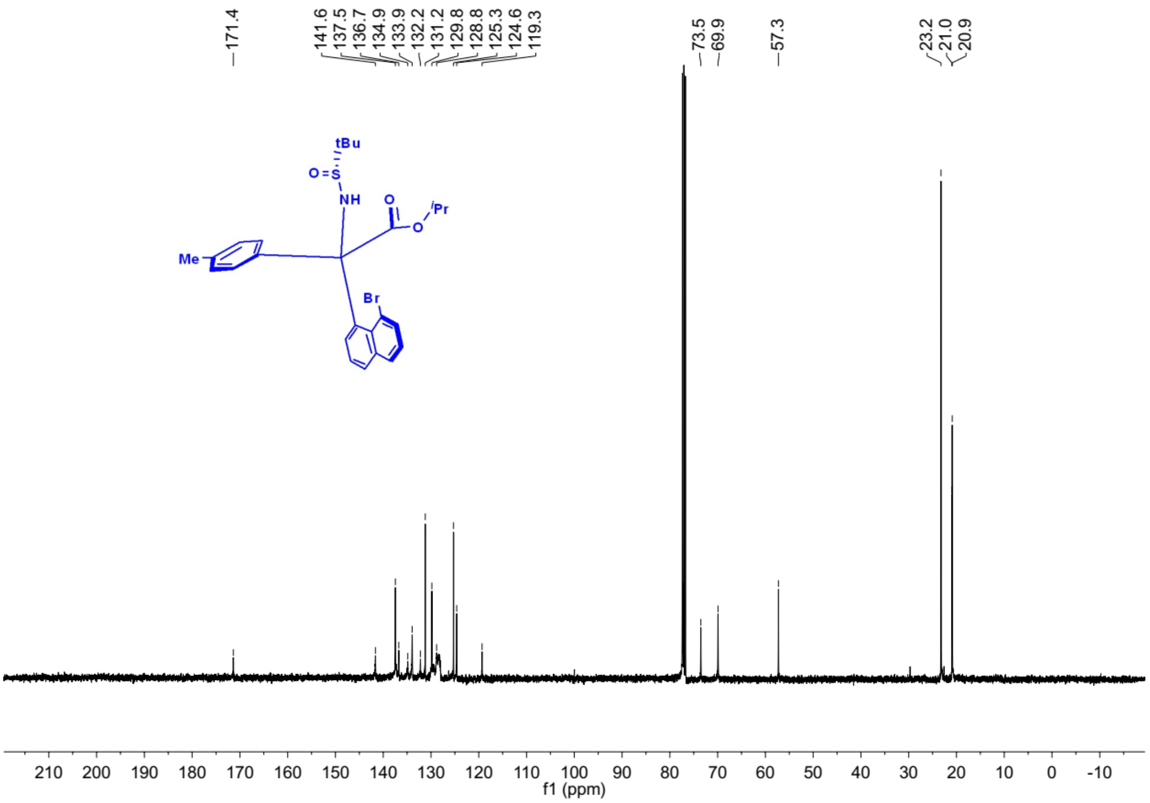

Figure 12. **^{13}C NMR Spectrum of Compound** (**e**)-*P*,*P*,*P* **(CDCl3, 100 MHz)**

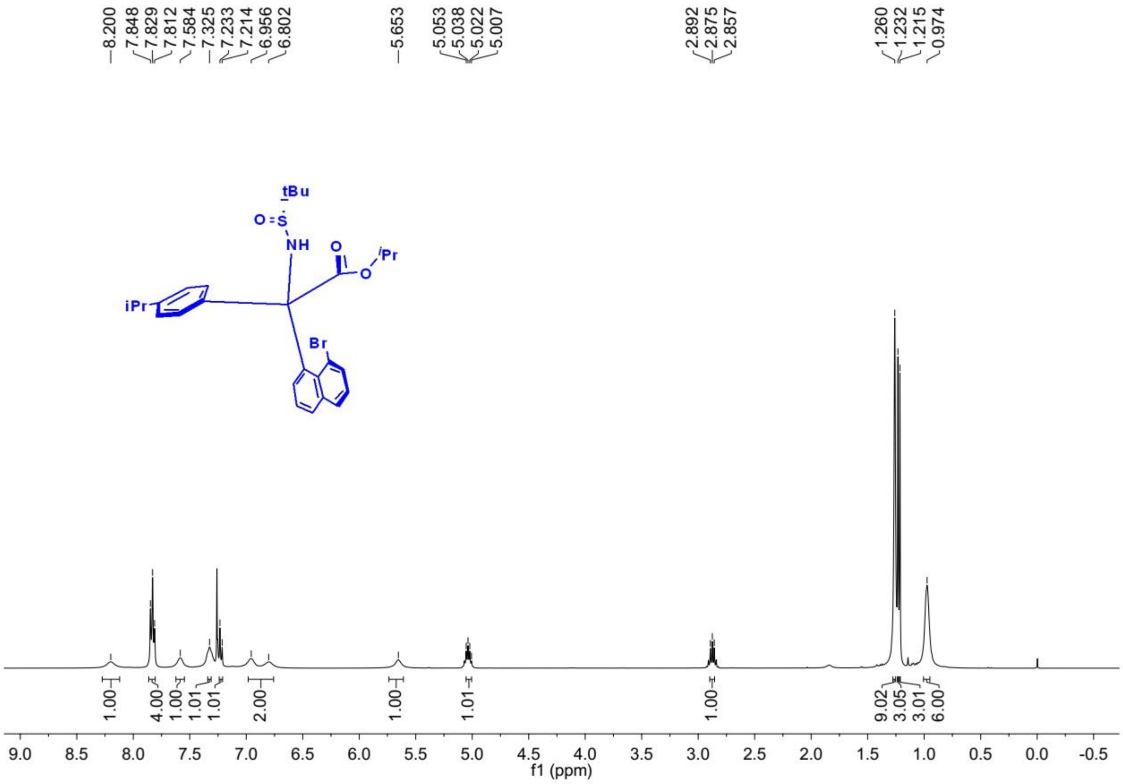

Figure 13. ^{1}**H NMR Spectrum of Compound (f)-*P*,*P*,*P* (CDCl$_3$, 400 MHz)**

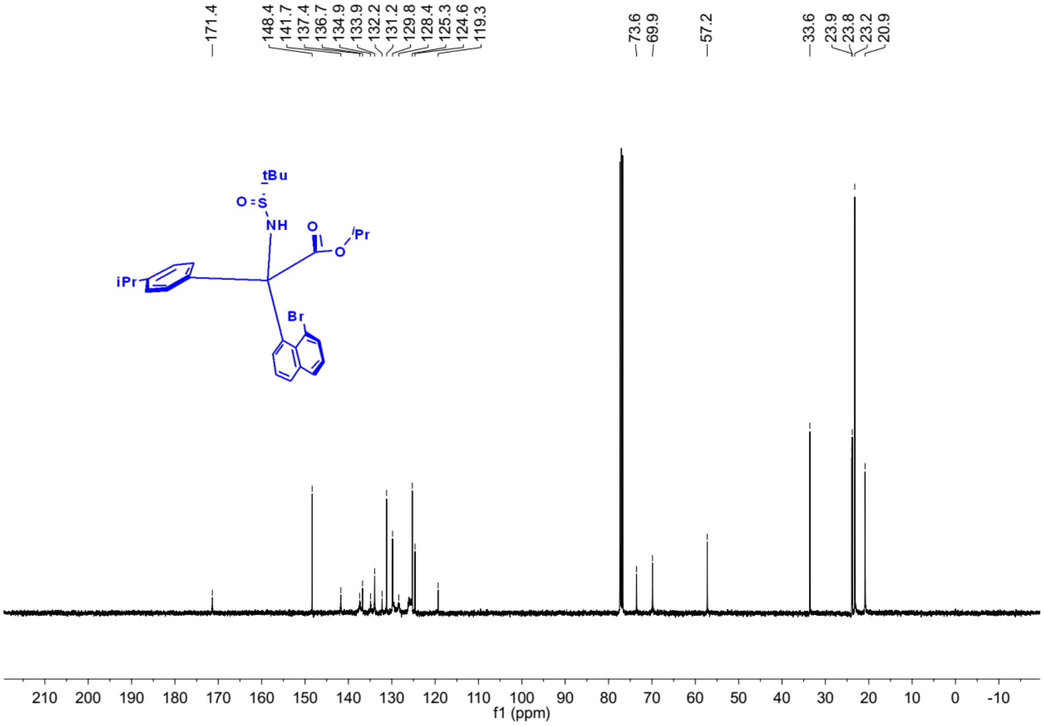

Figure 14. ^{13}**C NMR Spectrum of Compound (f)-*P*,*P*,*P* (CDCl$_3$, 100 MHz)**

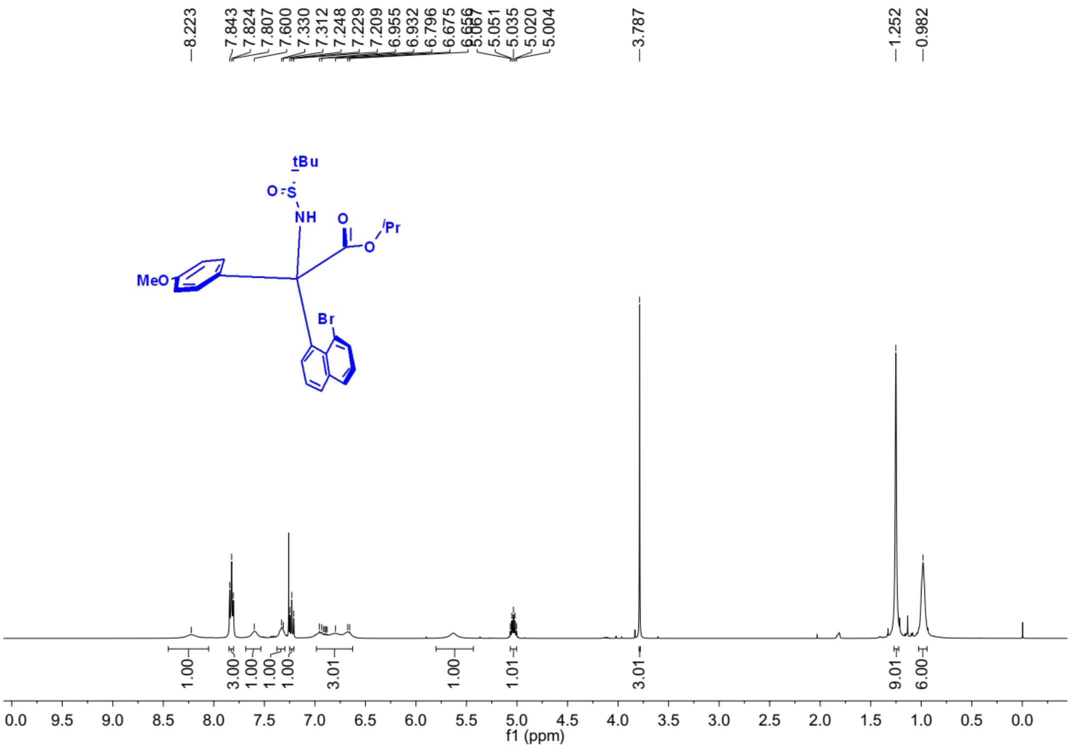

Figure 15. **^{1}H NMR Spectrum of Compound (g)-*P,P,P* (CDCl$_3$, 400 MHz)**

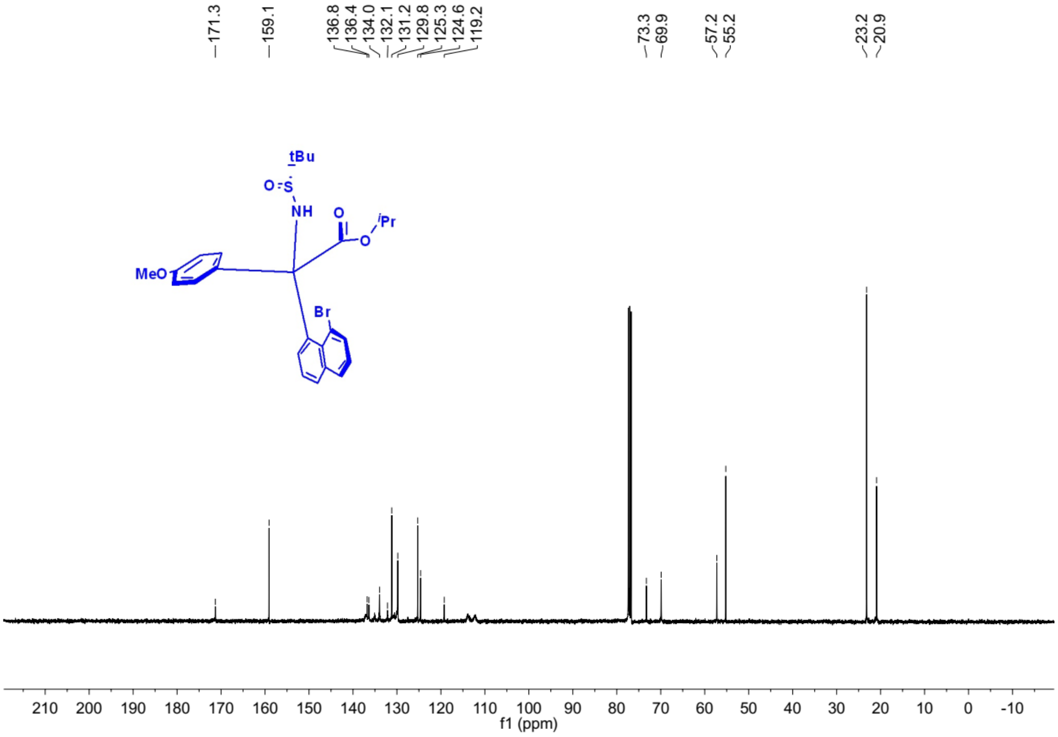

Figure 16. **^{13}C NMR Spectrum of Compound (g)-*P,P,P* (CDCl$_3$, 100 MHz)**

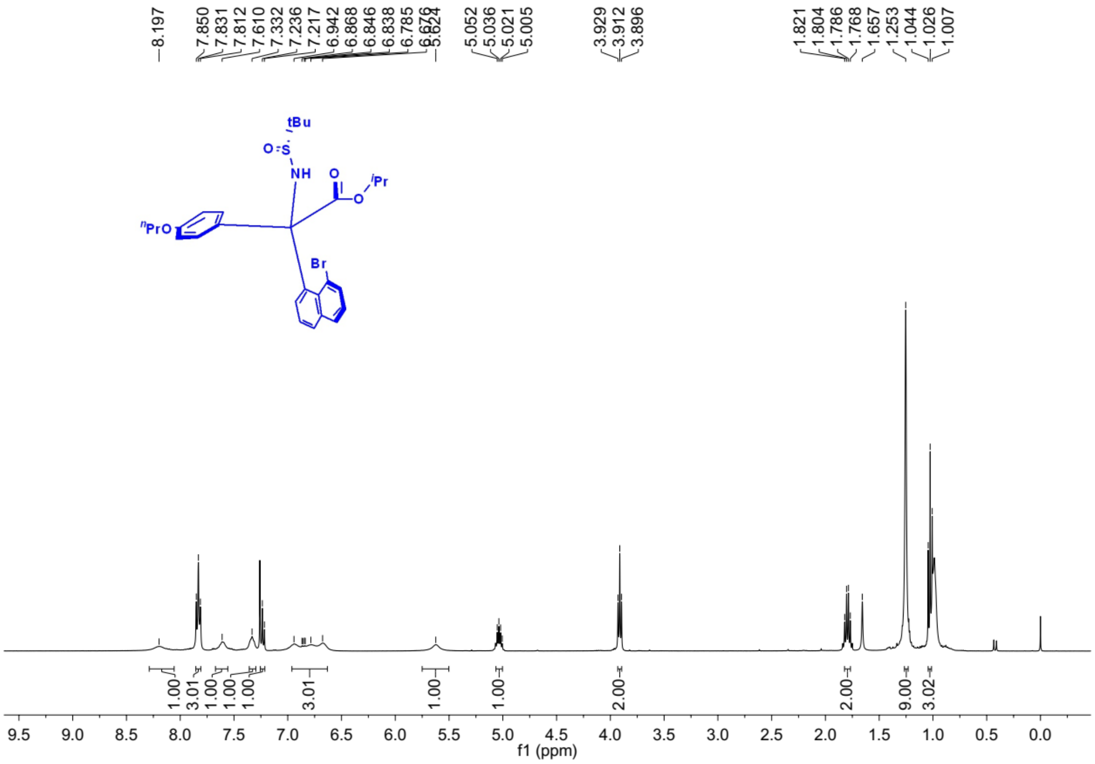

Figure 17. ^{1}**H NMR Spectrum of Compound** (**h**)-*P,P,P* **(CDCl**$_3$**, 400 MHz)**

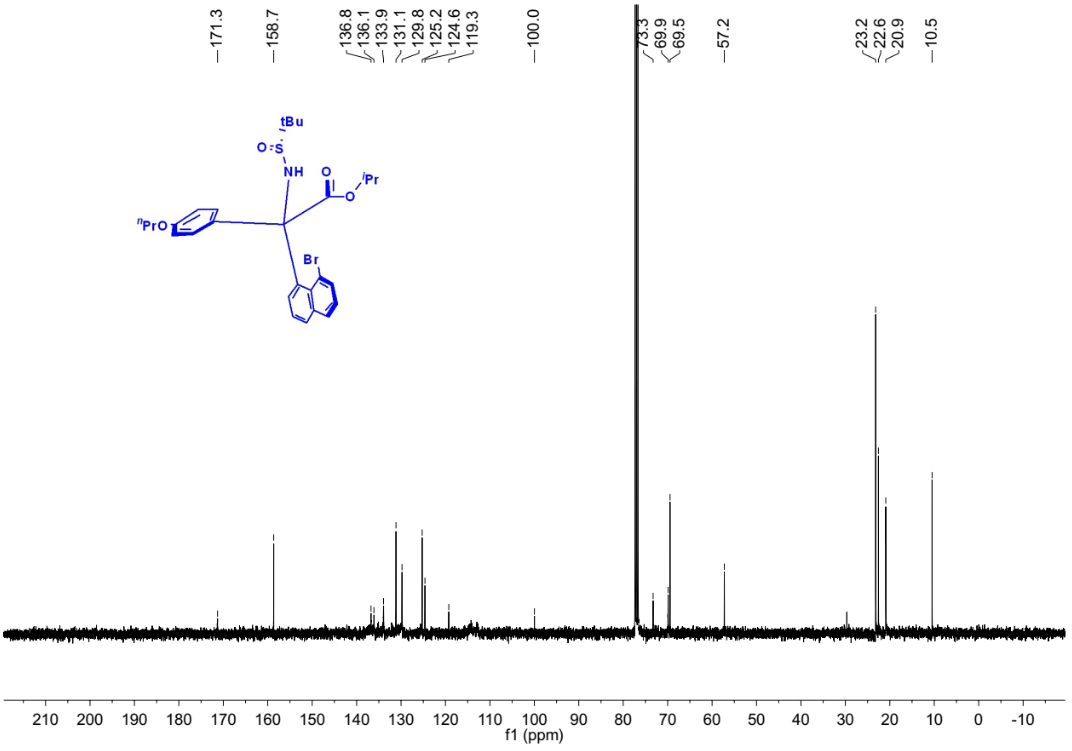

Figure 18. ^{13}**C NMR Spectrum of Compound** (**h**)-*P,P,P* **(CDCl**$_3$**, 100 MHz)**

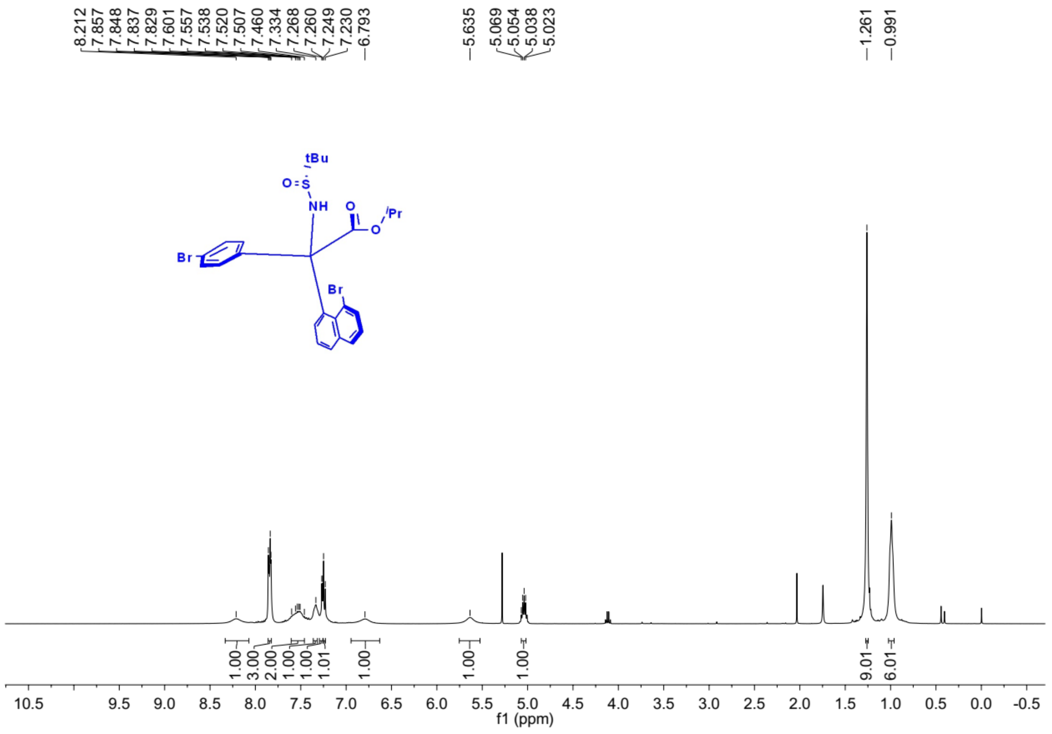

Figure 19. ^{1}H NMR Spectrum of Compound (i)-*P*,*P*,*P* ($CDCl_3$, 400 MHz)

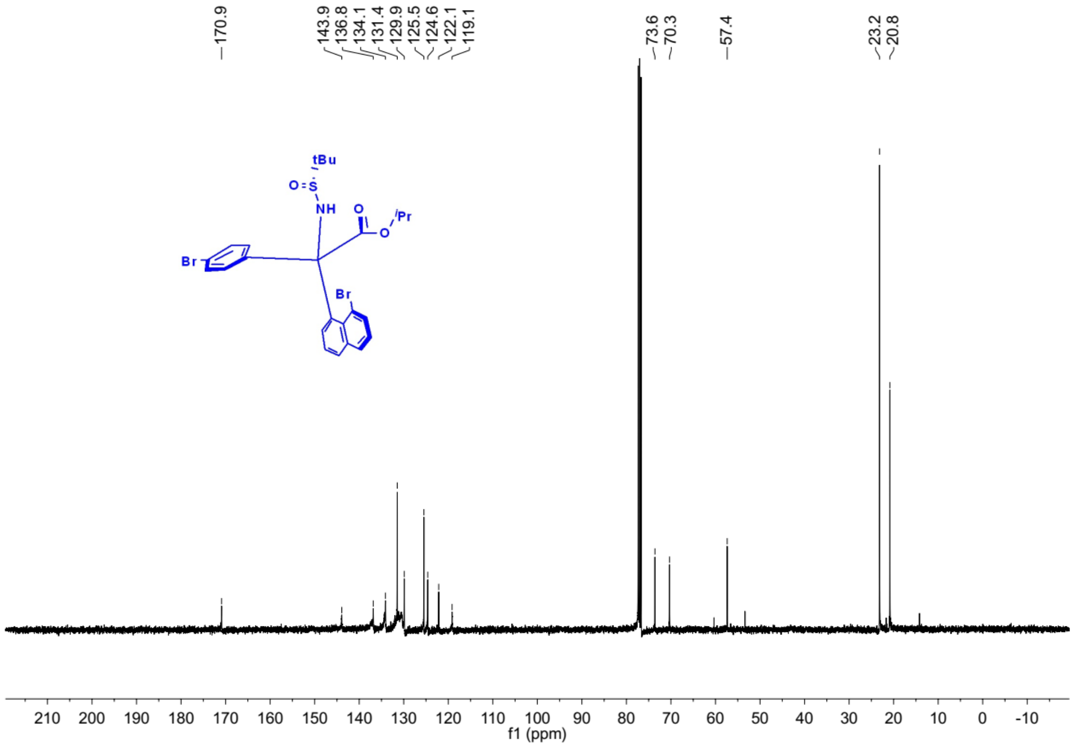

Figure 20. ^{13}C NMR Spectrum of Compound (i)-*P*,*P*,*P* ($CDCl_3$, 100 MHz)

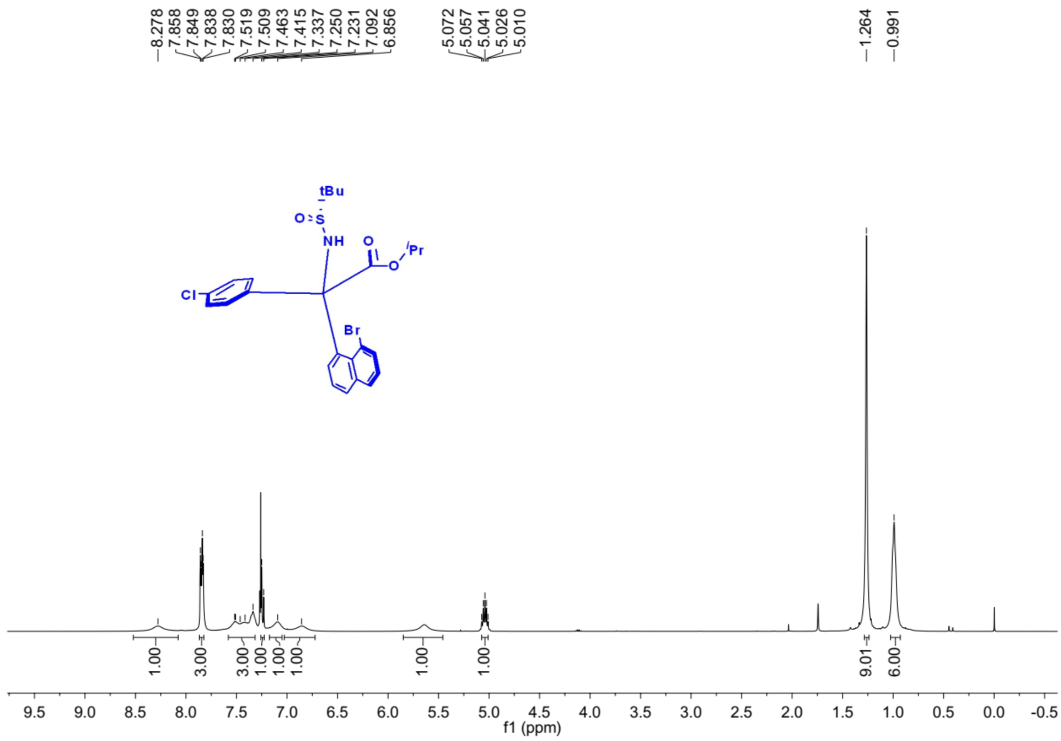

Figure 21. ^{1}**H NMR Spectrum of Compound** (**j**)-*P*,*P*,*P* **(CDCl$_3$, 400 MHz)**

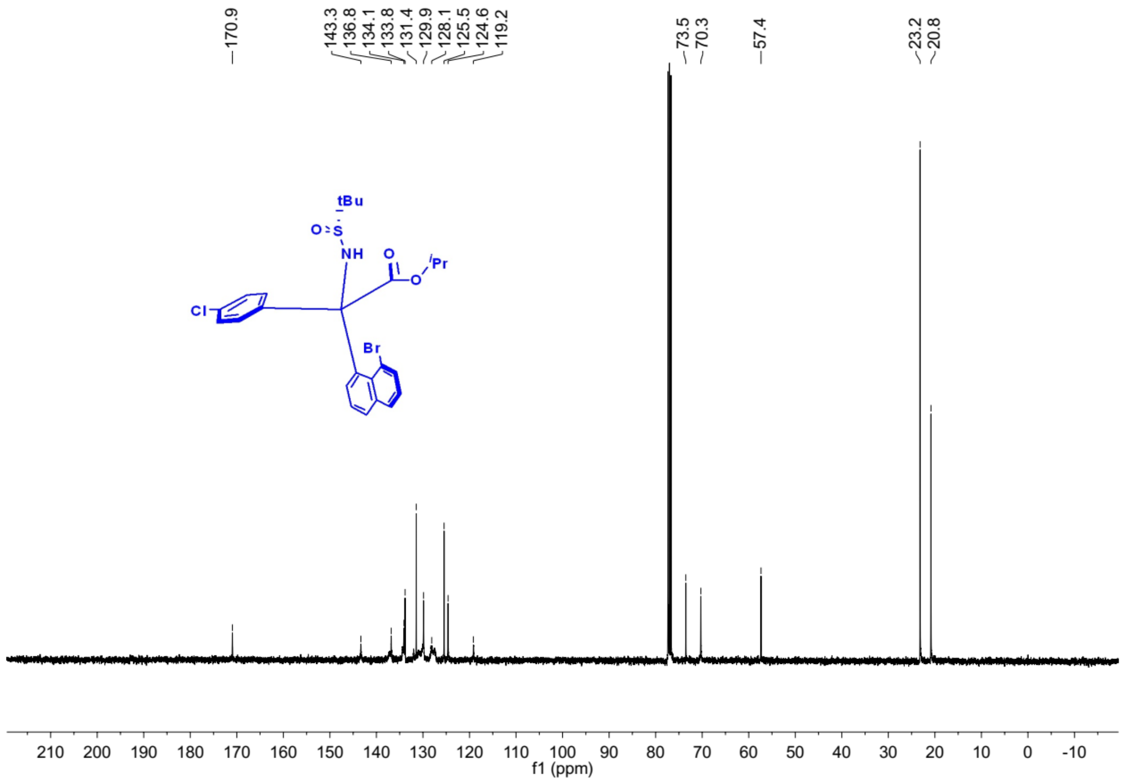

Figure 22. ^{13}**C NMR Spectrum of Compound** (**j**)-*P*,*P*,*P* **(CDCl$_3$, 100 MHz)**

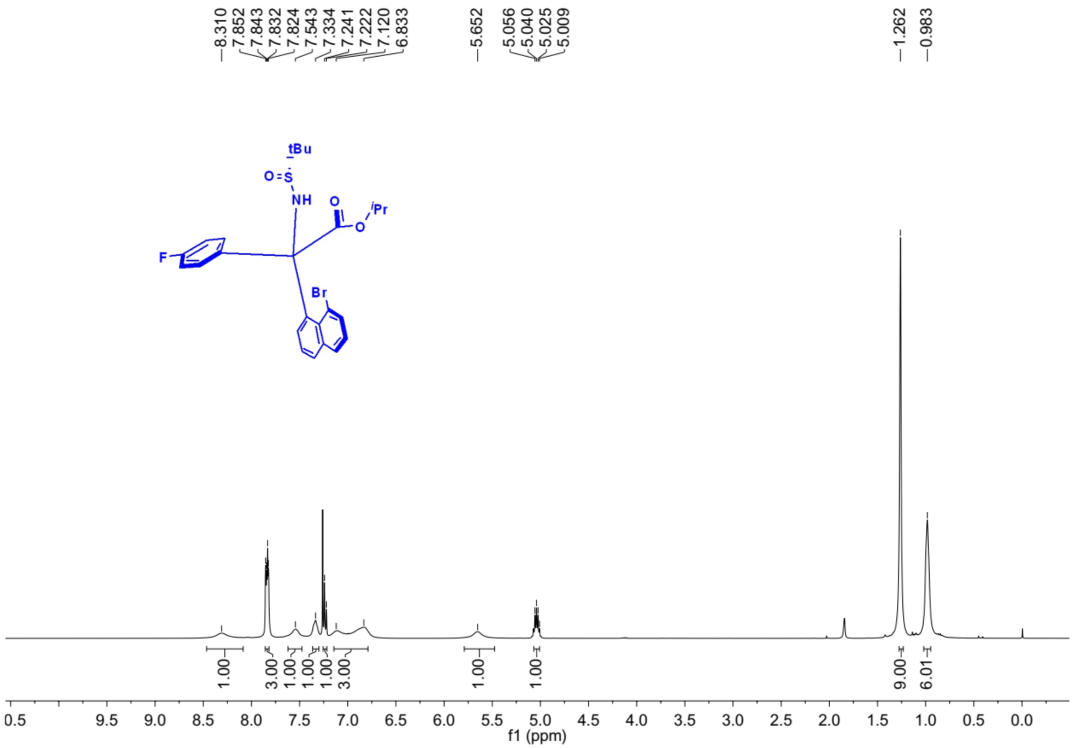

Figure 23. ^{1}**H NMR Spectrum of Compound (k)-*P,P,P* (CDCl$_3$, 400 MHz)**

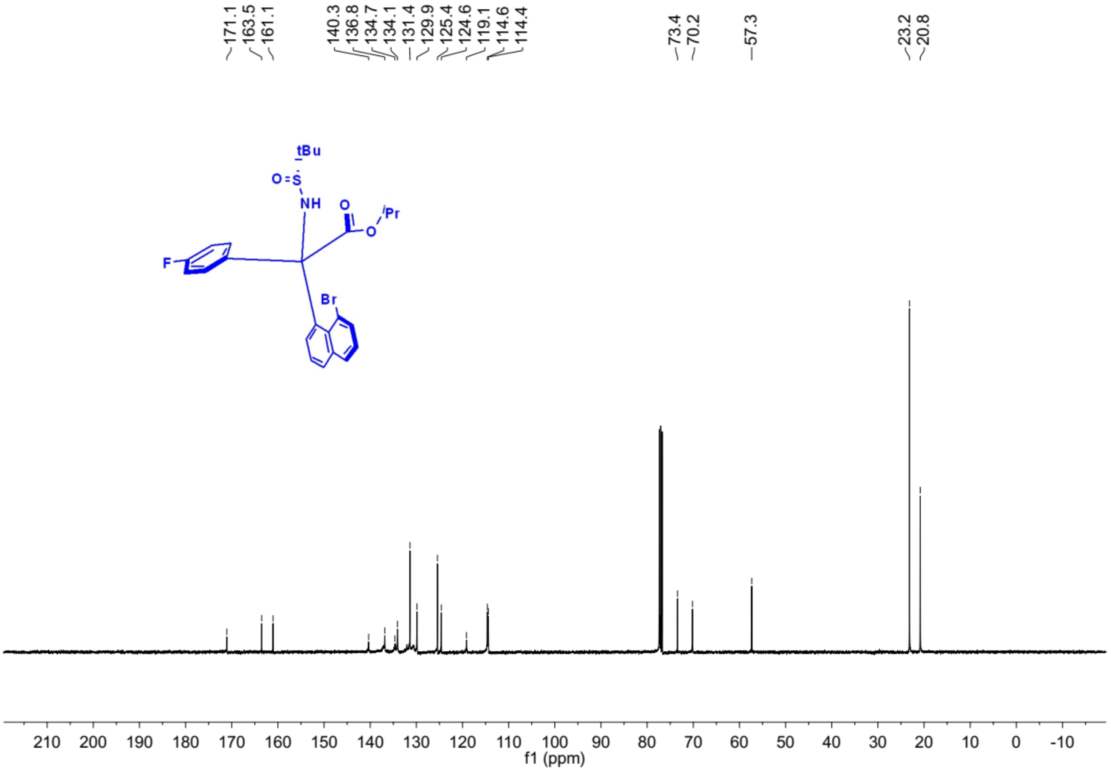

Figure 24. ^{13}**C NMR Spectrum of Compound (k)-*P,P,P* (CDCl$_3$, 100 MHz)**

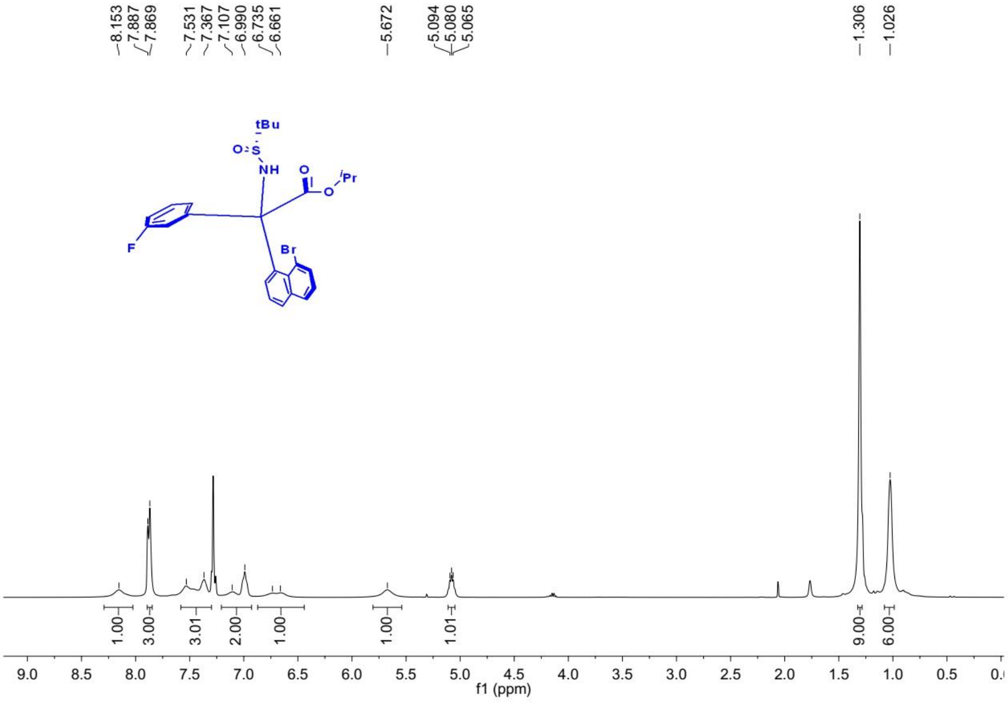

Figure 25. [1]**H NMR Spectrum of Compound** (l)-*P*,*P*,*P* **(CDCl**$_3$**, 400 MHz)**

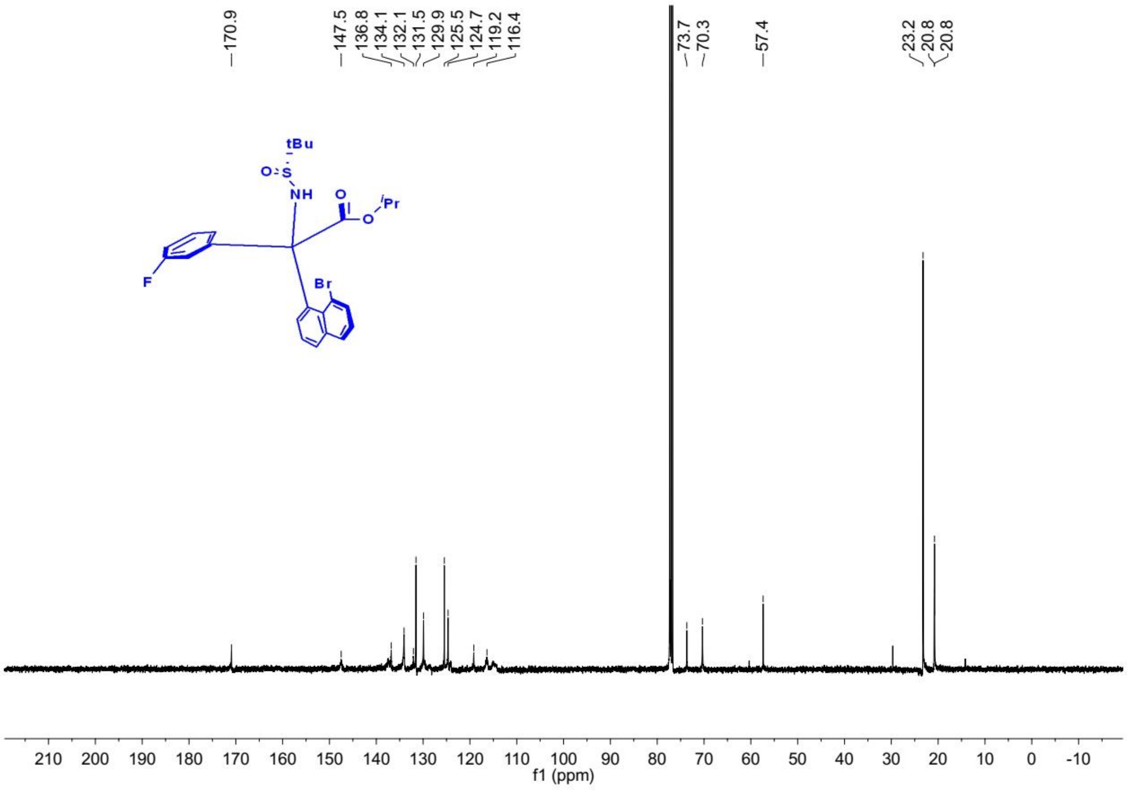

Figure 26. [13]**C NMR Spectrum of Compound** (l)-*P*,*P*,*P* **(CDCl**$_3$**, 100 MHz)**

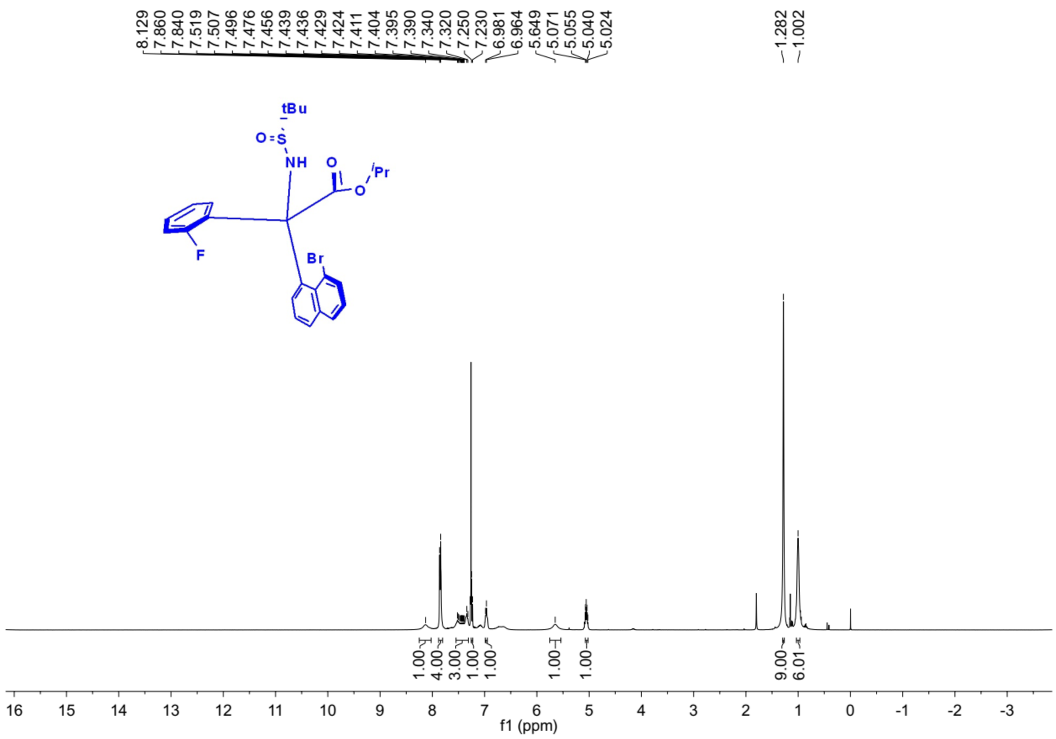

Figure 27. **^{1}H NMR Spectrum of Compound** (m)-*P*,*P*,*P* **(CDCl$_3$, 400 MHz)**

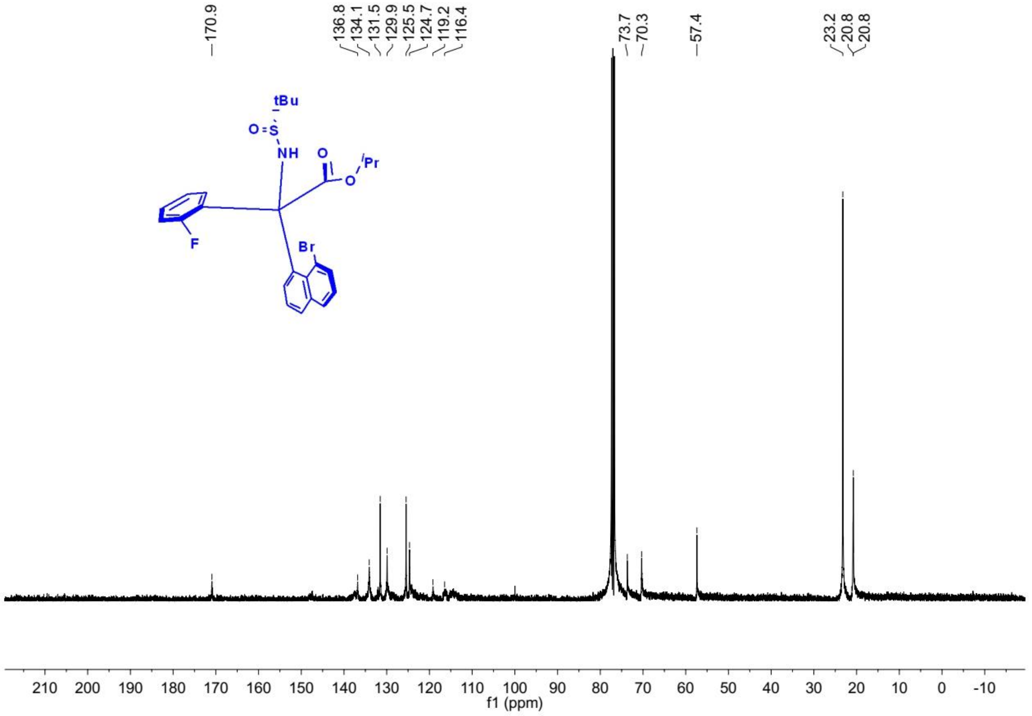

Figure 28. **^{13}C NMR Spectrum of Compound** (m)-*P*,*P*,*P* **(CDCl$_3$, 100 MHz)**

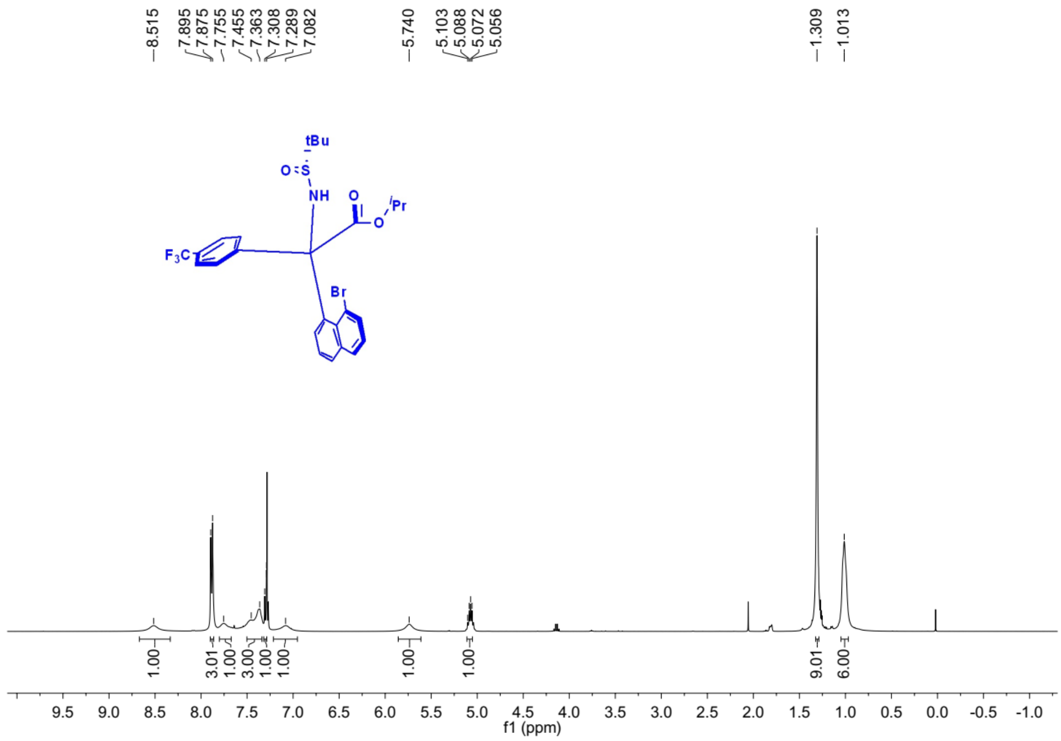

Figure 29. ^{1}H NMR Spectrum of Compound (n)-*P,P,P* (CDCl₃, 400 MHz)

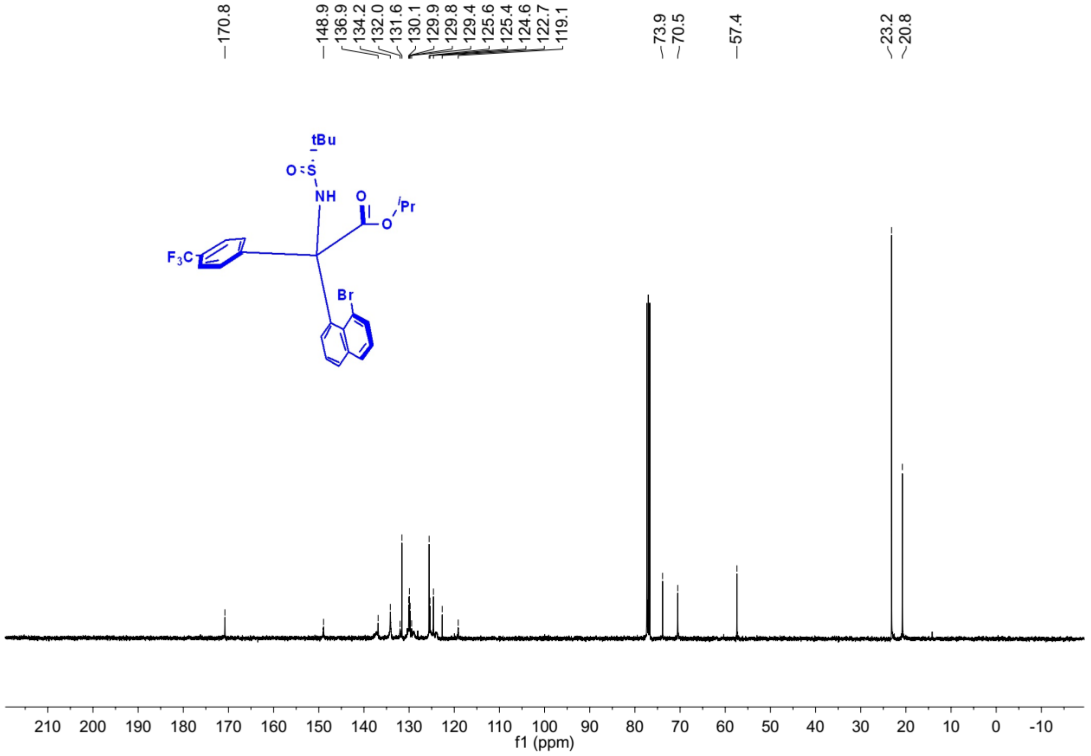

Figure 30. ^{13}C NMR Spectrum of Compound (n)-*P,P,P* (CDCl₃, 100 MHz)

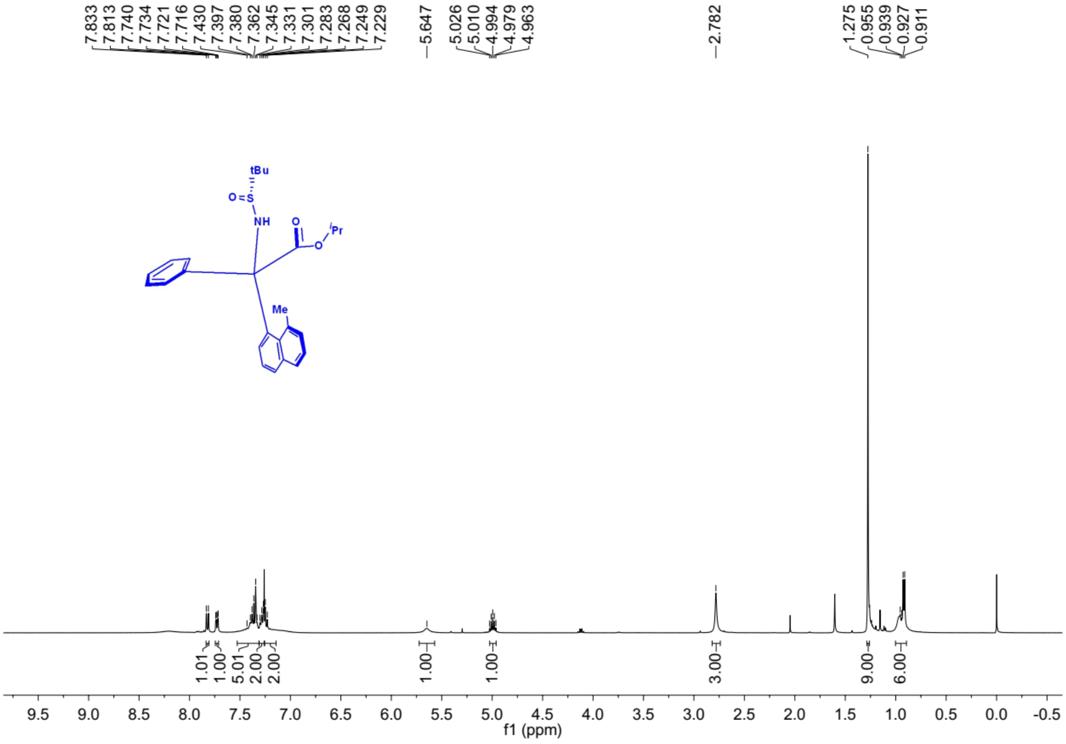

Figure 31. ^{1}**H NMR Spectrum of Compound** (**o**)-*P*,*P*,*P* **(CDCl$_3$, 400 MHz)**

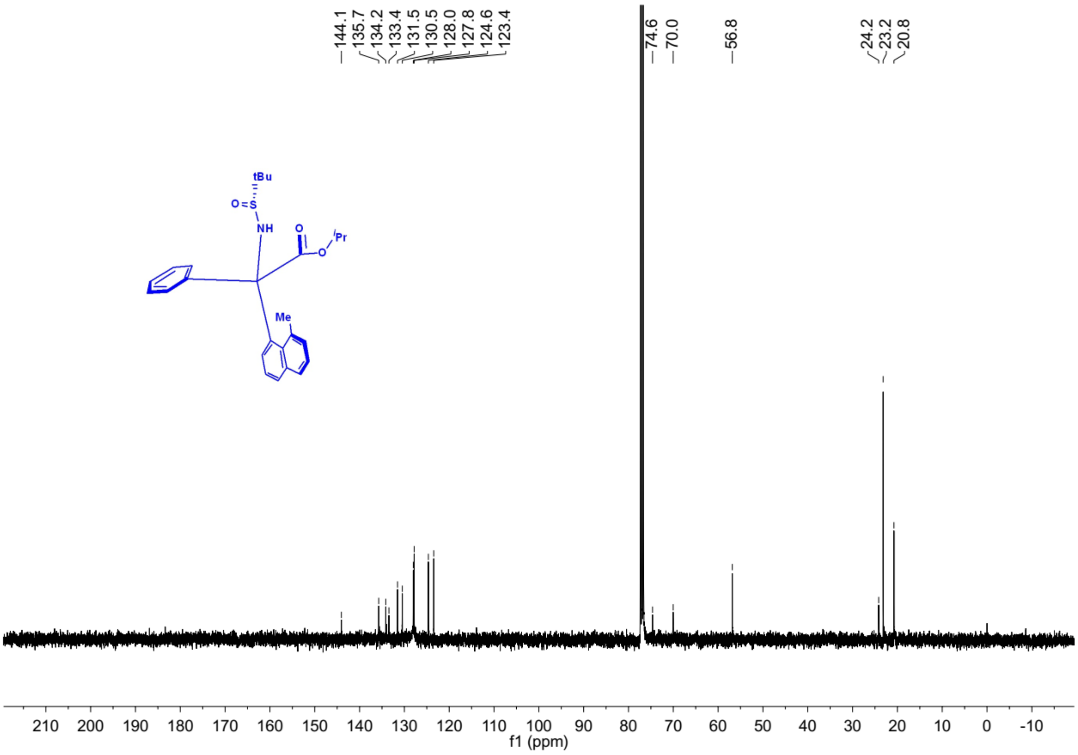

Figure 32. ^{13}**C NMR Spectrum of Compound** (**o**)-*P*,*P*,*P* **(CDCl$_3$, 100 MHz)**

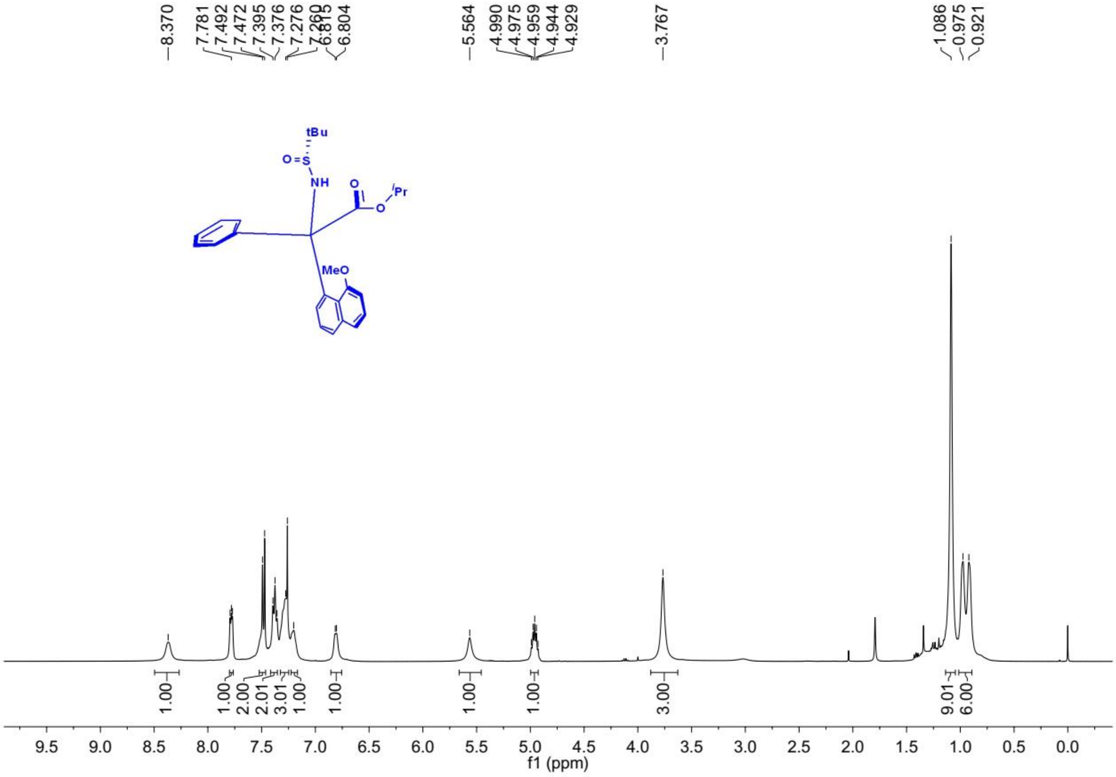

Figure 33. ^{1}H NMR Spectrum of Compound (**p**)-*P*,*P*,*P* **(CDCl$_3$, 400 MHz)**

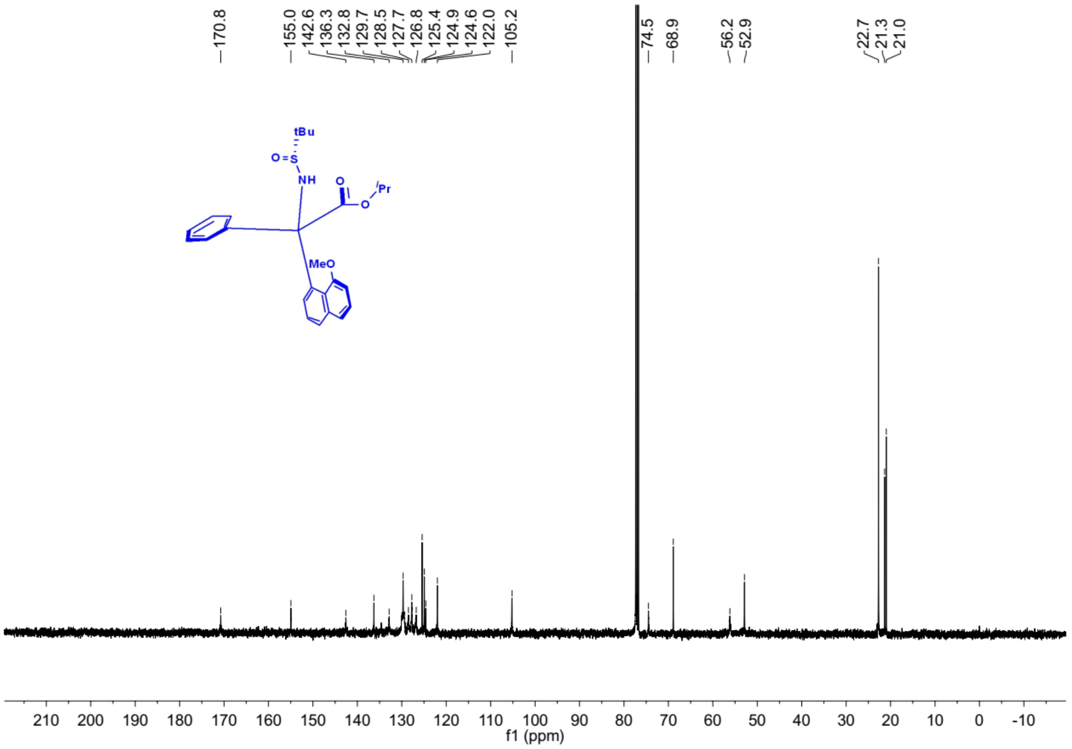

Figure 34. ^{13}C NMR Spectrum of Compound (**p**)-*P*,*P*,*P* **(CDCl$_3$, 100 MHz)**

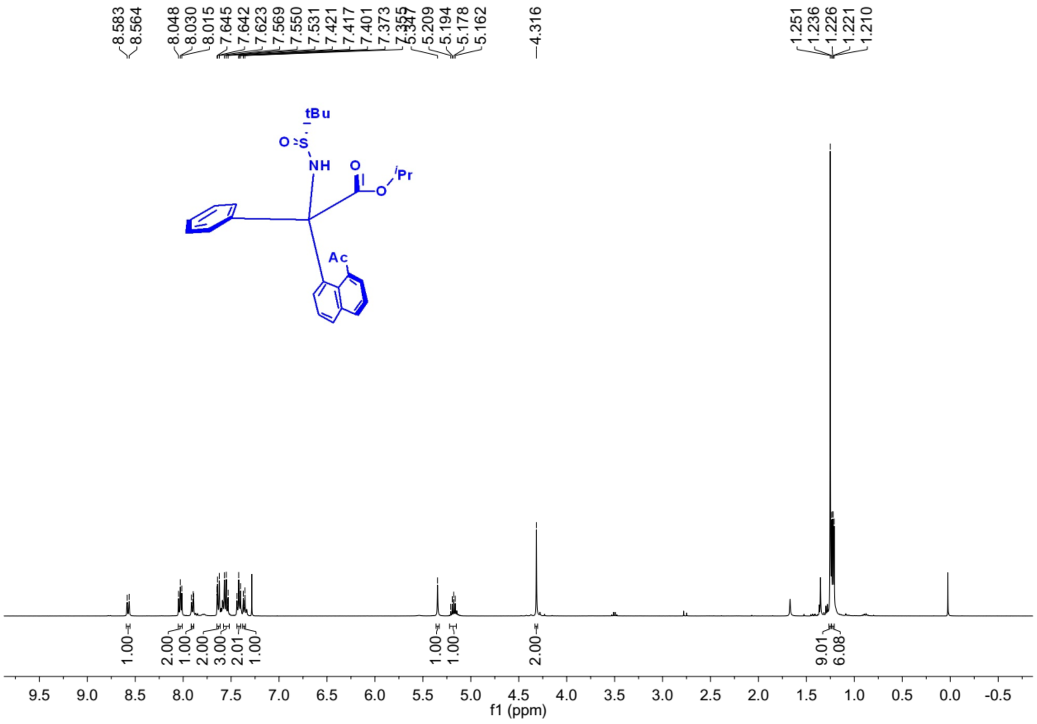

Figure 35. ^{1}H NMR Spectrum of Compound (q)-*P*,*P*,*P* ($CDCl_3$, 400 MHz)

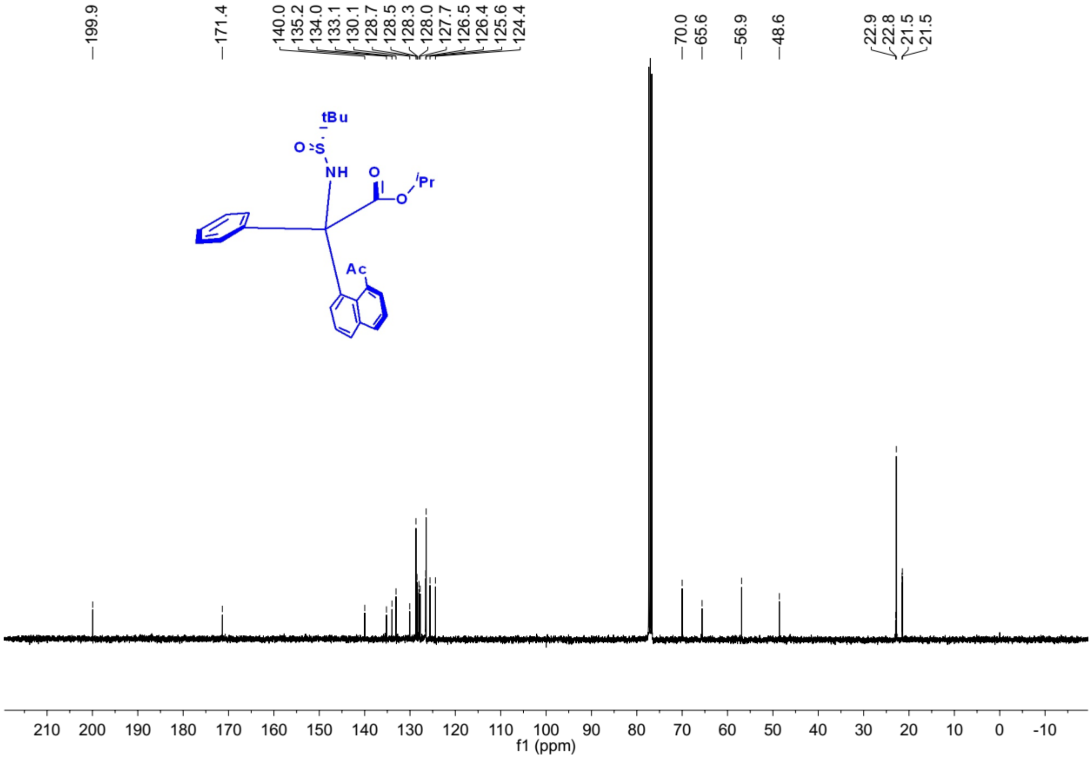

Figure 36. ^{13}C NMR Spectrum of Compound (q)-*P*,*P*,*P* ($CDCl_3$, 100 MHz)

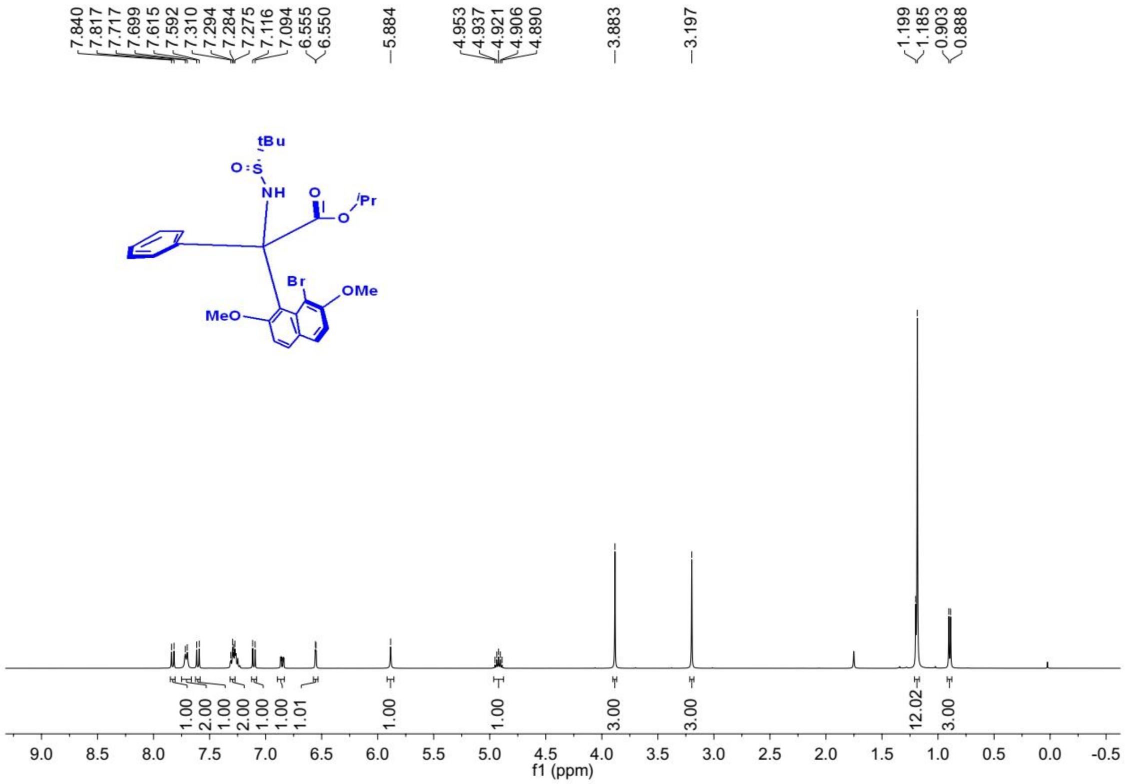

Figure 37. ^{1}H NMR Spectrum of Compound (r)-*P*,*P*,*P* ($CDCl_3$, 400 MHz)

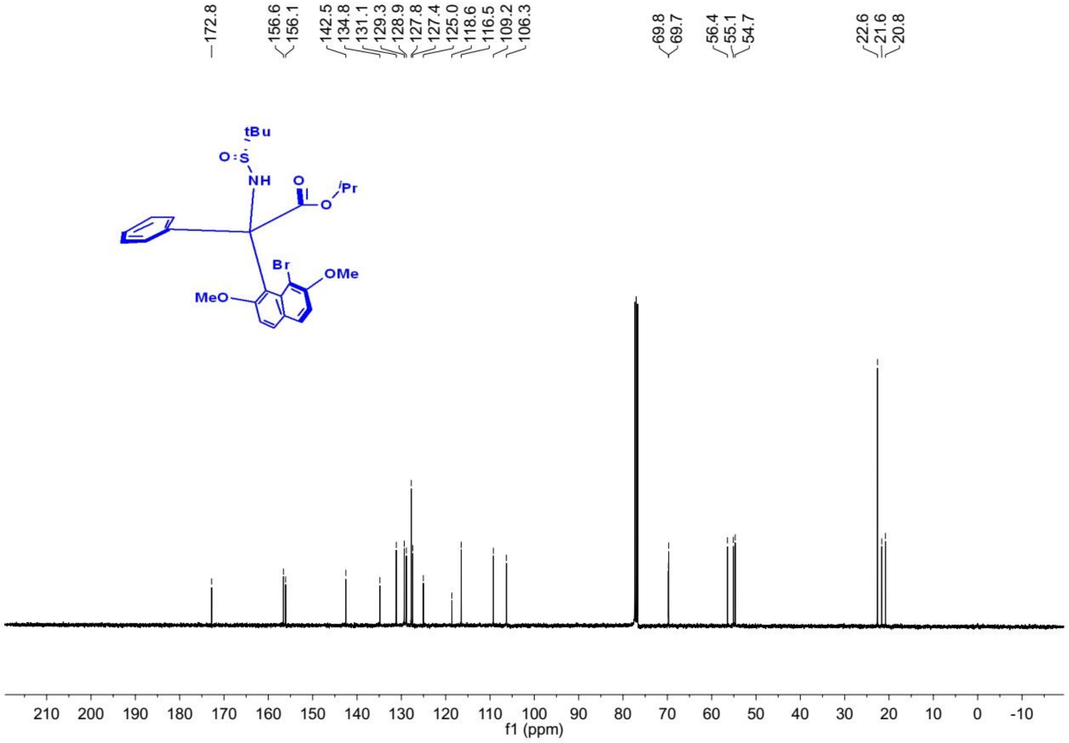

Figure 38. ^{13}C NMR Spectrum of Compound (r)-*P*,*P*,*P* ($CDCl_3$, 100 MHz)

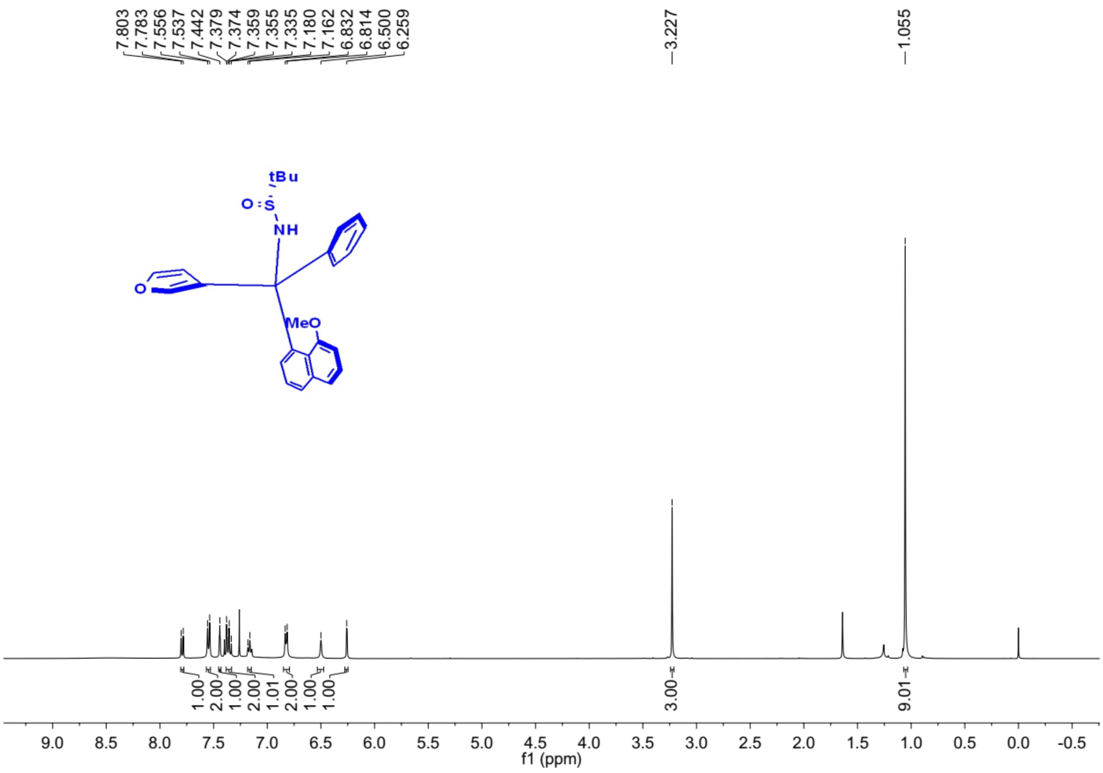

Figure 39. **^{1}H NMR Spectrum of Compound** (s)-*P*,*P*,*P* **(CDCl3, 400 MHz)**

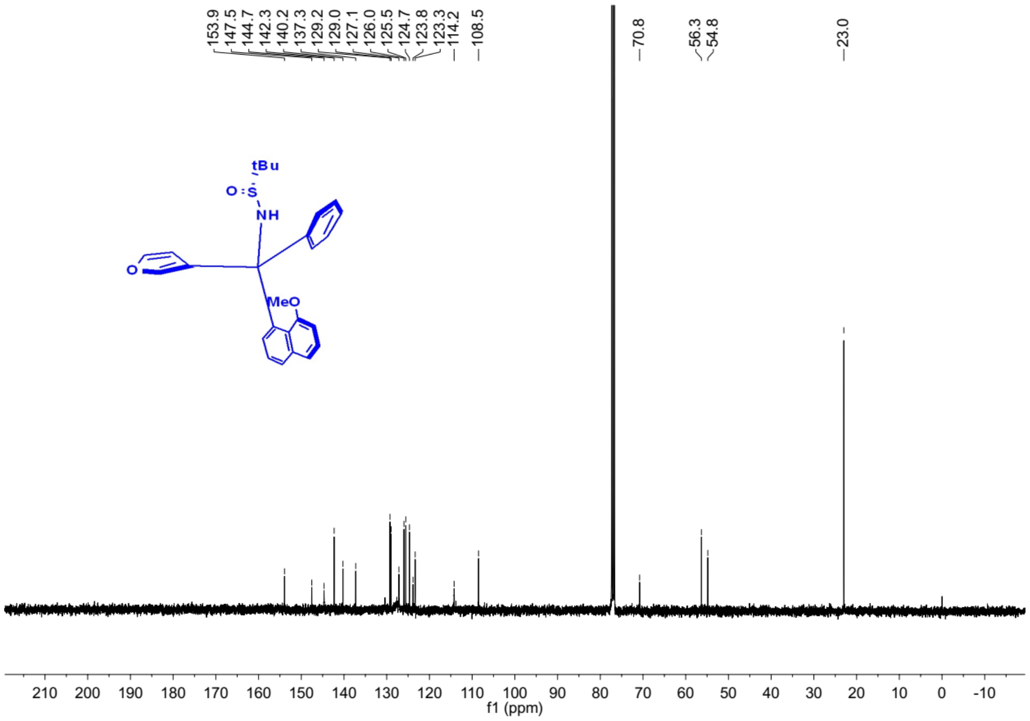

Figure 40. **^{13}C NMR Spectrum of Compound** (s)-*P*,*P*,*P* **(CDCl3, 100 MHz)**

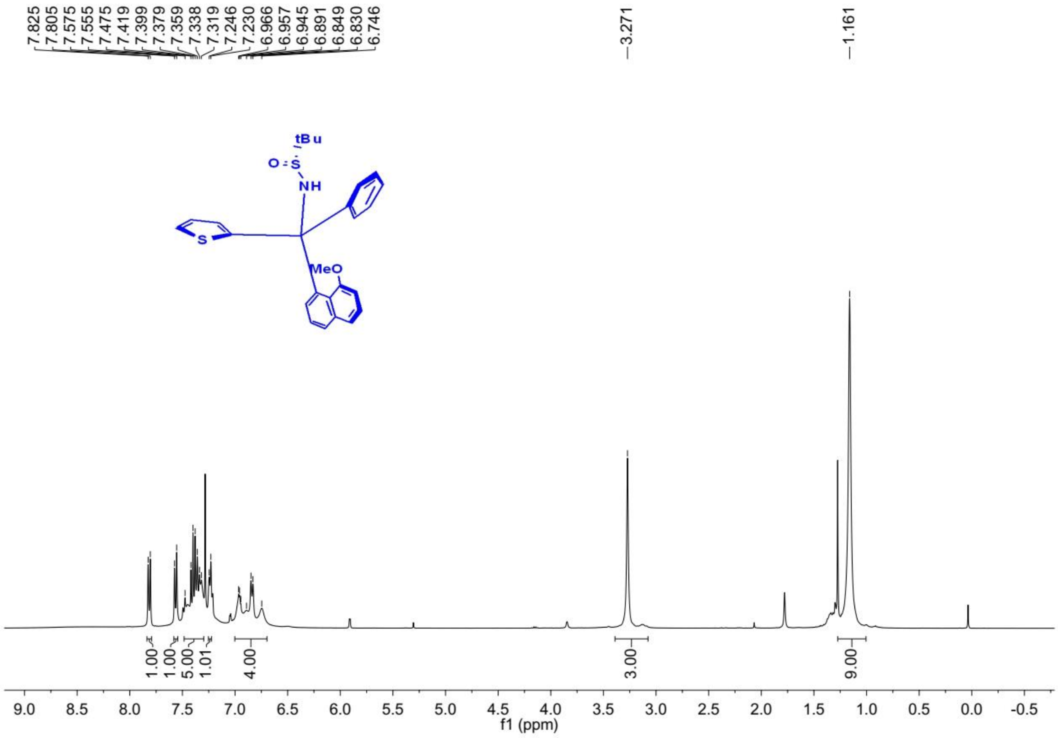

Figure 41. ^{1}**H NMR Spectrum of Compound** (**t**)-*P*,*P*,*P* **($CDCl_3$, 400 MHz)**

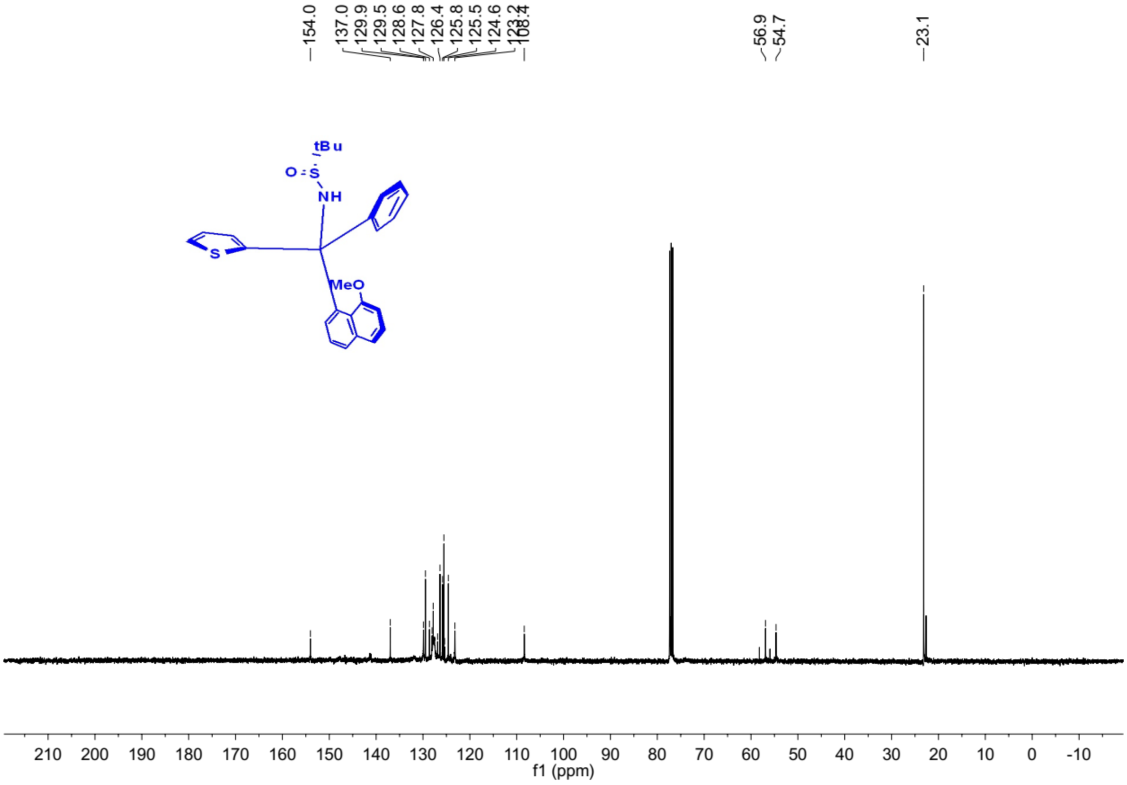

Figure 42. ^{13}**C NMR Spectrum of Compound** (**t**)-*P*,*P*,*P* **($CDCl_3$, 100 MHz)**

5. X-ray Single-crystal Data for Compound (a)-*P,P,P*, (a)-*M,M,M*, (b)-*P,P,P*, (c)-*M,M,M*, (d)-*M,M,M*, (s)-*P,P,P*, (t)-*P,P,P*

(a)-*P,P,P* : 2350832

Table 1 Crystal data and structure refinement for 2350832.

Identification code	**2350832**
Empirical formula	$C_{22}H_{20}BrN_4O_4S_2$
Formula weight	548.45
Temperature/K	298(2)
Crystal system	orthorhombic
Space group	$P2_12_12_1$
a/Å	8.8335(8)
b/Å	15.9966(15)
c/Å	16.2847(17)
α/°	90.00
β/°	90.00
γ/°	90.00
Volume/Å^3	2301.1(4)
Z	4
ρ_{calc}g/cm^3	1.583
μ/mm^{-1}	2.006
F(000)	1116.0
Crystal size/mm^3	0.2 × 0.14 × 0.1
Radiation	MoKα (λ = 0.71073)
2Θ range for data collection/°	5 to 50.04
Index ranges	-8 ≤ h ≤ 10, -18 ≤ k ≤ 18, -17 ≤ l ≤ 19
Reflections collected	11012
Independent reflections	4045 [R_{int} = 0.1051, R_{sigma} = 0.1047]
Data/restraints/parameters	4045/0/285
Goodness-of-fit on F^2	1.017
Final R indexes [I>=2σ (I)]	R_1 = 0.0507, wR_2 = 0.1046
Final R indexes [all data]	R_1 = 0.0805, wR_2 = 0.1123
Largest diff. peak/hole / e Å^{-3}	0.27/-0.72
Flack parameter	0.018(12)

(a)-*M,M,M* **: 2350835**

Table 1 Crystal data and structure refinement for 2350835

Identification code	2350835
Empirical formula	$C_{25}H_{28}BrNO_3S$
Formula weight	502.45
Temperature/K	298(2)
Crystal system	orthorhombic
Space group	$P2_12_12_1$
a/Å	8.8391(8)
b/Å	16.0216(14)
c/Å	16.3182(15)
α/°	90.00
β/°	90.00
γ/°	90.00
Volume/Å^3	2310.9(4)
Z	4
ρ_{calc}g/cm^3	1.444
μ/mm^{-1}	1.896
F(000)	1040.0
Crystal size/mm^3	0.38 × 0.22 × 0.15
Radiation	MoKα (λ = 0.71073)
2Θ range for data collection/°	5 to 50.04
Index ranges	-8 ≤ h ≤ 10, -18 ≤ k ≤ 19, -17 ≤ l ≤ 19
Reflections collected	11084
Independent reflections	4072 [R_{int} = 0.0530, R_{sigma} = 0.0702]
Data/restraints/parameters	4072/0/285
Goodness-of-fit on F^2	1.012
Final R indexes [I>=2σ (I)]	R_1 = 0.0372, wR_2 = 0.0788
Final R indexes [all data]	R_1 = 0.0539, wR_2 = 0.0823
Largest diff. peak/hole / e Å^{-3}	0.35/-0.46
Flack parameter	0.017(9)

(**b**)-*P*,*P*,*P* **: 2350837**

Table 1 Crystal data and structure refinement for 2350837.

Identification code	2350837
Empirical formula	$C_{26}H_{27}NO_3S$
Formula weight	433.55
Temperature/K	293(2)
Crystal system	monoclinic
Space group	C2
a/Å	16.0785(15)
b/Å	10.3061(8)
c/Å	13.7017(12)
α/°	90.00
β/°	99.149(4)
γ/°	90.00
Volume/Å^3	2241.6(3)
Z	4
ρ_{calc}g/cm^3	1.285
μ/mm^{-1}	0.172
F(000)	920.0
Crystal size/mm^3	0.4 × 0.3 × 0.23
Radiation	MoKα (λ = 0.71073)
2Θ range for data collection/°	4.72 to 50.02
Index ranges	$-19 \le h \le 16$, $-12 \le k \le 12$, $-16 \le l \le 13$
Reflections collected	5400
Independent reflections	3771 [R_{int} = 0.0253, R_{sigma} = 0.0471]
Data/restraints/parameters	3771/1/288
Goodness-of-fit on F^2	1.129
Final R indexes [I>=2σ (I)]	R_1 = 0.0404, wR_2 = 0.0869
Final R indexes [all data]	R_1 = 0.0492, wR_2 = 0.0900
Largest diff. peak/hole / e Å^{-3}	0.14/-0.24
Flack parameter	0.07(7)

(**c**)-*M,M,M* **: 2350838**

Table 1 Crystal data and structure refinement for 2350838.

Identification code	2350838
Empirical formula	$C_{26}H_{27}NO_2S_2$
Formula weight	449.61
Temperature/K	298(2)
Crystal system	monoclinic
Space group	C2
a/Å	16.0748(16)
b/Å	10.3329(11)
c/Å	13.8940(13)
α/°	90.00
β/°	98.466(3)
γ/°	90.00
Volume/Å^3	2282.6(4)
Z	4
ρ_{calc}g/cm^3	1.308
μ/mm^{-1}	0.257
F(000)	952.0
Crystal size/mm^3	0.32 × 0.2 × 0.11
Radiation	MoKα (λ = 0.71073)
2Θ range for data collection/°	4.7 to 50.04
Index ranges	-19 ≤ h ≤ 19, -12 ≤ k ≤ 12, -16 ≤ l ≤ 10
Reflections collected	5510
Independent reflections	3699 [R_{int} = 0.0359, R_{sigma} = 0.0740]
Data/restraints/parameters	3699/223/284
Goodness-of-fit on F^2	1.034
Final R indexes [I>=2σ (I)]	R_1 = 0.0592, wR_2 = 0.1337
Final R indexes [all data]	R_1 = 0.0777, wR_2 = 0.1416
Largest diff. peak/hole / e Å^{-3}	0.33/-0.36
Flack parameter	0.00(11)

(**d**)-*M*,*M*,*M* **: 2350842**

Table 1 Crystal data and structure refinement for 2350842.

Identification code	2350842
Empirical formula	$C_{30}H_{33}NO_2S$
Formula weight	471.63
Temperature/K	293(2)
Crystal system	triclinic
Space group	P1
a/Å	9.9080(8)
b/Å	9.9502(8)
c/Å	15.9002(12)
α/°	78.597(7)
β/°	72.713(7)
γ/°	61.180(5)
Volume/Å^3	1308.57(18)
Z	2
ρ_{calc}g/cm^3	1.197
μ/mm^{-1}	0.150
F(000)	504.0
Crystal size/mm^3	0.28 × 0.17 × 0.1
Radiation	MoKα (λ = 0.71073)
2Θ range for data collection/°	6.9 to 50
Index ranges	-11 ≤ h ≤ 11, -11 ≤ k ≤ 11, -12 ≤ l ≤ 18
Reflections collected	5692
Independent reflections	5109 [R_{int} = 0.0315, R_{sigma} = 0.0657]
Data/restraints/parameters	5109/3/625
Goodness-of-fit on F^2	1.042
Final R indexes [I>=2σ (I)]	R_1 = 0.0445, wR_2 = 0.0824
Final R indexes [all data]	R_1 = 0.0555, wR_2 = 0.0891
Largest diff. peak/hole / e Å^{-3}	0.17/-0.16
Flack parameter	0.05(7)

(s)-*P,P,P* : **2350840**

Table 1 Crystal data and structure refinement for 2350840.

Identification code	2350840
Empirical formula	$C_{29}H_{31}NOS$
Formula weight	441.61
Temperature/K	293(2)
Crystal system	orthorhombic
Space group	$P2_12_12_1$
a/Å	9.5130(5)
b/Å	11.5922(6)
c/Å	21.5032(12)
α/°	90.00
β/°	90.00
γ/°	90.00
Volume/Å^3	2371.3(2)
Z	4
ρ_{calc}g/cm^3	1.237
μ/mm^{-1}	0.158
F(000)	944.0
Crystal size/mm^3	0.22 × 0.16 × 0.11
Radiation	MoKα (λ = 0.71073)
2Θ range for data collection/°	7.04 to 50
Index ranges	-11 ≤ h ≤ 10, -13 ≤ k ≤ 13, -25 ≤ l ≤ 23
Reflections collected	18282
Independent reflections	4161 [R_{int} = 0.0358, R_{sigma} = 0.0320]
Data/restraints/parameters	4161/0/294
Goodness-of-fit on F^2	1.034
Final R indexes [I>=2σ (I)]	R_1 = 0.0370, wR_2 = 0.0799
Final R indexes [all data]	R_1 = 0.0450, wR_2 = 0.0832
Largest diff. peak/hole / e Å^{-3}	0.21/-0.22
Flack parameter	-0.03(7)

(t)-*P*,*P*,*P* : 2350839

Table 1 Crystal data and structure refinement for 2350839.

Identification code	2350839
Empirical formula	$C_{36}H_{33}NO_{1.5}S$
Formula weight	535.69
Temperature/K	293(2)
Crystal system	monoclinic
Space group	$P2_1$
a/Å	10.5289(4)
b/Å	18.0374(7)
c/Å	16.0043(5)
α/°	90.00
β/°	92.487(3)
γ/°	90.00
Volume/Å^3	3036.58(19)
Z	4
ρ_{calc}g/cm^3	1.172
μ/mm^{-1}	0.136
F(000)	1136.0
Crystal size/mm^3	0.27 × 0.25 × 0.13
Radiation	MoKα (λ = 0.71073)
2Θ range for data collection/°	6.92 to 50
Index ranges	$-12 \le h \le 12$, $-21 \le k \le 17$, $-18 \le l \le 19$
Reflections collected	23983
Independent reflections	9156 [R_{int} = 0.0300, R_{sigma} = 0.0371]
Data/restraints/parameters	9156/205/747
Goodness-of-fit on F^2	1.035
Final R indexes [I>=2σ (I)]	R_1 = 0.0519, wR_2 = 0.1309
Final R indexes [all data]	R_1 = 0.0620, wR_2 = 0.1392
Largest diff. peak/hole / e Å^{-3}	0.69/-0.26
Flack parameter	0.05(7)

6. Other atom-centered chiral turbo frameworks (a few examples)

Besides the turbo compounds described in this paper, the well-known BINAP also presents turbo chirality (Fig 43 a & b). For example, in the X-ray structure of (*S*)-BINAP isomer, one phosphorus-centered subunit has *P*-turbo chirality and another one has *M*-turbo chirality (although on the *M*-side, the naph ring is almost arranged along P-C axis). Similarly, BINAP oxide also presents turbo chirality.

In the X-ray structure of (*S*)-BINAP oxide, two phosphorus-centered subunits also present *P*- and *M*-turbo chirality, respectively. Similar turbo or propeller configurations exist in chiral phosphine ligands and their metal complexes represented by those developed by Qi-Lin Zhou and Kuiling Ding groups (Fig 43 c & d). It should be noted that turbo chirality phenomenon has not been paid much attention to although many relevant chiral compounds have appeared in literature for many research purposes.

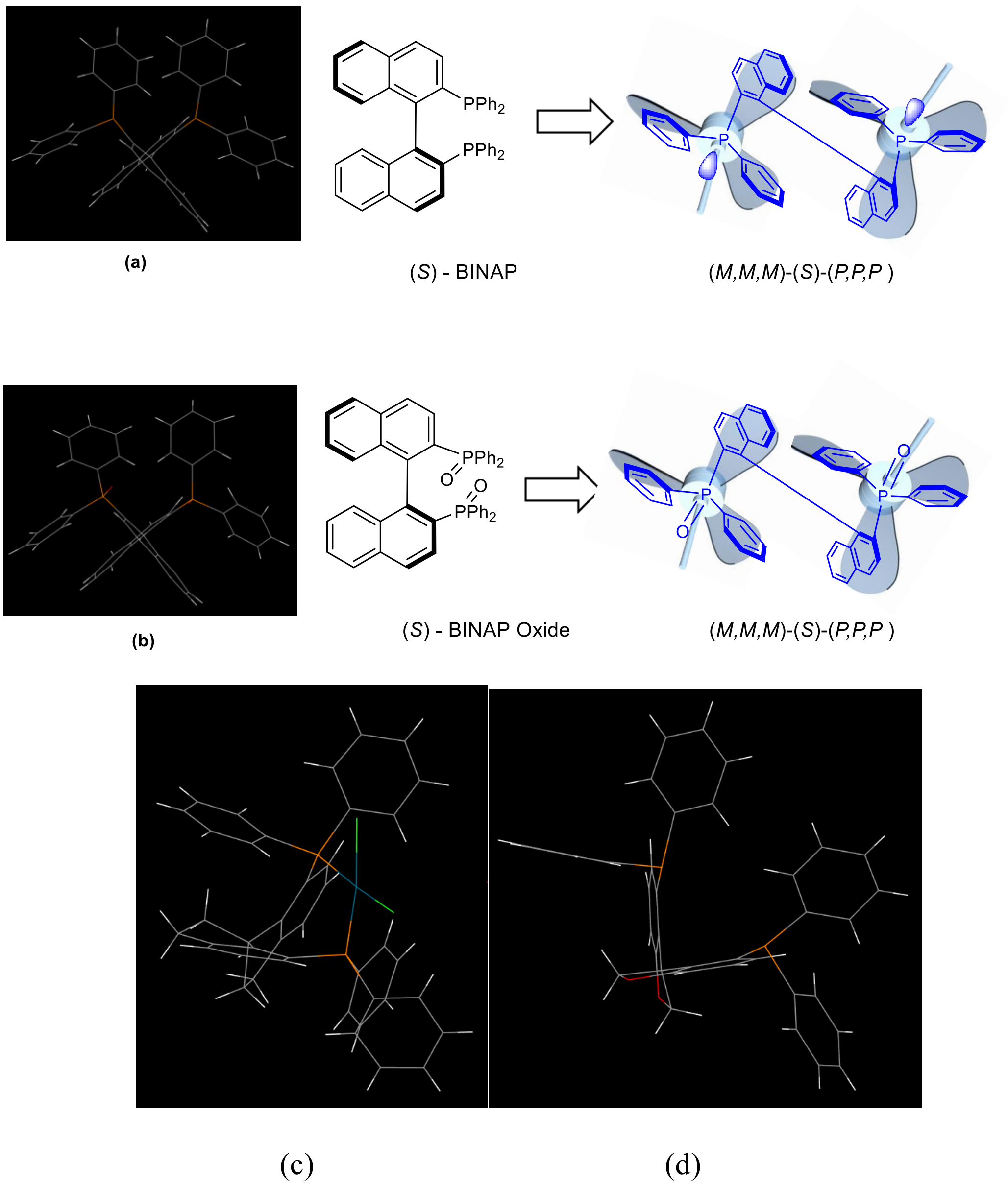

Figure 43. Turbo Chirality in BINAP, BINAP Oxide Derivatives and Pd-P Complex

In future, BINAP, BINA(P=O) and their derivatives would be noted not only simply as (*S*)- or (*R*)-configurations, but also as (*P,P,P*)- and/or -(*M,M,M*)-configurations, *e.g.*, (*P,P,P*)-(*S*)-(*M,M,M*)-BINAP [(*P,P,P*)-(*S*)-(*M,M,M*) = (*M,M,M*)-(*S*)-(*P,P,P*)] or (*P,P,P*)-(*R*)-(*M,M,M*)-BINAP to differentiate (*P,P,P*)-(*S*)-(*P,P,P*)- and (*M,M,M*)-(*S*)-(*M,M,M*)-BINAP, (*P,P,P*)-(*R*)-(*P,P,P*)- and (*M,M,M*)-(*R*)-(*M,M,M*)-BINAP, respectively. A total of eight isomers could exist theoretically if the propeller blades can be fixed by changing suitable groups surrounding P center. So far, chiral BINAP-derived ligands have been based on two configurations: (*P,P,P*)-(*S*)-(*M,M,M*) and (*P,P,P*)-(*R*)-(*M,M,M*), as reported and extensively studied ignoring other six possible isomers which would also play various important roles for asymmetric chemistry in future. It should be noted that chiral center-containing mono phosphine ligands and metal-ligand complexes also indicates turbo chirality in their structures. Examples include those developed by Guigen Li (Fig 44 a) and Junliang Zhang groups (Fig 44 b & c). Interestingly, in Zhang's ligand, phosphine and its oxide showed different turbo chirality when the same sulfonamide auxiliary is used, *i.e.*, *P,P,P*-configuration was found in the former, but *M,M,M*-configuration in the latter. This could indicate when chiral phosphine ligands are coordinated onto metals, the P-centered turbo chirality can be reversed having impacts on asymmetric control.

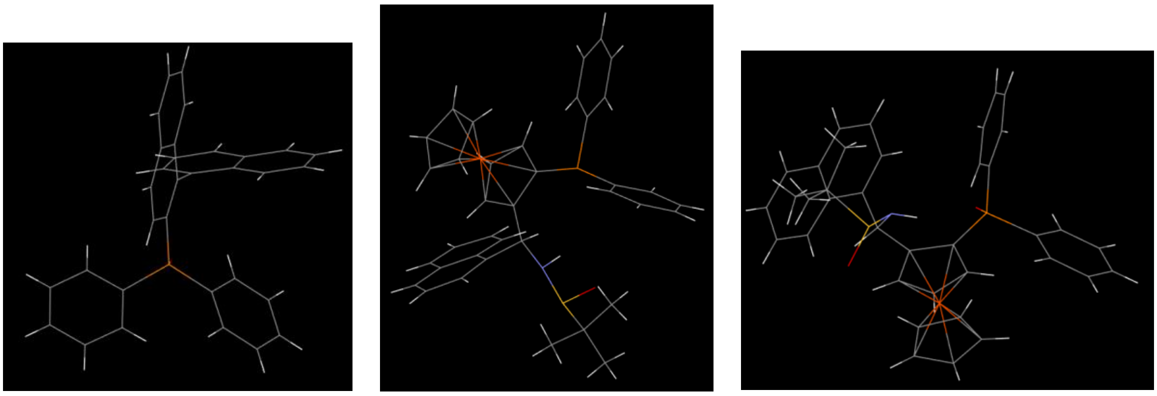

(a) P(=O) center, (*S*)-diaxial-*M*-turbo; (b) P-center, (*R*)-Sulfur, *P*-turbo; (c) P(=O)center, (*R*)-Sulfur, *M*-turbo

Figure 44. Turbo Chirality in Mono Phosphines and Oxide Derivatives

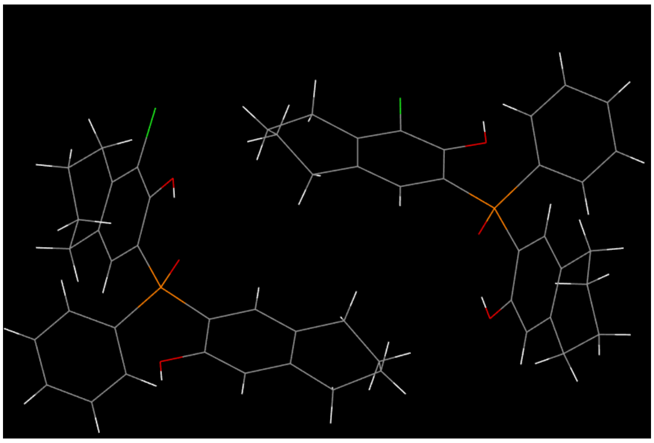

Figure 45. Two Turbo Chiral Configurations of the Same P-Center

In Xiaodan Zhao' products (Figure 45), we realized that the same chiral P center displayed two different turbo chirality with three different propeller blades. In this case, one of the aromatic rings is arranged along the C(sp2)-P axis with *M,M*- and *P,P*-configurations, respectively.

In addition, turbo chirality also exists in dual-axial chiral targets, *e.g.,* in diaryl ethers. Our careful analysis of X-ray structures by Guofu Zhong/Xiaofei Zeng and Xiaoyu Yang groups revealed that two wings can be fixed in *M,M*- or *P,P*-configuration when an axis is placed in the plane of two O-C(sp2) bonds in which the bond angle of C(sp2)-O-C(sp2) is equally divided by the axis as shown in Figure 46.

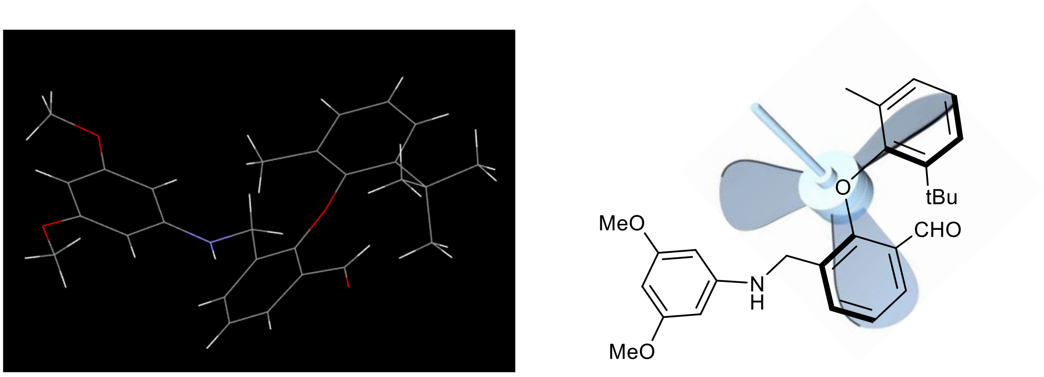

(a)

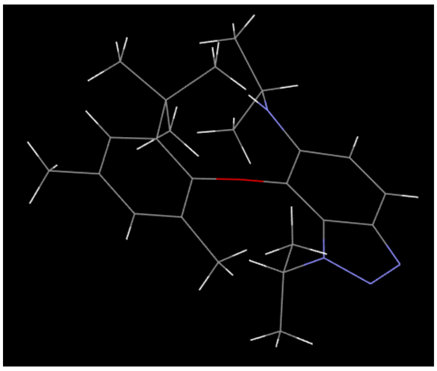

(b)

Figure 46. Turbo Chirality in Diaryl Ethers

A series of other turbo chiral targets have been designed and summarized in Figure 47. The turbo centers include P, C, S, N and Si atoms. Although phosphine oxides are used as examples, their phosphine derivatives are obviously anticipated to show turbo chirality frameworks. Bis(2,7-dimethoxynaphthalen-1-yl)phosphine oxide and derivatives with various RO groups clearly indicated turbo chirality with (*M,M,M*)- or (*P,P,P*)-configurations as our preliminary results showed. Many of their racemic products have been readily synthesized and reported in literature, relevant turbo or propeller enantiomers shown in Figure 47 are expected to be generated with great feasibility.

Finally, tertiary carbon-centered turbo alcohols and quaternary carbon-centered turbo chiral targets can also be found in literature (Figure 48). For the former, axial auxiliary can well direct turbo configuration in which a (*S*)- BINOL axial structure leads to *P,P,P*-configuration for both Ar_3C-OH moieties (Figure 48a). For the latter by Jianwei Sun, a (*S*)-carbon center directs *M,M,M*-turbo configuration if the atomically smallest Ph group is arranged along the C(sp3)-C(sp2) axis symmetrically (Figure 48b). If sterically smallest pyyrole group is arranged along another C(sp3)-C(sp2) axis, the *P,P,P*-turbo configuration is shown for the same chiral center.

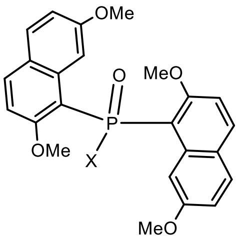

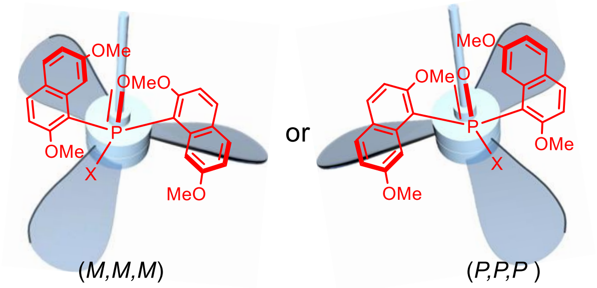

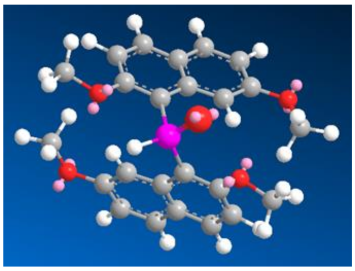

X = R, Ar, halo, H, OH, OR, NHR, NR^2, etc

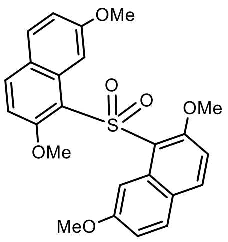

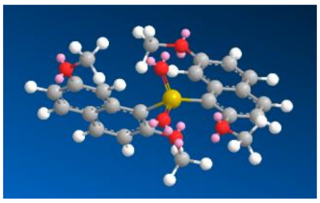

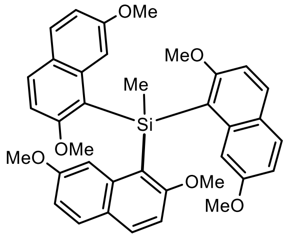

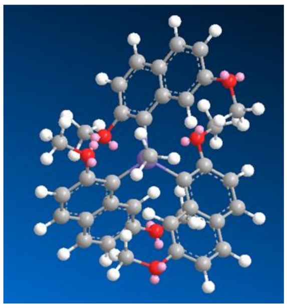

H on position 1 of naph can be Br, Me, Ph and other groups

Me can be other alkyl and Ar groups

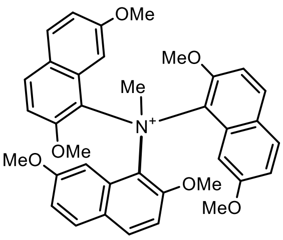

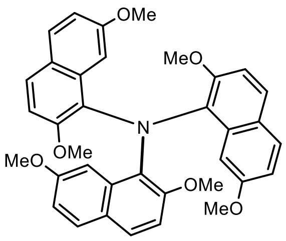

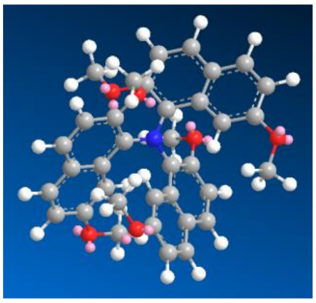

Me and naph rings can be other Ar groups; H on position 1 of naph can be Br, Me, Ph and other groups

Figure 47.

Newly Designed Turbo Chiral Targets with Various Atom Centers and Propeller Blades

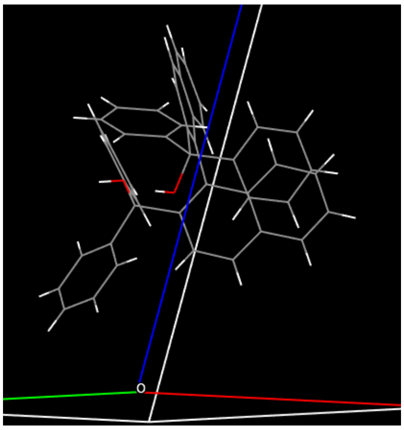

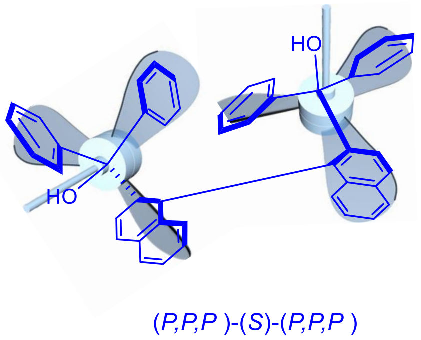

(a)

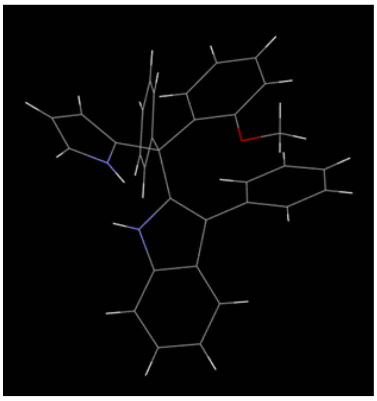

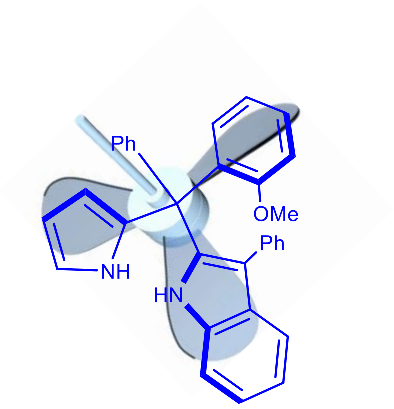

(*S*)-*M*,*M*,*M*

(b)

Figure 48. Turbo Chirality in Tertiary and Quaternary Carbon-Centered Targets

(All X-ray shown in Figure 43 - 48 can be found in literature with citations in main text and PI's name indicated on relevant writing, but none of these papers pay attention to turbo or propeller chirality)
